# Supplementary material for: Psychological distress among Japanese high school students during the COVID-19 pandemic: An energy landscape analysis
Source: PLoS Med. 2026 Jan 22;23(1):e1004884. doi: 10.1371/journal.pmed.1004884 (PMC12826503; doi:10.1371/journal.pmed.1004884)
Supplement: S1 Fig — (DOCX) [file pmed.1004884.s001.docx]

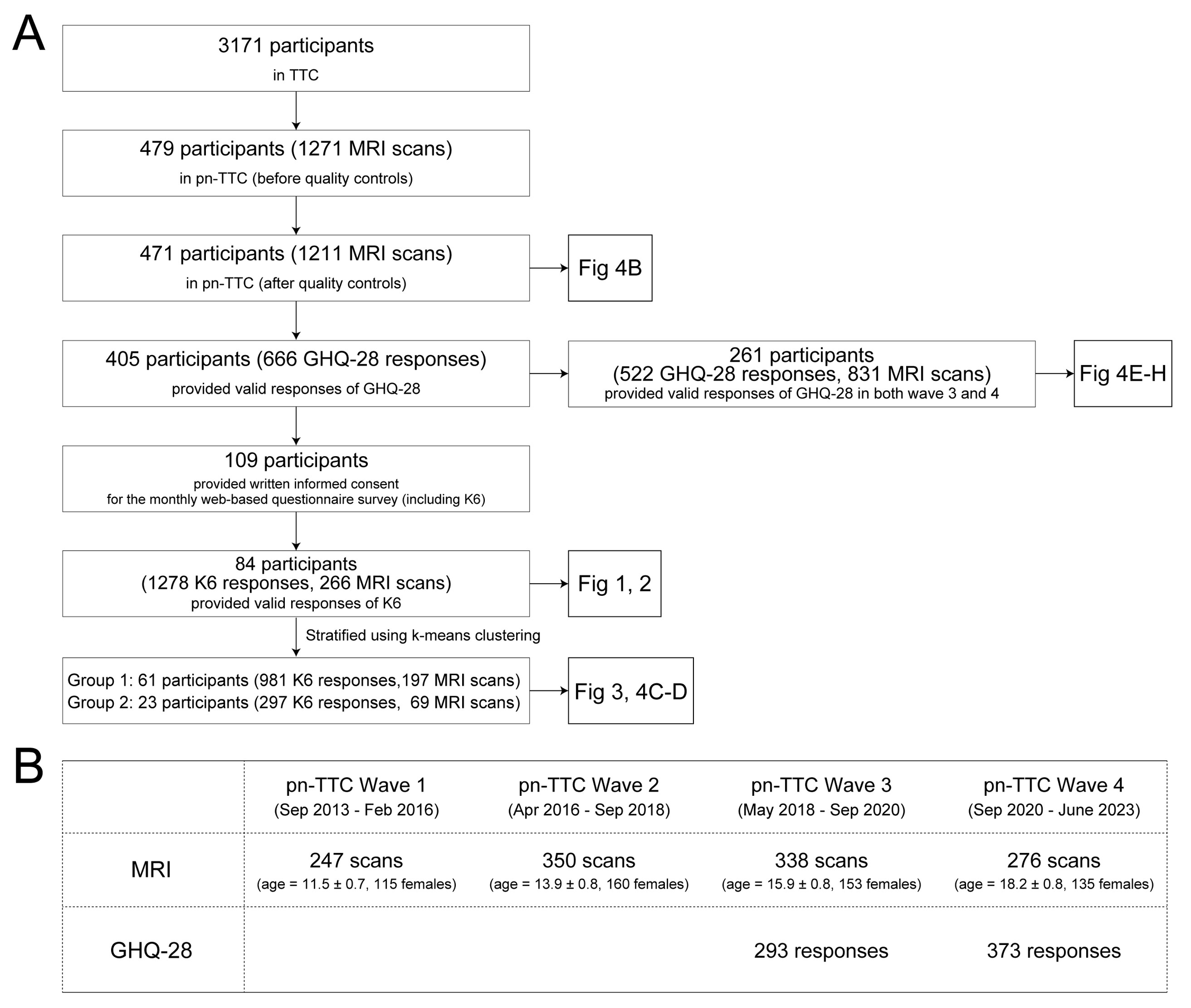


**S1 Fig** | **Summary of the whole study**: **(A)** Study flowchart showing the number of participants and the inclusion criteria for our analysis. **(B)** The study timeline and data collected during the pn-TTC. GHQ-28 = 28-item version of the General Health Questionnaire; pn-TTC = population-neuroscience Tokyo TEEN Cohort.
