# Supplementary material for: Psychological distress among Japanese high school students during the COVID-19 pandemic: An energy landscape analysis
Source: PLoS Med. 2026 Jan 22;23(1):e1004884. doi: 10.1371/journal.pmed.1004884 (PMC12826503; doi:10.1371/journal.pmed.1004884)
Supplement: S2 Fig — (DOCX) [file pmed.1004884.s002.docx]

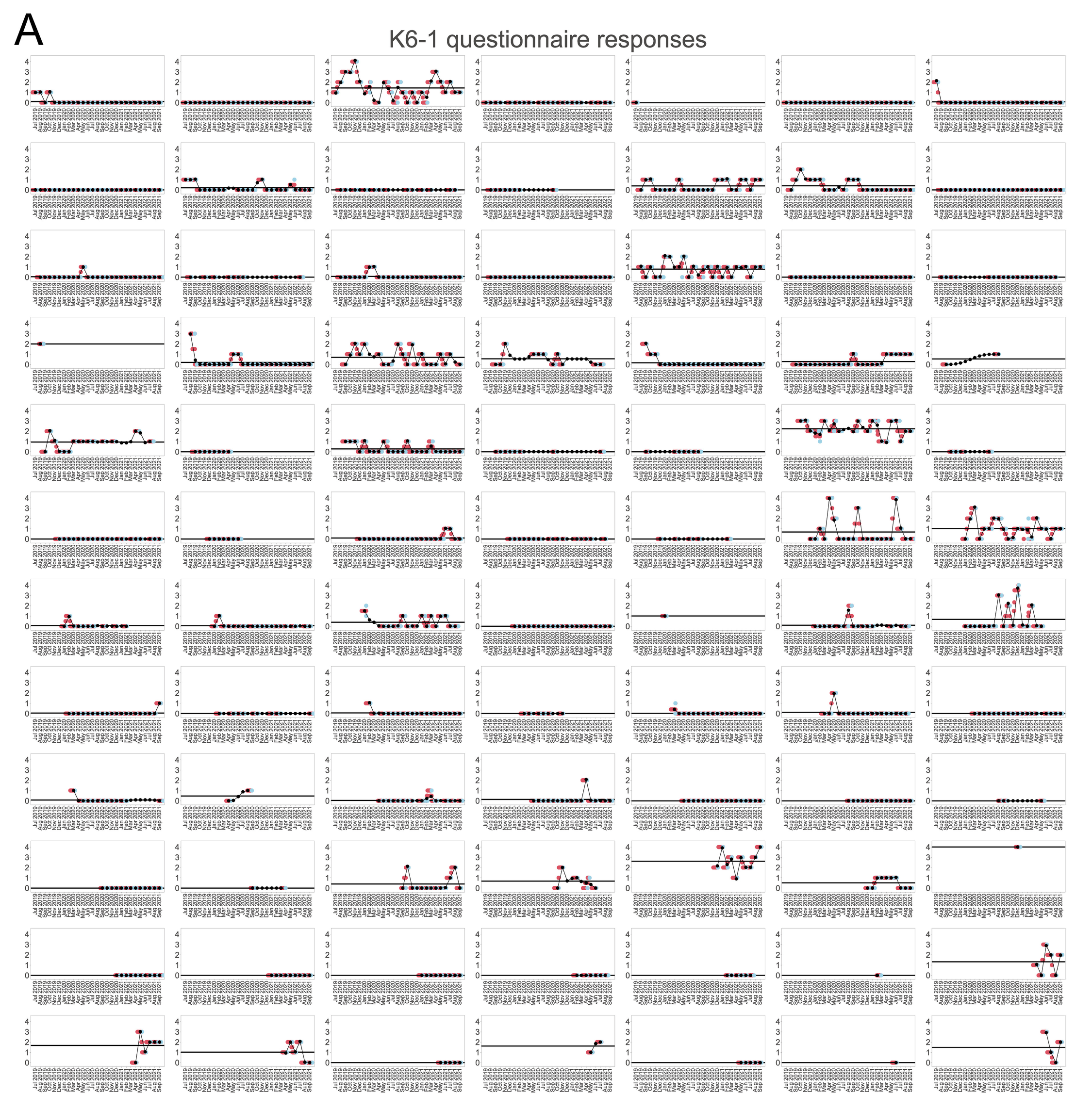


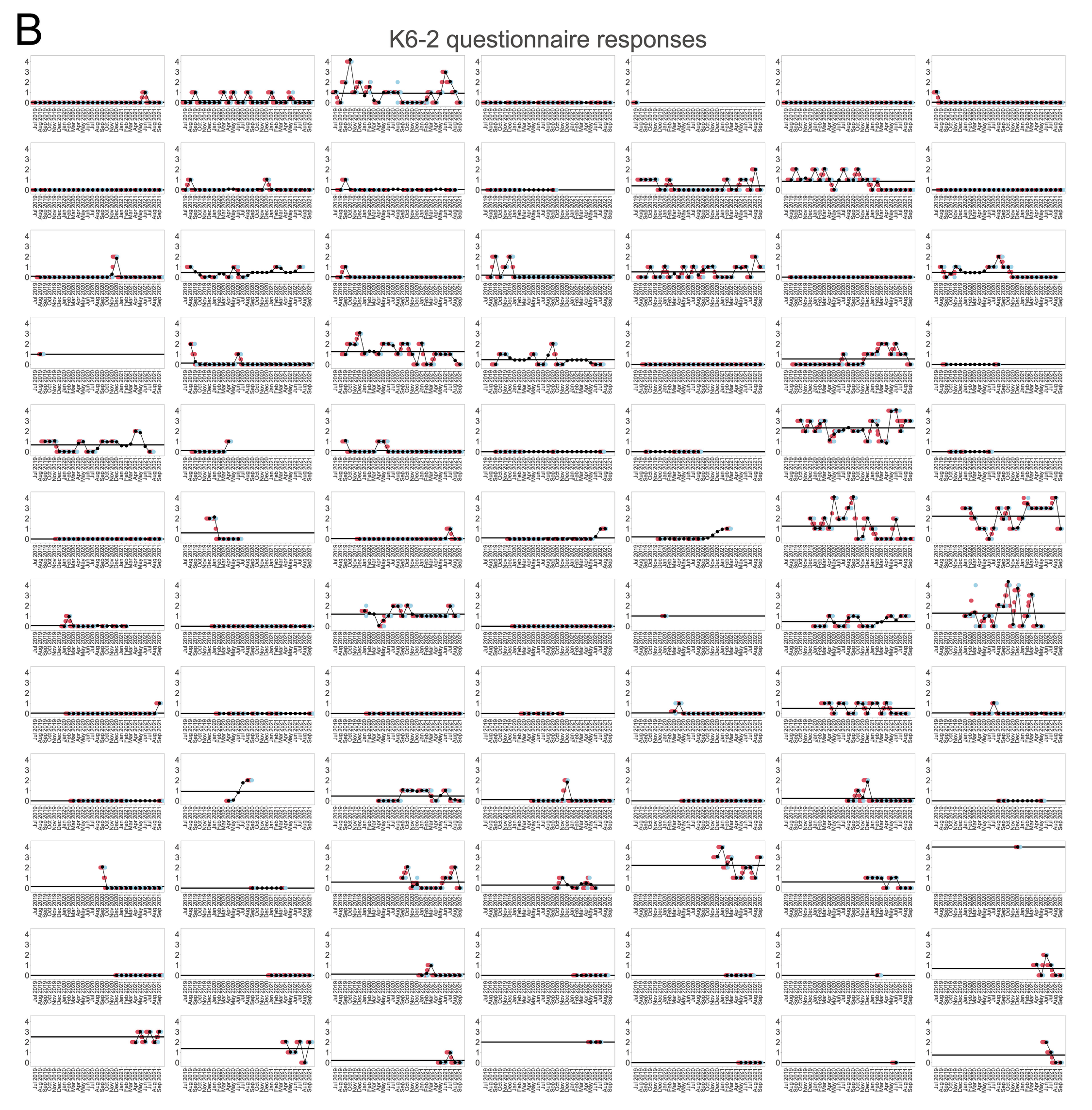


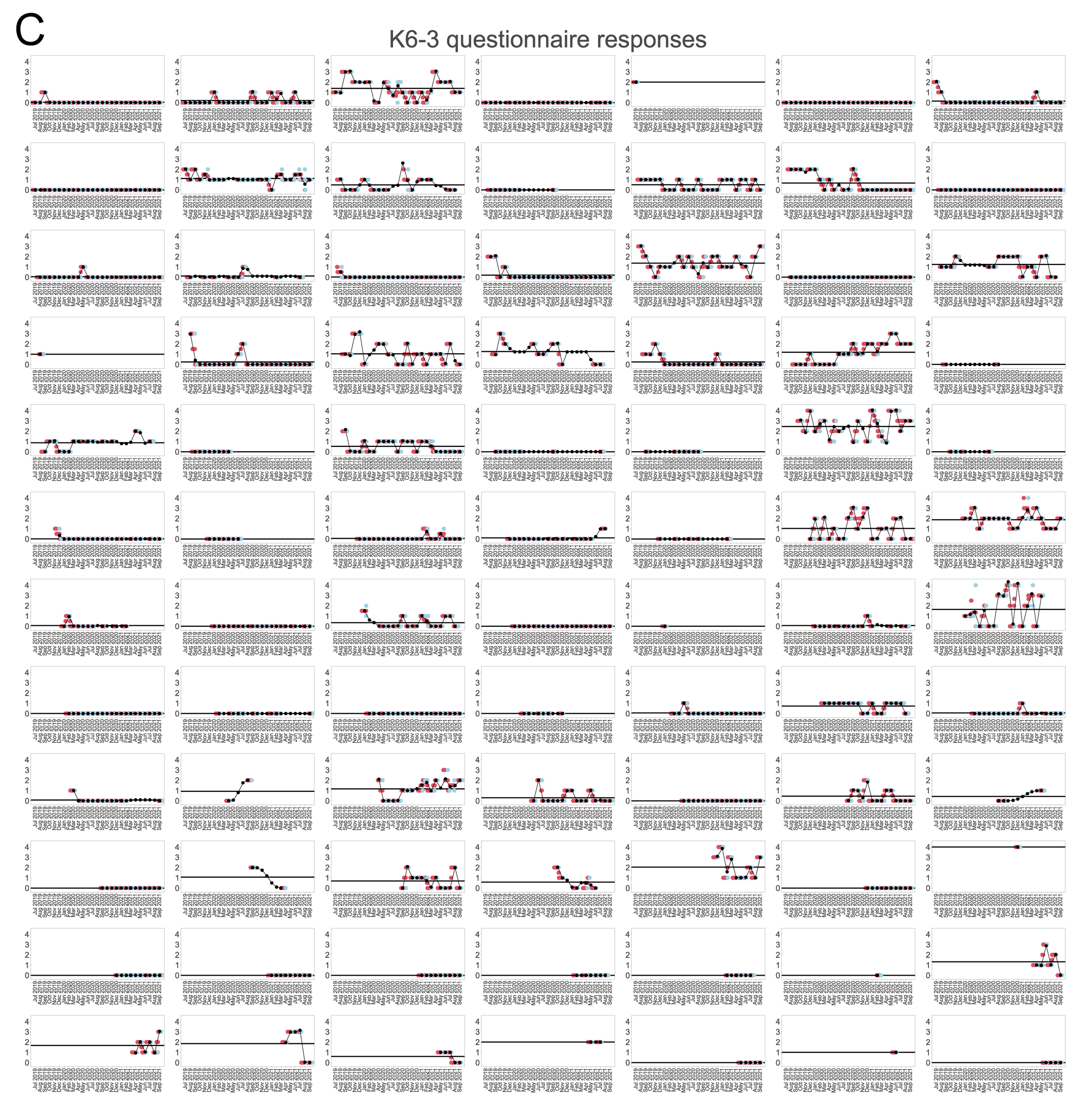


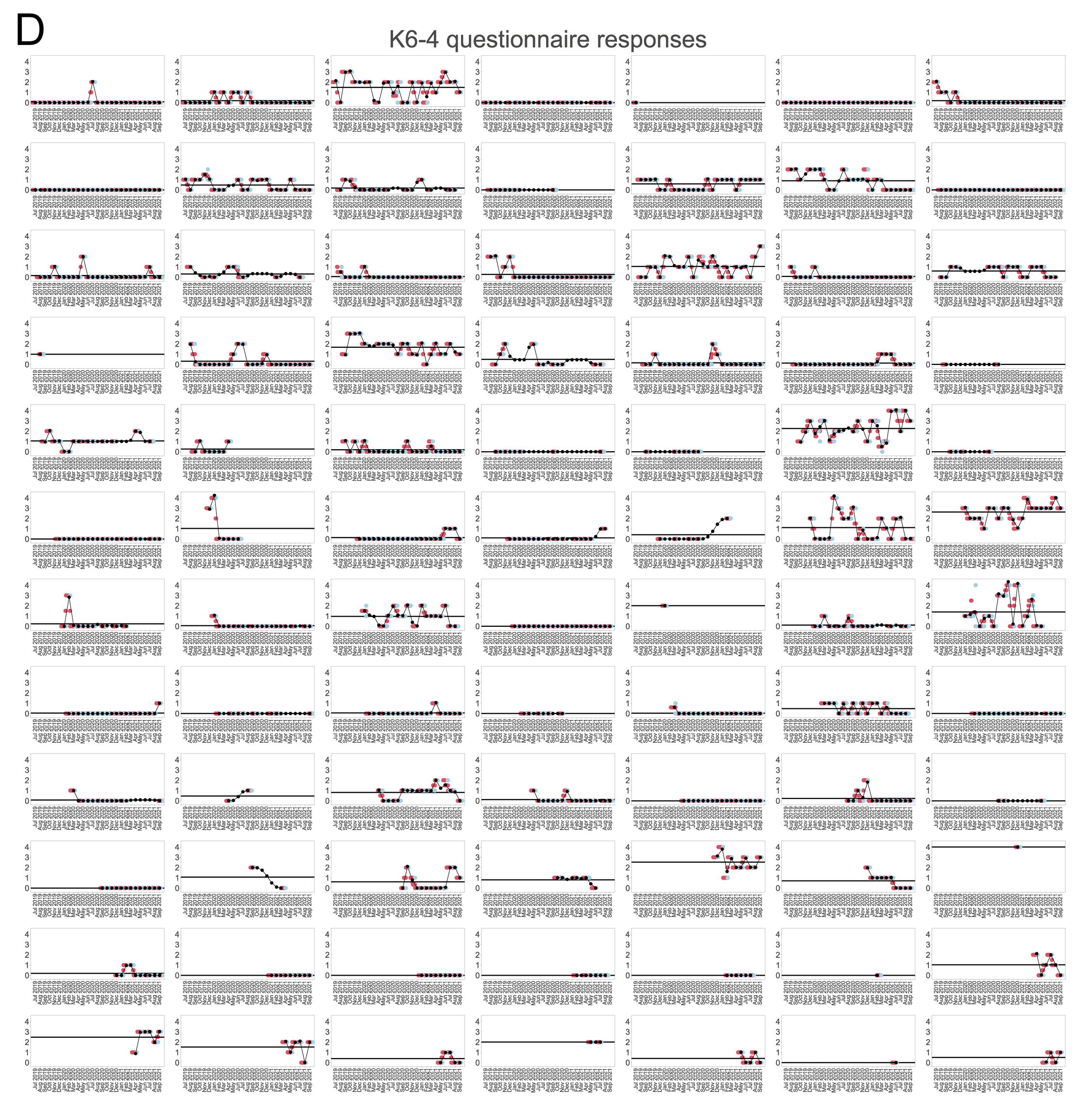


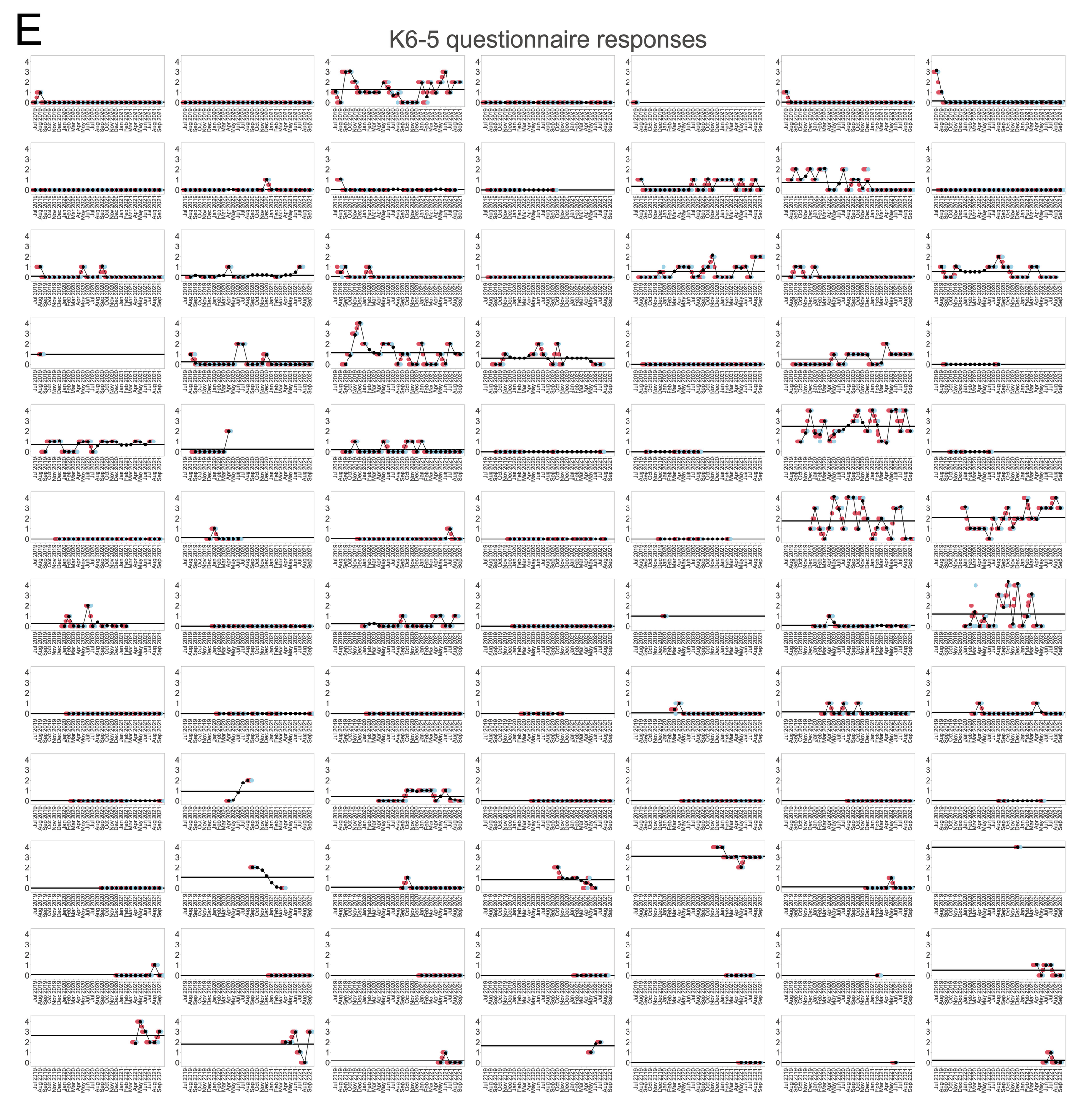


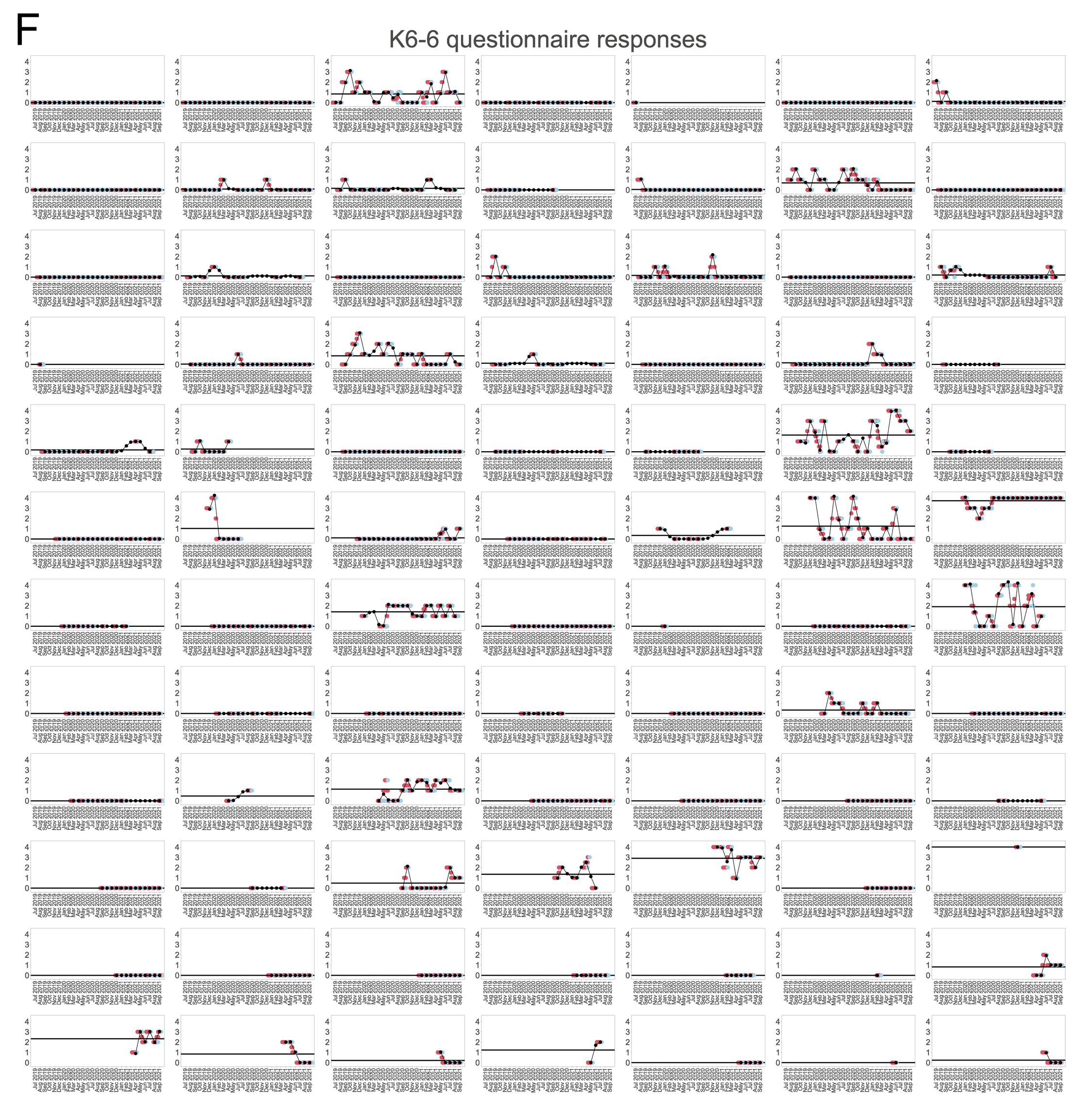


**S2 Fig |** **Preprocessing of each participant's questionnaire responses:** The process and results of the preprocessing of each participant's questionnaire responses are plotted (see **Methods**). Light blue dots are data collection points, red dots are the data points closest to the data collection point and extended to the previous 30 days, black dots are monthly data interpolated by Gaussian process regression, and the black horizontal line indicates the average over time. (**A**)-(**F**) correspond to K6-1 to K6-6 questionnaire responses. Panels at the same position in each of (**A**)-(**F**) are those of the same participant.
