# Supplementary material for: Psychological distress among Japanese high school students during the COVID-19 pandemic: An energy landscape analysis
Source: PLoS Med. 2026 Jan 22;23(1):e1004884. doi: 10.1371/journal.pmed.1004884 (PMC12826503; doi:10.1371/journal.pmed.1004884)
Supplement: S3 Fig — (DOCX) [file pmed.1004884.s003.docx]

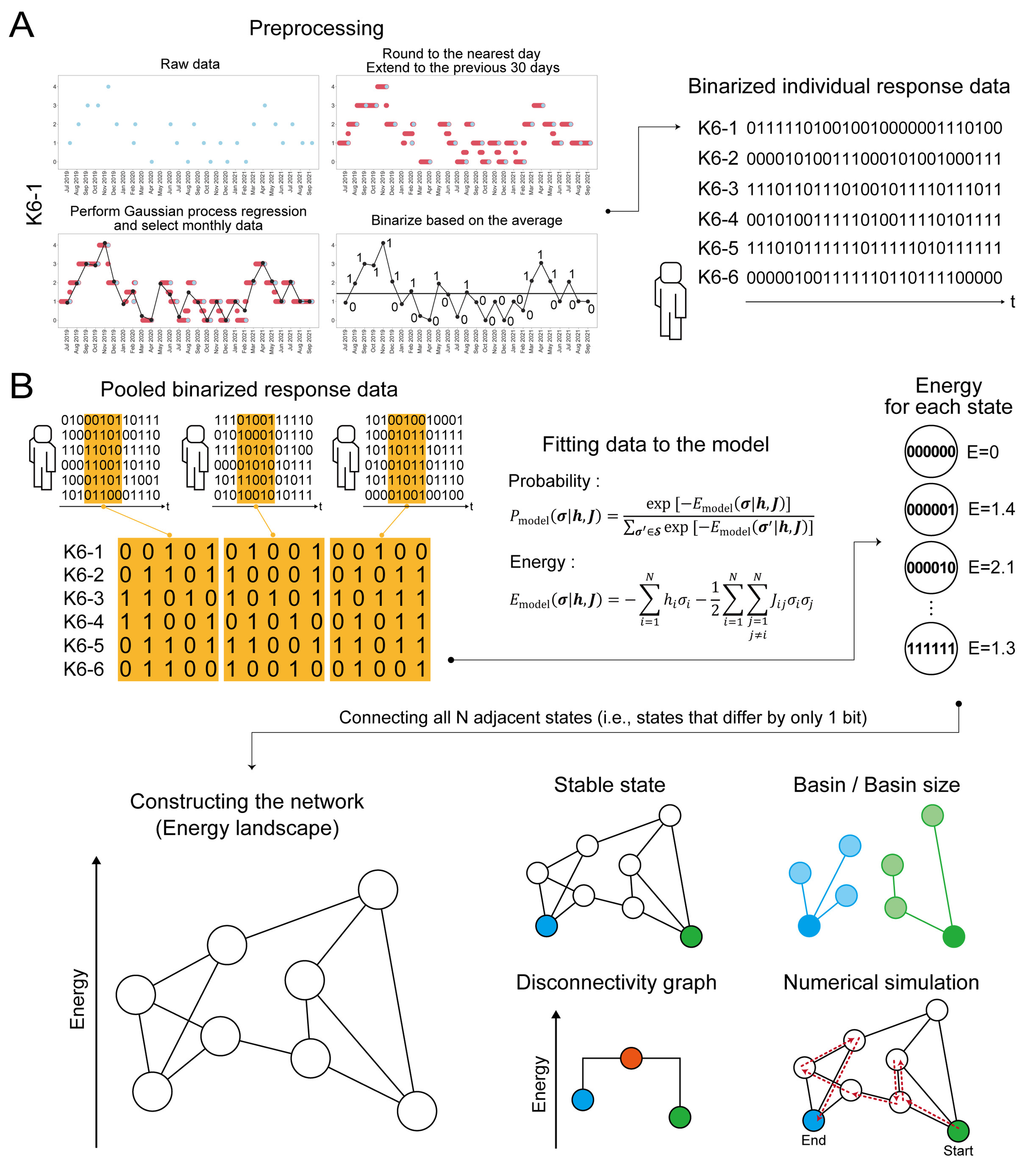


**S3 Fig | Schematic illustration of energy landscape analysis on the K6 questionnaire: (A)** Preprocessing and binarization of questionnaire response data. First, each data point was rounded to the nearest day and extended to the previous 30 days. Then, a Gaussian process regression was performed to smoothly connect the points. Finally, data on the first day of each month were selected and binarized based on the average (1 if above average, 0 otherwise). The resulting data consist of six-dimensional binary vectors (e.g., 001100), of which each entry corresponds to the response for an item on the K6 questionnaire. **(B)** Application of energy landscape analysis to pooled binarized questionnaire response data. The binarized response data from (**A**) were pooled over a given time period and individuals and fitted to the model. As a result, each state (six-dimensional binary vector) was assigned an energy (real value related to probability). The network was then constructed by connecting all adjacent states (i.e., states that differ by only 1 bit). From the constructed network, stable states (states with lower energy than all adjacent states) are determined and shown as blue and green states. The network is divided into separate basins (blue basins and green basins). The basin size refers to the number of states belonging to a basin. The disconnectivity graph shows the hierarchical relationship between stable states and intermediate states shown in orange. Numerical simulations allow us to simulate the time evolution of states based on the obtained energy landscape. The red dotted line shows an example time evolution. Additional details are provided in **S1 Note** (Glossary) and **Methods**.
