# Supplementary material for: Psychological distress among Japanese high school students during the COVID-19 pandemic: An energy landscape analysis
Source: PLoS Med. 2026 Jan 22;23(1):e1004884. doi: 10.1371/journal.pmed.1004884 (PMC12826503; doi:10.1371/journal.pmed.1004884)
Supplement: S4 Fig — (DOCX) [file pmed.1004884.s004.docx]

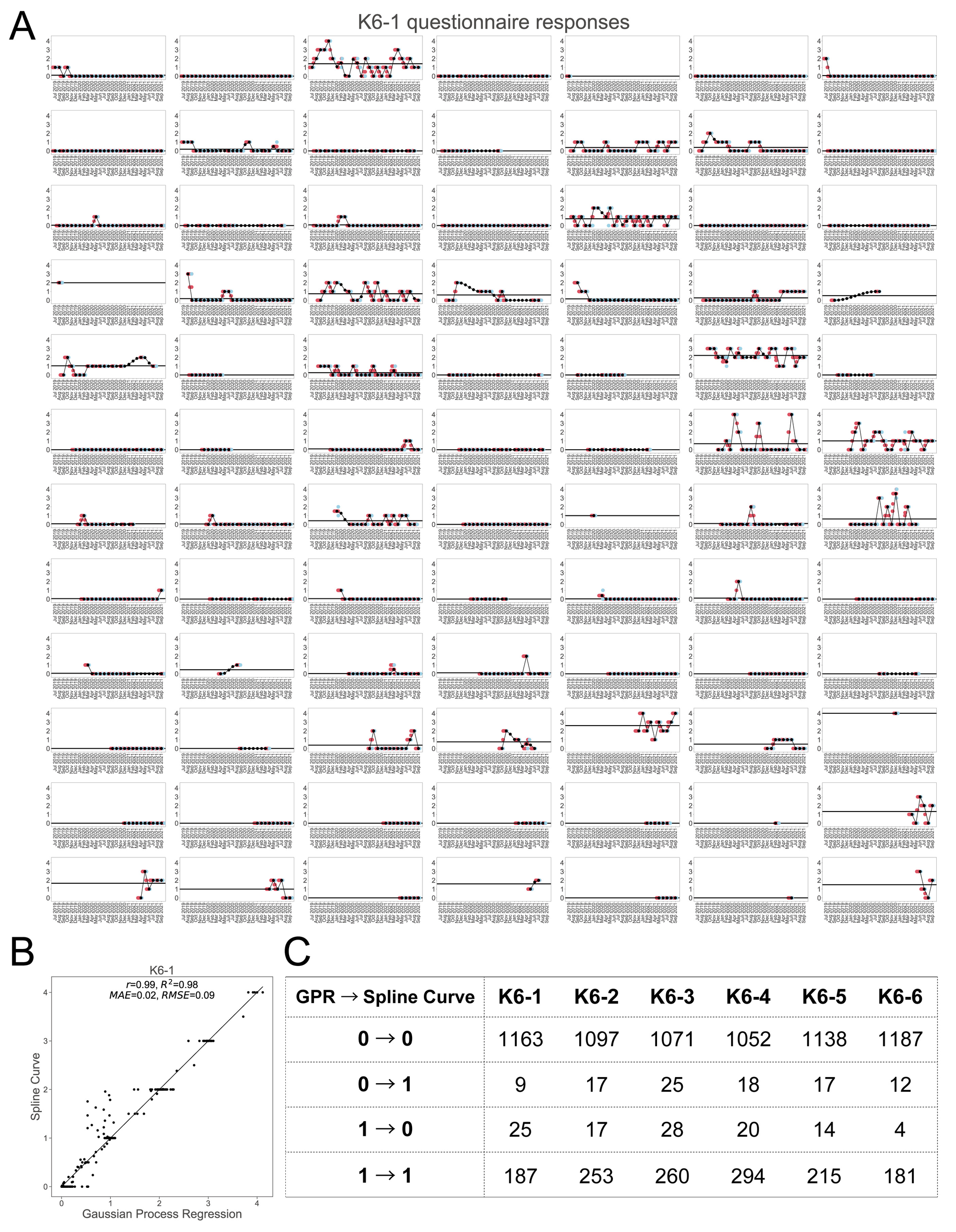


**S4 Fig | Different smoothing methods: (A)** Smoothing by spline curves (for K6-1 questionnaire responses). The process and result of the preprocessing of each participant's questionnaire responses are plotted (see **Methods**). Light blue dots are data collection points, red dots are the data points closest to the data collection point and extended to the previous 30 days, black dots are monthly data interpolated by spline curves, and the black horizontal line indicates the average over time. **(B)** Comparison of values obtained by Gaussian process regression (**S2A Fig**) and by spline curves (for K6-1 questionnaire responses). **(C)** Comparison of binarization results obtained by Gaussian process regression and by spline curves.
