# Supplementary material for: Psychological distress among Japanese high school students during the COVID-19 pandemic: An energy landscape analysis
Source: PLoS Med. 2026 Jan 22;23(1):e1004884. doi: 10.1371/journal.pmed.1004884 (PMC12826503; doi:10.1371/journal.pmed.1004884)
Supplement: S5 Fig — (DOCX) [file pmed.1004884.s005.docx]

**
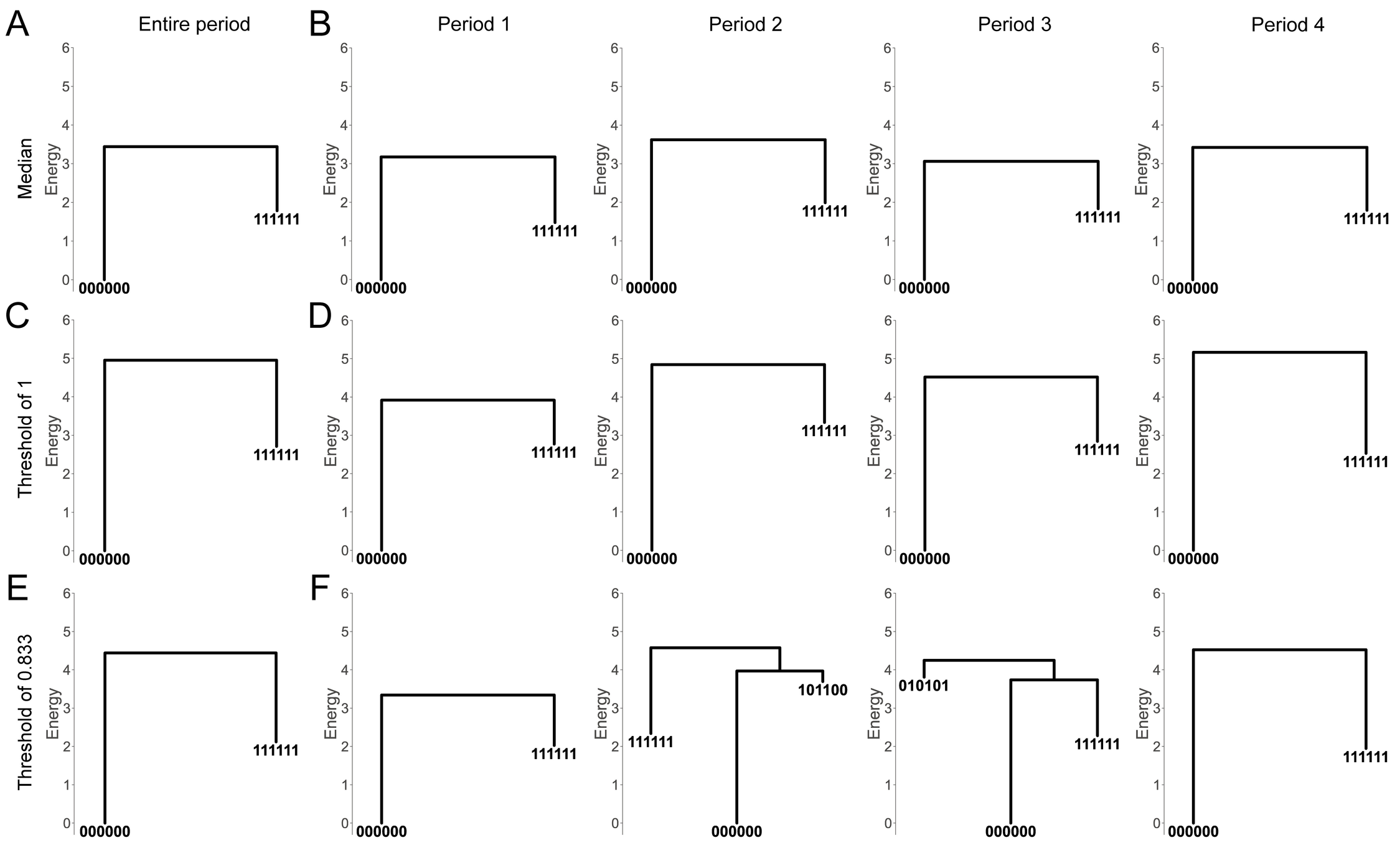
**

**S5 Fig | Different binarization methods:** Disconnectivity graphs for the four periods are shown when the K6 score for each item was binarized by: **(A)(B)** the individual median, **(C)(D)** the threshold of 1, or **(E)(F)** the threshold of 0.833 (=5/6). **(A)(C)(E)** are for the entire period, and **(B)(D)(F)** are for Periods 1-4.
