# Supplementary material for: Psychological distress among Japanese high school students during the COVID-19 pandemic: An energy landscape analysis
Source: PLoS Med. 2026 Jan 22;23(1):e1004884. doi: 10.1371/journal.pmed.1004884 (PMC12826503; doi:10.1371/journal.pmed.1004884)
Supplement: S6 Fig — (DOCX) [file pmed.1004884.s006.docx]

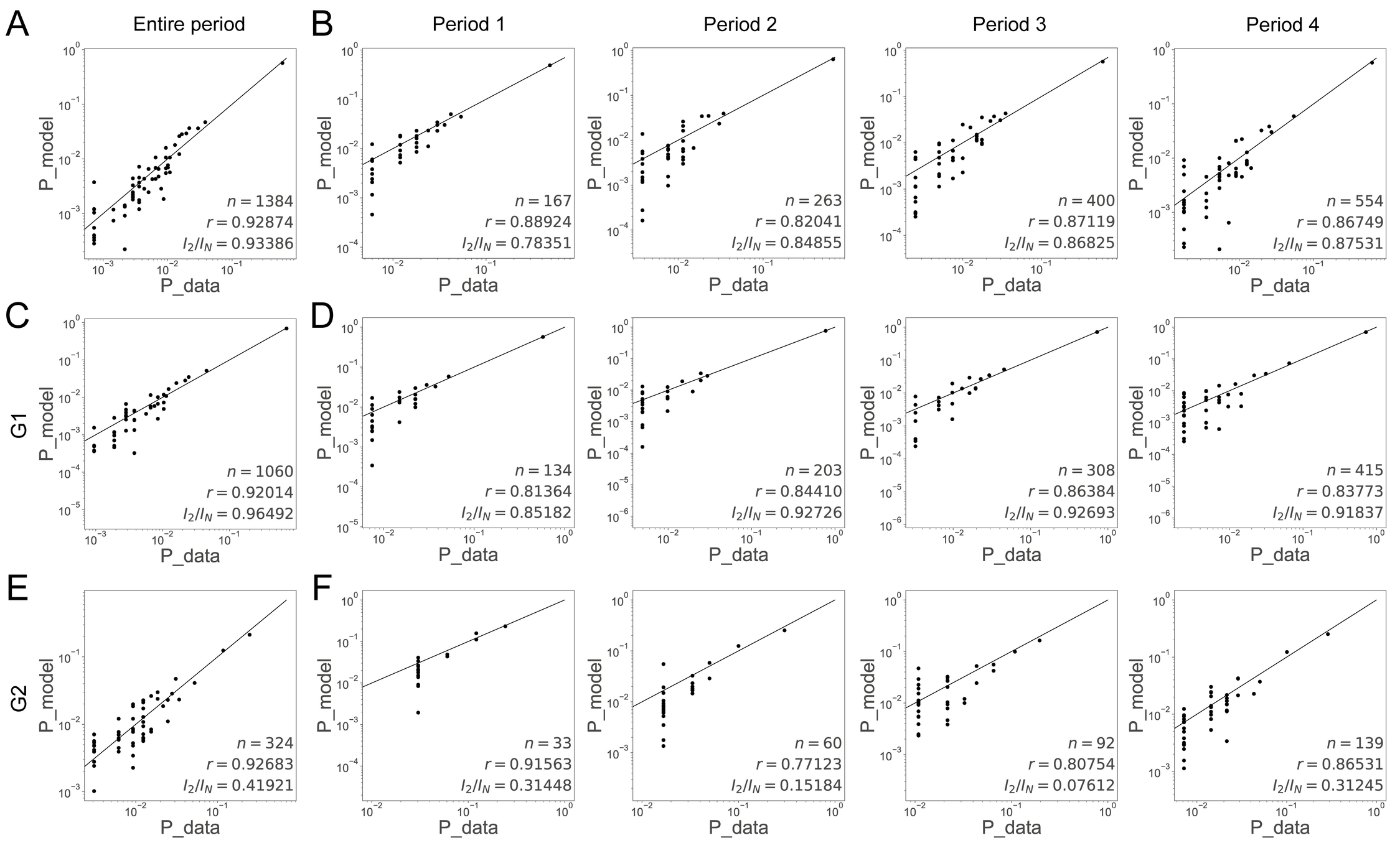


**S6 Fig |** **Accuracy of the model fitting:** The accuracy of data fitting to the model is shown. For all possible states ($2^{6}=64$ states), the probabilities calculated from the data $P_{\text{data}}$ and from the model $P_{\text{model}}$ are plotted as black dots. If $P_{\text{data}}$ equals $P_{\text{model}}$ for a state, the state is plotted on the black line $y=x$. $n$ is the number of data points used for the estimation. $r$ and $I_{2}/I_{N}$ are calculated as accuracy indices (see **Methods**). (**A**)(**B**) all participants, (**C**)(**D**) G1 participants, (**E**)(**F**) G2 participants. The leftmost column is for the entire period, and the remaining columns are for Periods 1-4.
