# Supplementary material for: Psychological distress among Japanese high school students during the COVID-19 pandemic: An energy landscape analysis
Source: PLoS Med. 2026 Jan 22;23(1):e1004884. doi: 10.1371/journal.pmed.1004884 (PMC12826503; doi:10.1371/journal.pmed.1004884)
Supplement: S7 Fig — (DOCX) [file pmed.1004884.s007.docx]

**
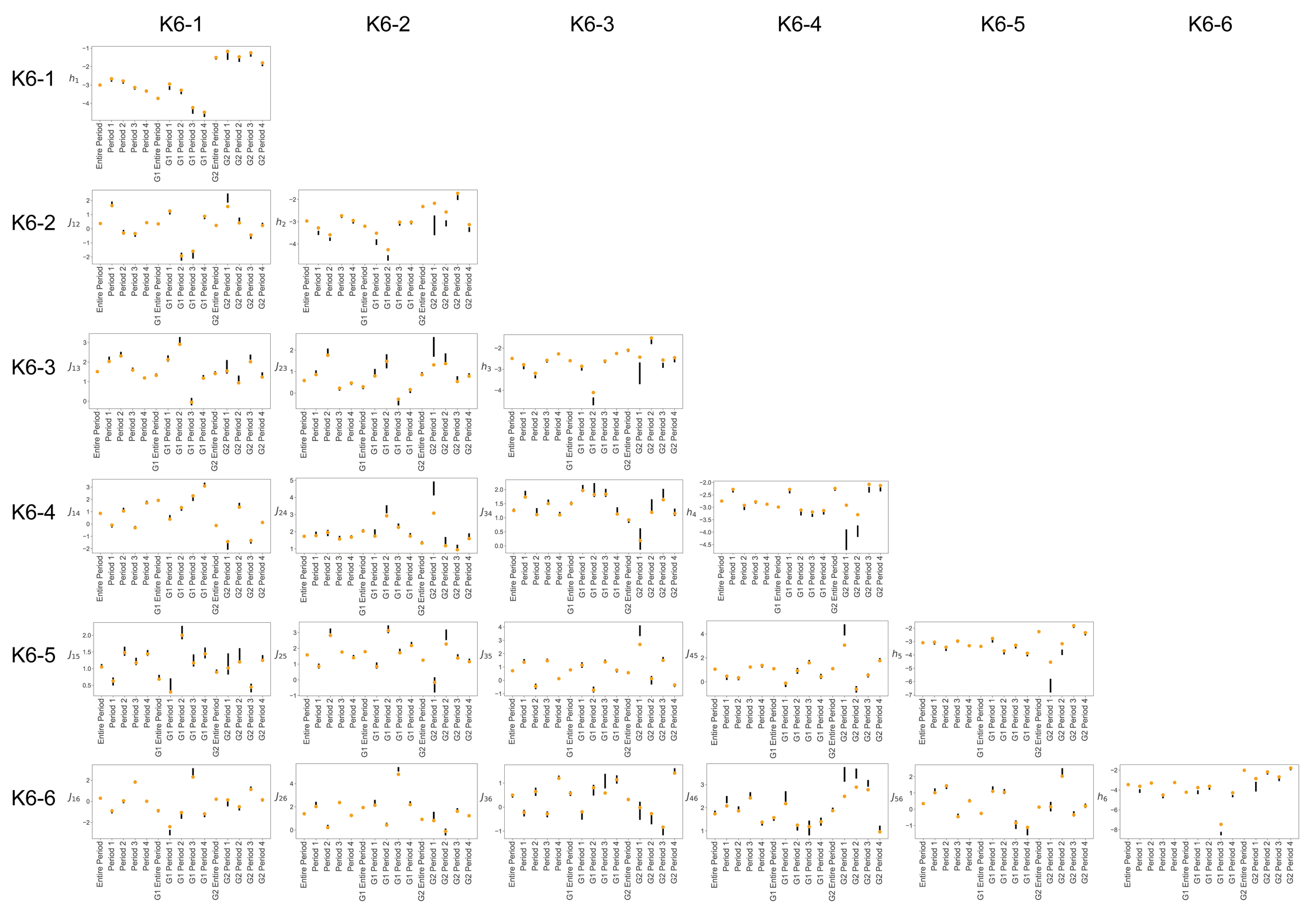
**

**S7 Fig | Confidence intervals of parameter estimate:** The 95% confidence intervals of the estimated parameters, $h_{i}$ and $J_{ij}$ are displayed for all participants (entire period and periods 1-4), for G1 (entire period and periods 1-4), and for G2 (entire period and periods 1-4). The diagonal panels show the 95% confidence interval of $h_{i}$ parameters. The panels, except those on the diagonal, show the 95% confidence interval of $J_{ij}$ parameters. The estimation was performed 100 times using bootstrapped data. The permissible error $\varepsilon$ was set to $0.005$ for all conditions; the learning rate $\alpha$ was set to $0.001$ for all conditions.
