# Supplementary material for: Psychological distress among Japanese high school students during the COVID-19 pandemic: An energy landscape analysis
Source: PLoS Med. 2026 Jan 22;23(1):e1004884. doi: 10.1371/journal.pmed.1004884 (PMC12826503; doi:10.1371/journal.pmed.1004884)
Supplement: S8 Fig — (DOCX) [file pmed.1004884.s008.docx]

**
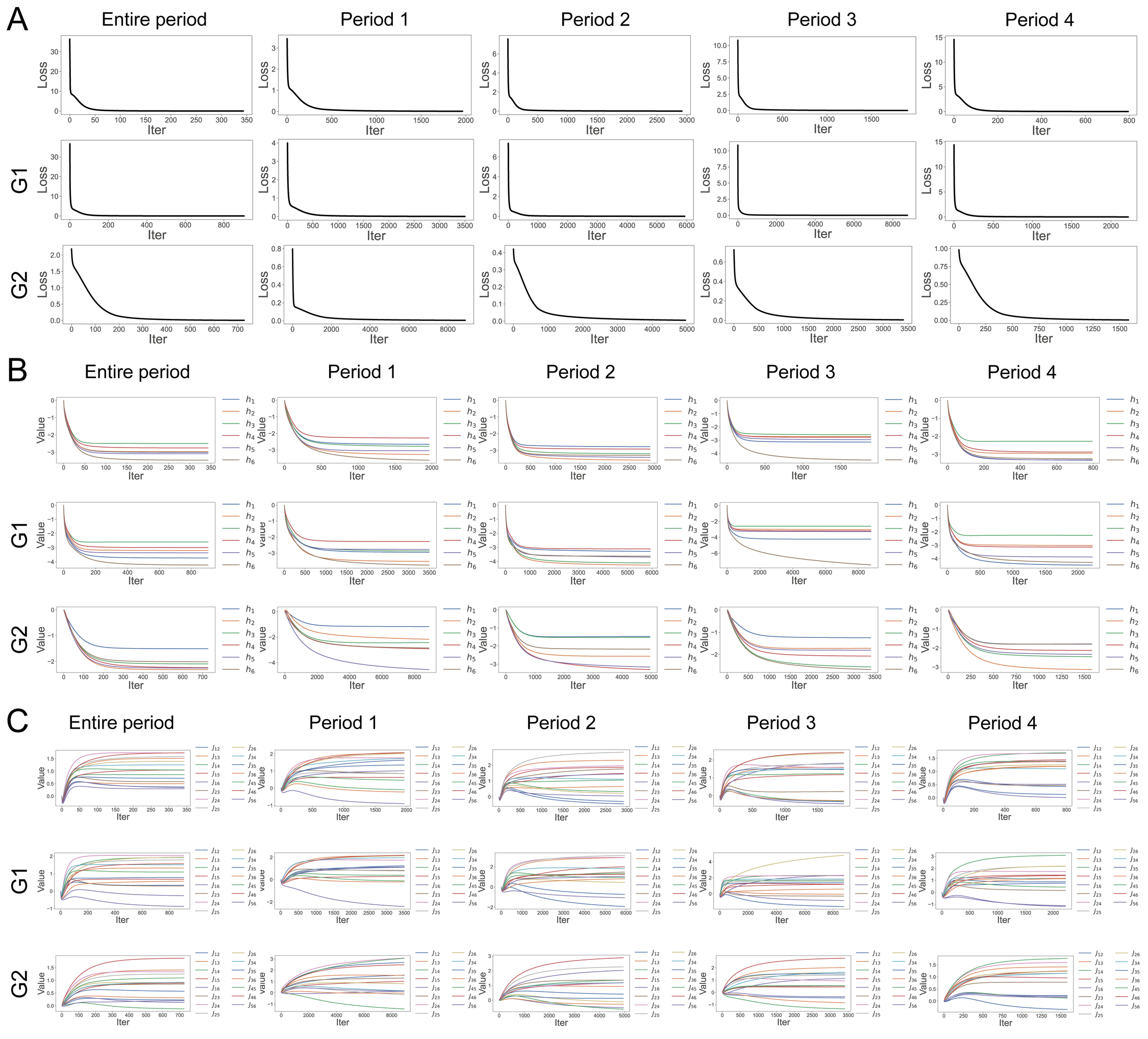
**

**S8 Fig | Convergence of estimation errors and changes in parameter estimates across iterations: (A)** The convergences of estimation errors across iterations are displayed for all participants (entire period and periods 1-4), for G1 (entire period and periods 1-4), and for G2 (entire period and periods 1-4). **(B)(C)** The change in $h_{i}$ and $J_{ij}$ parameter estimates across iterations is displayed for all participants (entire period and periods 1-4), for G1 (entire period and periods 1-4), and for G2 (entire period and periods 1-4).
