# Supplementary material for: Psychological distress among Japanese high school students during the COVID-19 pandemic: An energy landscape analysis
Source: PLoS Med. 2026 Jan 22;23(1):e1004884. doi: 10.1371/journal.pmed.1004884 (PMC12826503; doi:10.1371/journal.pmed.1004884)
Supplement: S9 Fig — (DOCX) [file pmed.1004884.s009.docx]

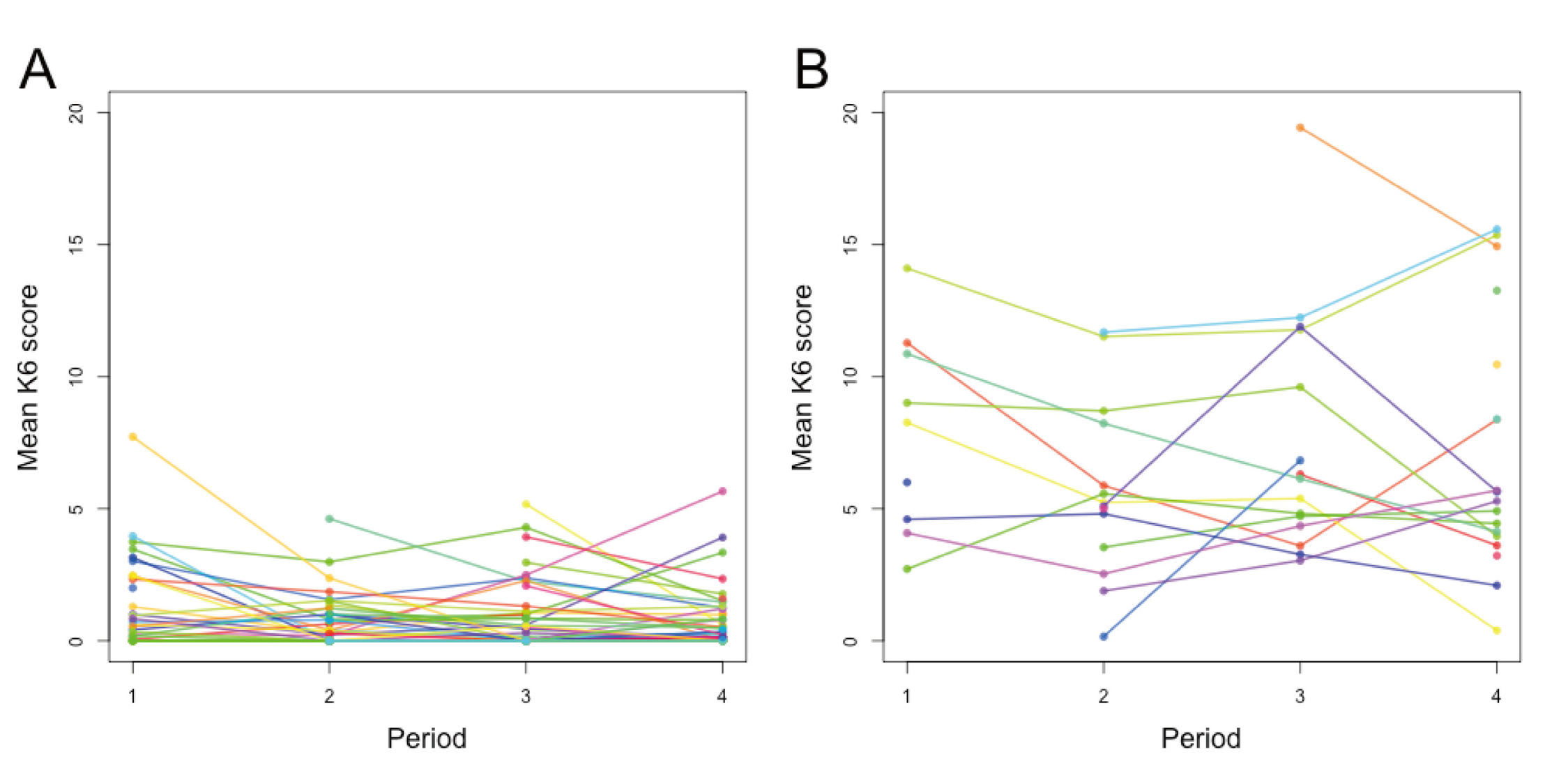


**S9 Fig | Changes in total K6 scores across different periods:** The mean of the total K6 score in each period is plotted for **(A)** G1 participants (n = 63) and **(B)** G2 participants (n = 21). The same individuals are connected by lines of the same color.
