# Supplementary material for: Psychological distress among Japanese high school students during the COVID-19 pandemic: An energy landscape analysis
Source: PLoS Med. 2026 Jan 22;23(1):e1004884. doi: 10.1371/journal.pmed.1004884 (PMC12826503; doi:10.1371/journal.pmed.1004884)
Supplement: S10 Fig — (DOCX) [file pmed.1004884.s010.docx]

**
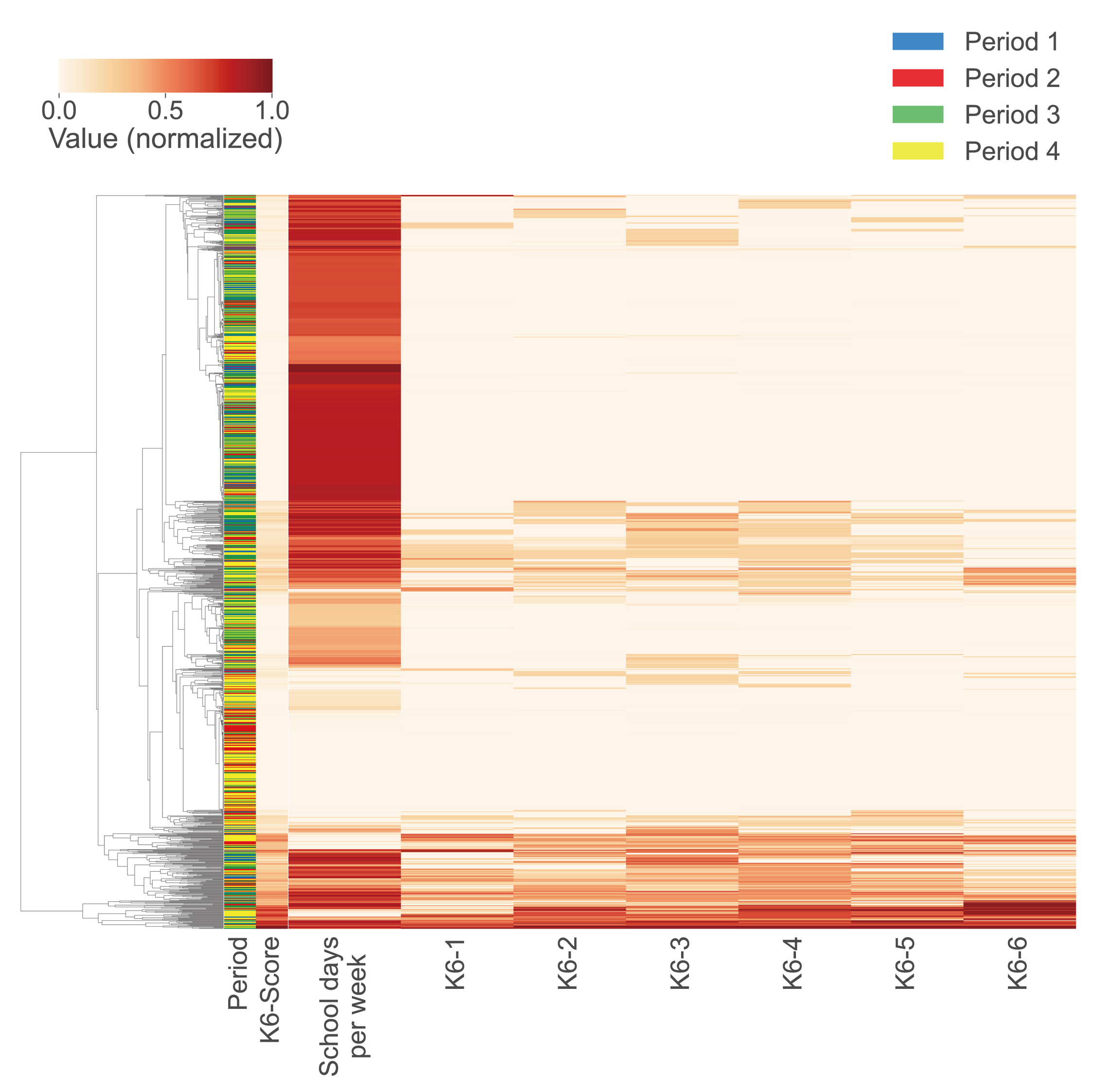
**

**S10 Fig | Relationship between school days per week and K6 questionnaire responses:** Hierarchical clustering of pooled questionnaire responses from all participants revealed a correlation between fewer school days per week and lower K6 questionnaire responses (depicted in the lighter area near the bottom). Period refers to the time period in which the data were collected and is displayed in one of four colors (blue, red, green, and yellow for Periods 1, 2, 3, and 4, respectively).
