# Supplementary material for: Psychological distress among Japanese high school students during the COVID-19 pandemic: An energy landscape analysis
Source: PLoS Med. 2026 Jan 22;23(1):e1004884. doi: 10.1371/journal.pmed.1004884 (PMC12826503; doi:10.1371/journal.pmed.1004884)
Supplement: S11 Fig — (DOCX) [file pmed.1004884.s011.docx]

**
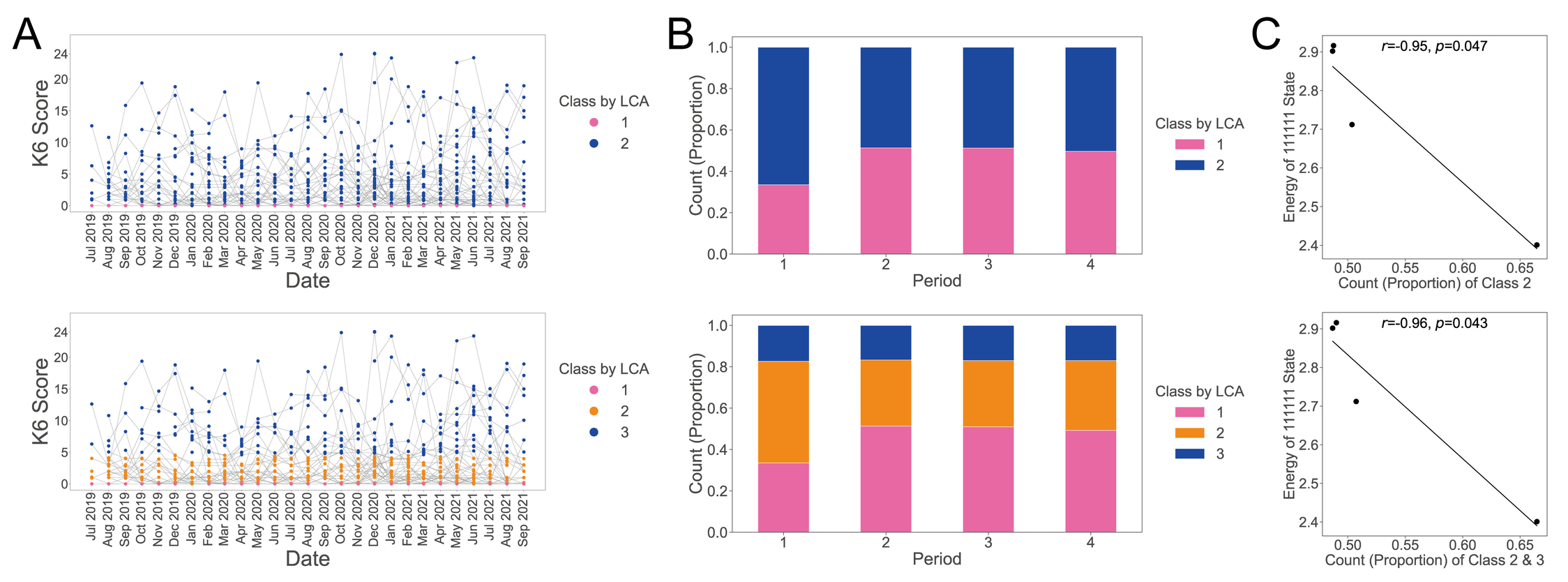
**

**S11 Fig |** **Latent class analysis of time-series K6 questionnaire responses for all participants: (A)** Participant-level plots showing classes of datapoints based on latent class analysis with 2- and 3-class solutions. **(B)** Count (proportion) of datapoints in each class identified by latent class analysis across the 4 periods for 2- and 3-class solutions. **(C)** Relationship between the energy of 111111 state estimated by energy landscape analysis and the count (proportion) of data points in each class identified by latent class analysis. Each point represents one of the 4 periods. 111111 (binarized responses of 1 to all six items) represents the “depressive” state. These were negatively correlated (*r* = -0.95, *p* = 0.047; *r* = -0.96, *p* = 0.043).
