# Supplementary material for: Psychological distress among Japanese high school students during the COVID-19 pandemic: An energy landscape analysis
Source: PLoS Med. 2026 Jan 22;23(1):e1004884. doi: 10.1371/journal.pmed.1004884 (PMC12826503; doi:10.1371/journal.pmed.1004884)
Supplement: S12 Fig — (DOCX) [file pmed.1004884.s012.docx]

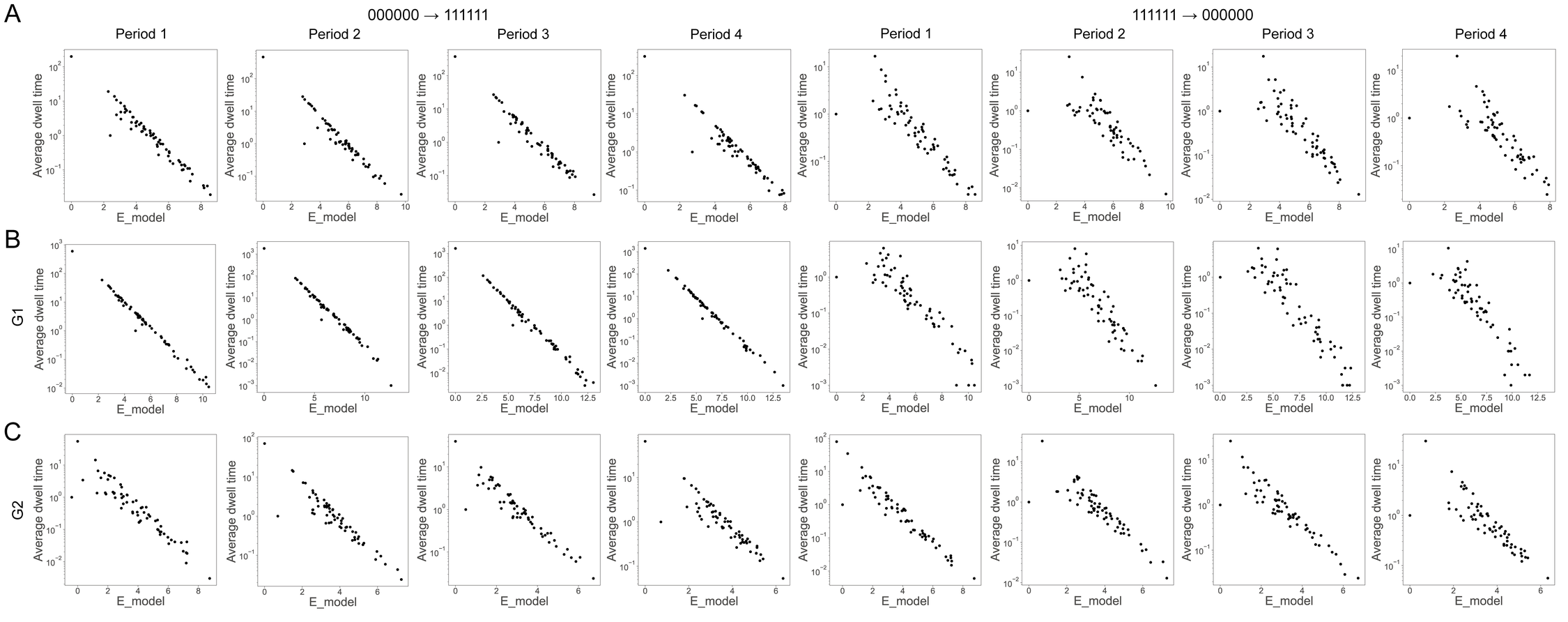


**S12 Fig | The average dwell time of each state in the simulation:** The logarithm of the average dwell time of each state in the simulation is plotted with its energy. The former is a linear function of the latter with a negative slope, consistent with the Boltzmann distribution. **(A)** All participants, **(B)** G1 participants, **(C)** G2 participants.
