# Supplementary material for: Psychological distress among Japanese high school students during the COVID-19 pandemic: An energy landscape analysis
Source: PLoS Med. 2026 Jan 22;23(1):e1004884. doi: 10.1371/journal.pmed.1004884 (PMC12826503; doi:10.1371/journal.pmed.1004884)
Supplement: S13 Fig — (DOCX) [file pmed.1004884.s013.docx]

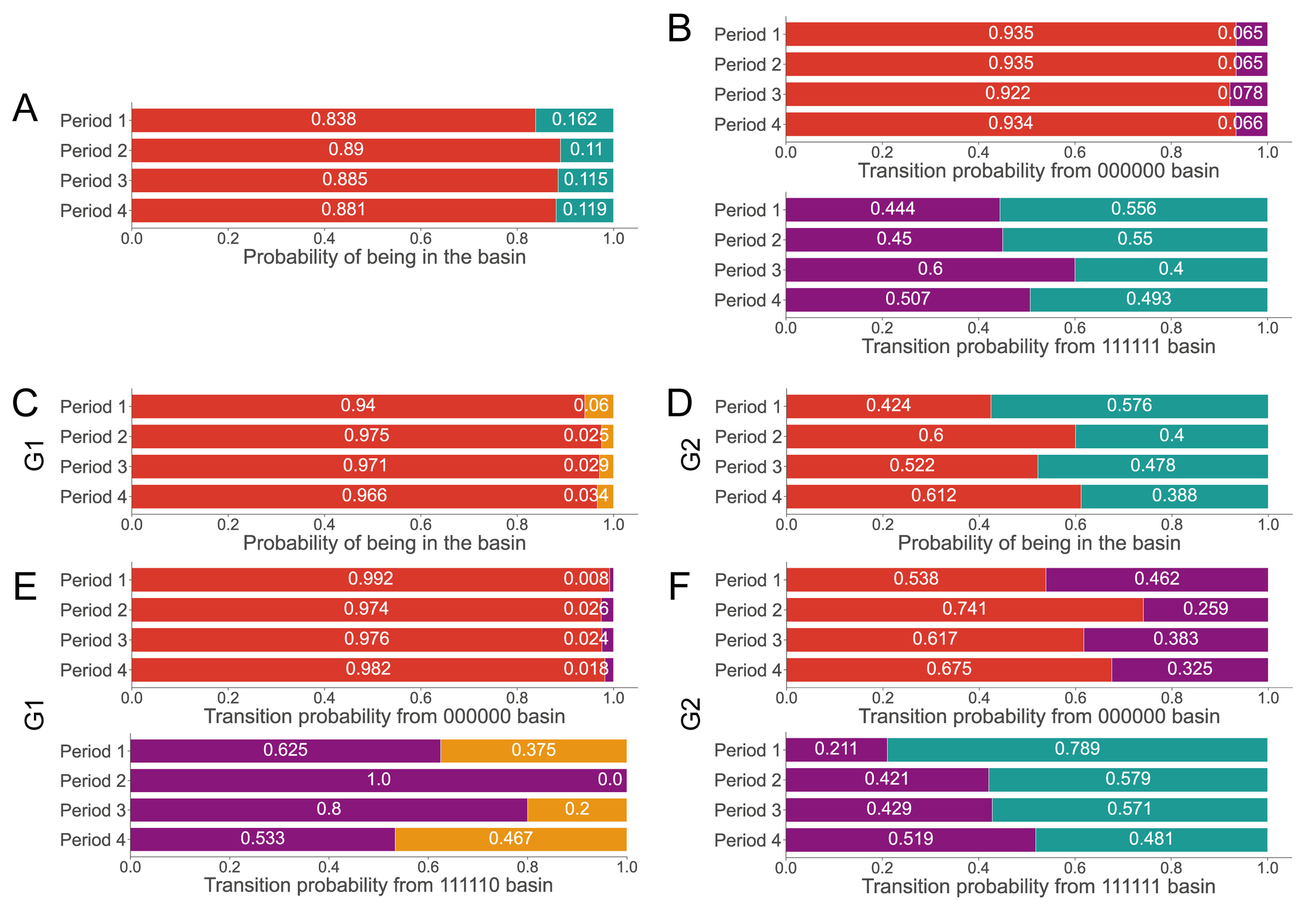


**S13 Fig | Probabilities of staying in the basin and of transitioning between basins: (A)** The probabilities of being in each basin for the 4 periods. The basin 000000 is shown in orange; the basin 111111 is shown in green. **(B)** Transition probabilities between the basins for the 4 periods. Top: Transition probabilities from the 000000 basin to itself are shown in orange; those from the 000000 basin to the 111111 basin (i.e., probabilities of switching basins) are shown in purple. Bottom: Transition probabilities from the 111111 basin to itself are shown in green; those from the 111111 basin to the 000000 basin (i.e., probabilities of switching basins) are shown in purple. **(C)(D)** The probabilities of being in each basin for G1 and G2 participants is plotted for the 4 periods. The 000000 basin is shown in orange; the 111110 basin is shown in yellow; the 111111 basin is shown in green. **(E)(F)** Transition probabilities between the basins for G1 and G2 participants are plotted for the 4 periods. Top: Transition probabilities from the 000000 basin to itself are shown in orange; those from the 000000 basin to the 111111 basin are shown in purple. Bottom: Transition probabilities from the 111110 (or 111111) basin to itself are shown in yellow (or green, respectively); those from the 111110 (or 111111) basin to the 000000 basin are shown in purple.
