# Supplementary material for: Psychological distress among Japanese high school students during the COVID-19 pandemic: An energy landscape analysis
Source: PLoS Med. 2026 Jan 22;23(1):e1004884. doi: 10.1371/journal.pmed.1004884 (PMC12826503; doi:10.1371/journal.pmed.1004884)
Supplement: S14 Fig — (DOCX) [file pmed.1004884.s014.docx]

**
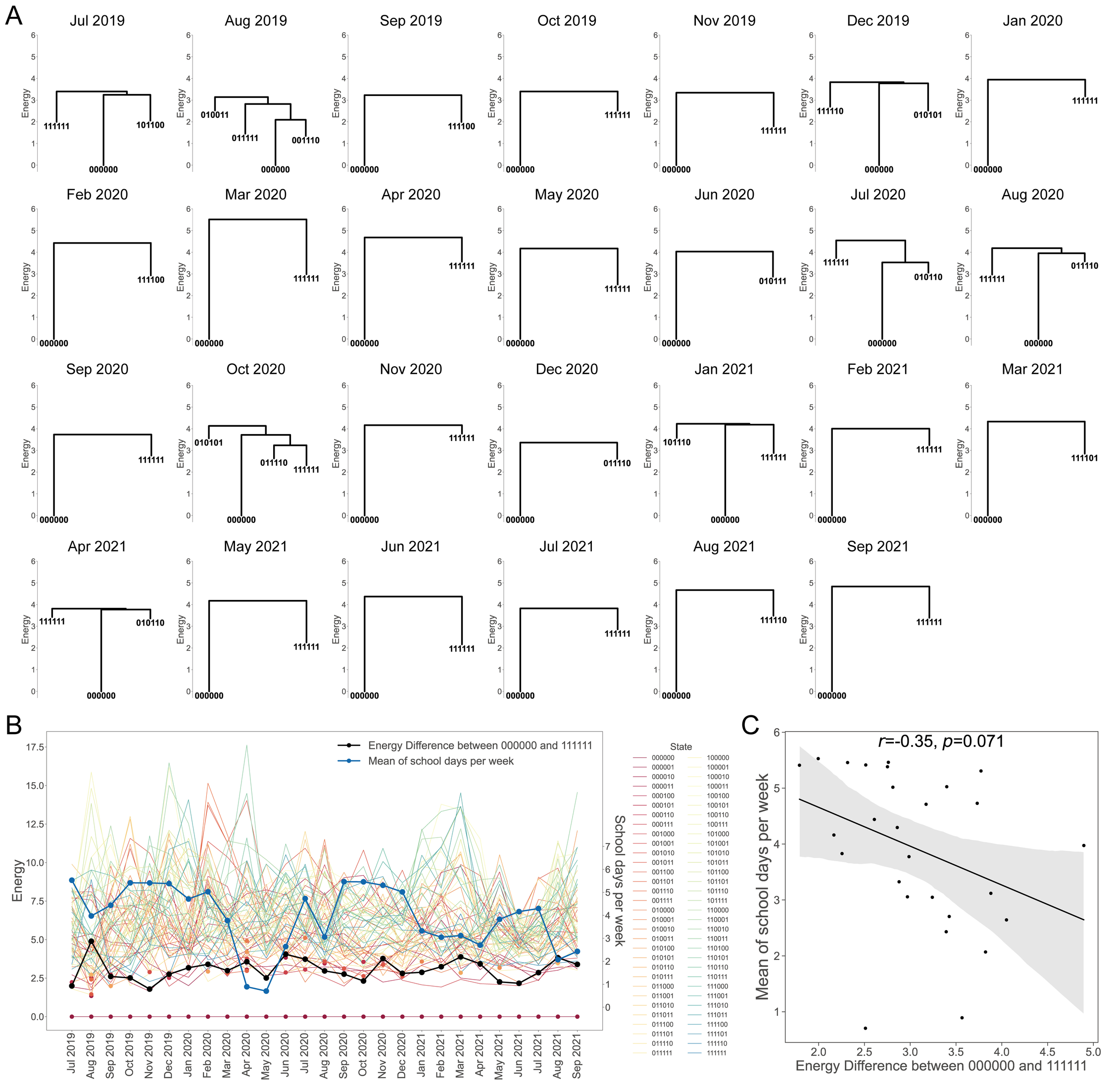
**

**S14 Fig | Energy landscape analysis of time-series K6 questionnaire responses by month: (A)** Disconnectivity graphs of the energy landscape are shown for the pooled data for each month. **(B)** The monthly energy transition of each state is plotted and overlaid. Stable states are indicated by solid circles. The thick black line shows the energy difference between 000000 (healthy state) and 111111 (depressive state). The thick blue line shows the number of school days per week. **(C)** Relationship between school days per week and Energy Difference between 000000 and 111111. The black line represents the trend line, and the gray shaded area indicates the 95% confidence interval (95% CI). The energy difference between 000000 and 111111 was negatively correlated with the number of school days per week (*r* = -0.35, *p* = 0.071).
