# Supplementary material for: Psychological distress among Japanese high school students during the COVID-19 pandemic: An energy landscape analysis
Source: PLoS Med. 2026 Jan 22;23(1):e1004884. doi: 10.1371/journal.pmed.1004884 (PMC12826503; doi:10.1371/journal.pmed.1004884)
Supplement: S15 Fig — (DOCX) [file pmed.1004884.s015.docx]

**
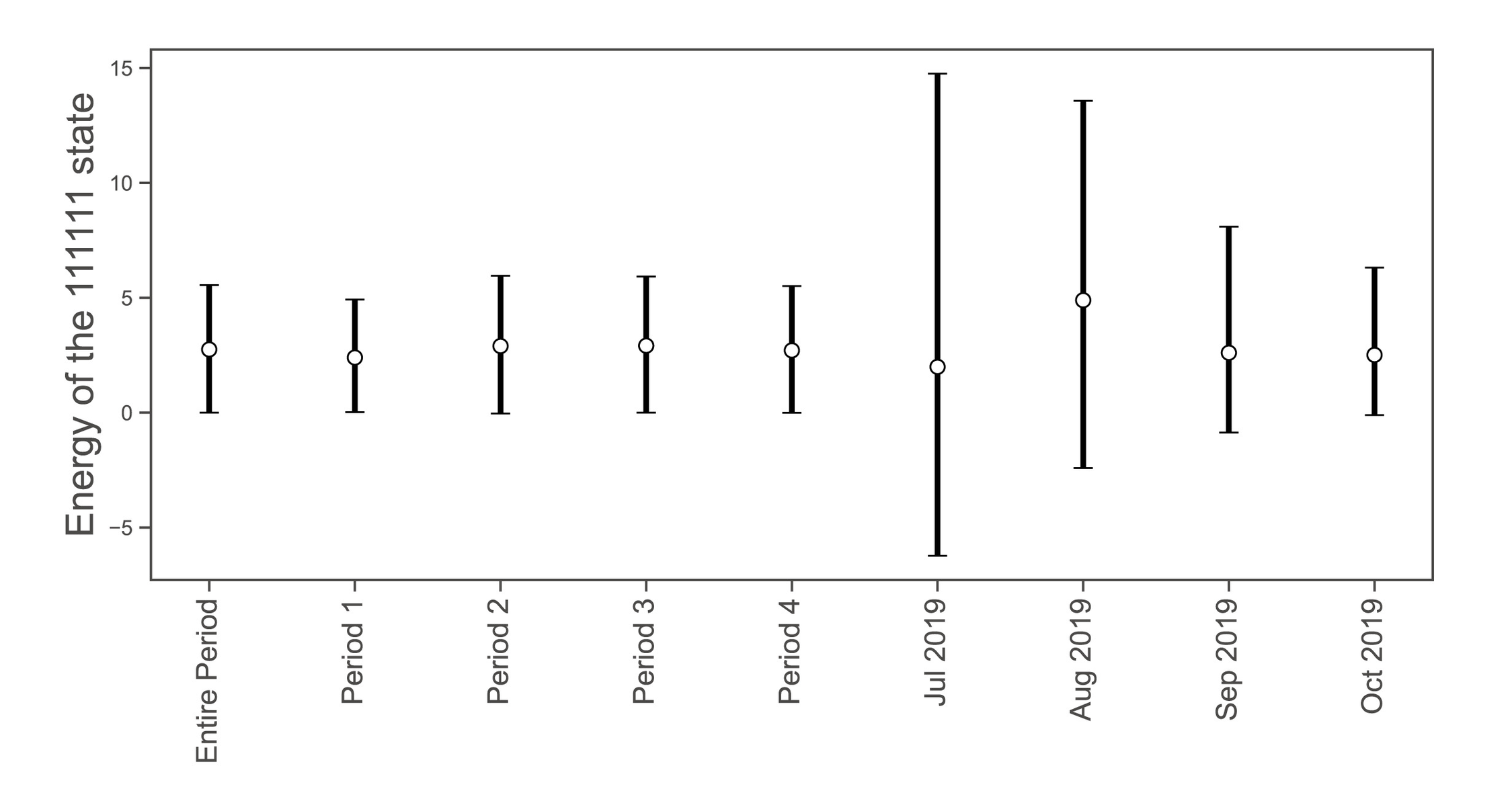
**

**S15 Fig | Confidence intervals of energy estimates:** The 95% confidence interval of the estimated energy of the 111111 state is displayed for the entire period, for Periods 1-4, and for July to Oct 2019. 111111 (binarized responses of 1 to all six items) represents the “depressive” state. The estimation was performed 100 times using bootstrapped data. The permissible error $\varepsilon$ was set to $0.005$ for all conditions; the learning rate $\alpha$ was set to $0.001$ for the entire period and for Periods 1-4 and to $0.01$ for the monthly data.
