# Supplementary material for: Psychological distress among Japanese high school students during the COVID-19 pandemic: An energy landscape analysis
Source: PLoS Med. 2026 Jan 22;23(1):e1004884. doi: 10.1371/journal.pmed.1004884 (PMC12826503; doi:10.1371/journal.pmed.1004884)
Supplement: S16 Fig — (DOCX) [file pmed.1004884.s016.docx]

**
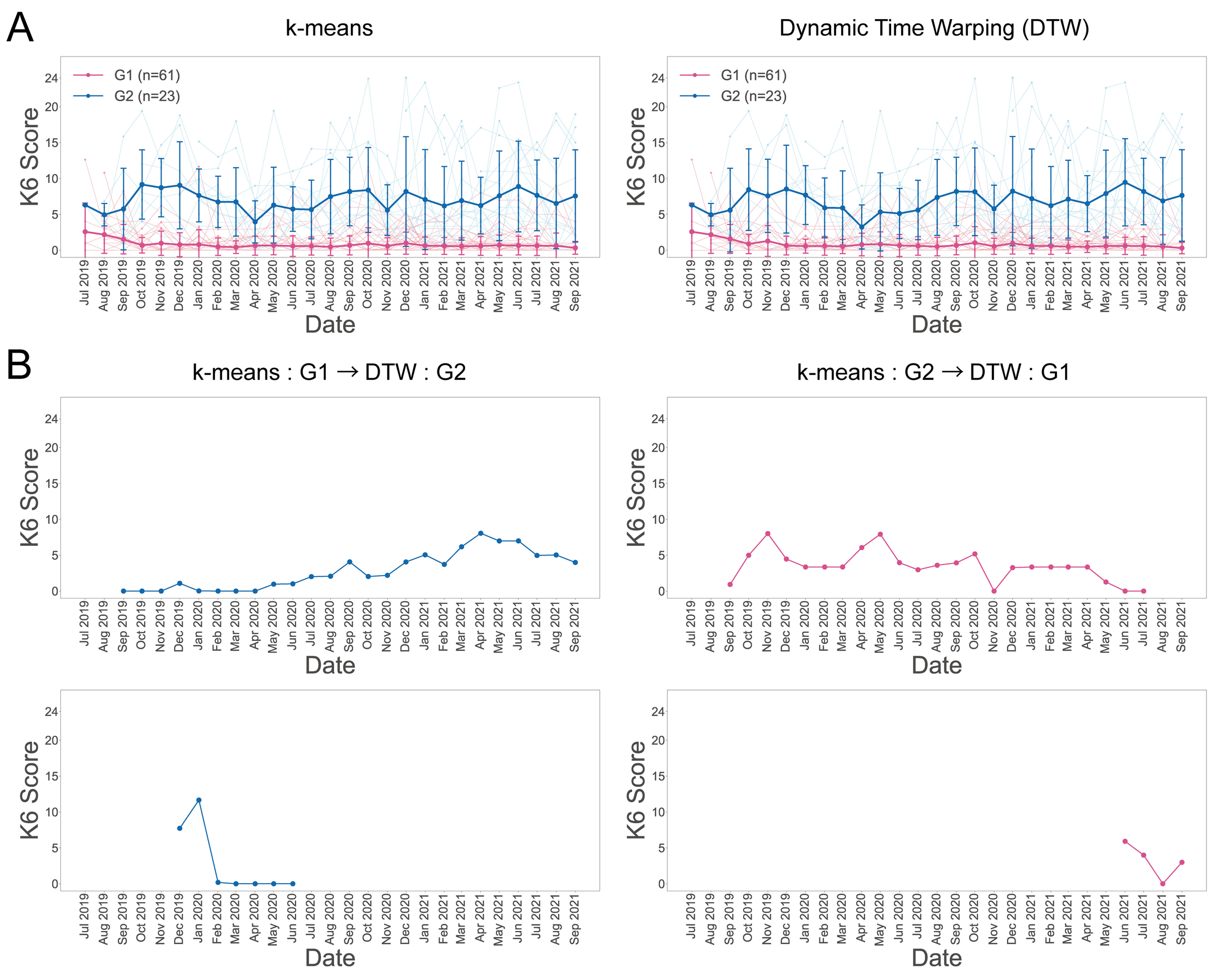
**

**S16 Fig | Clustering of participants by dynamic time warping: (A)** Agreement between two clustering methods (k-means and dynamic time warping). Total K6 scores are plotted with time for each participant. The thick line represents the mean. Error bars indicate standard deviation (SD). The colors correspond to the stratified groups G1 and G2. **(B)** Four individuals are shown that belonged to different clusters according to the two methods.
