# Supplementary material for: Psychological distress among Japanese high school students during the COVID-19 pandemic: An energy landscape analysis
Source: PLoS Med. 2026 Jan 22;23(1):e1004884. doi: 10.1371/journal.pmed.1004884 (PMC12826503; doi:10.1371/journal.pmed.1004884)
Supplement: S17 Fig — (DOCX) [file pmed.1004884.s017.docx]

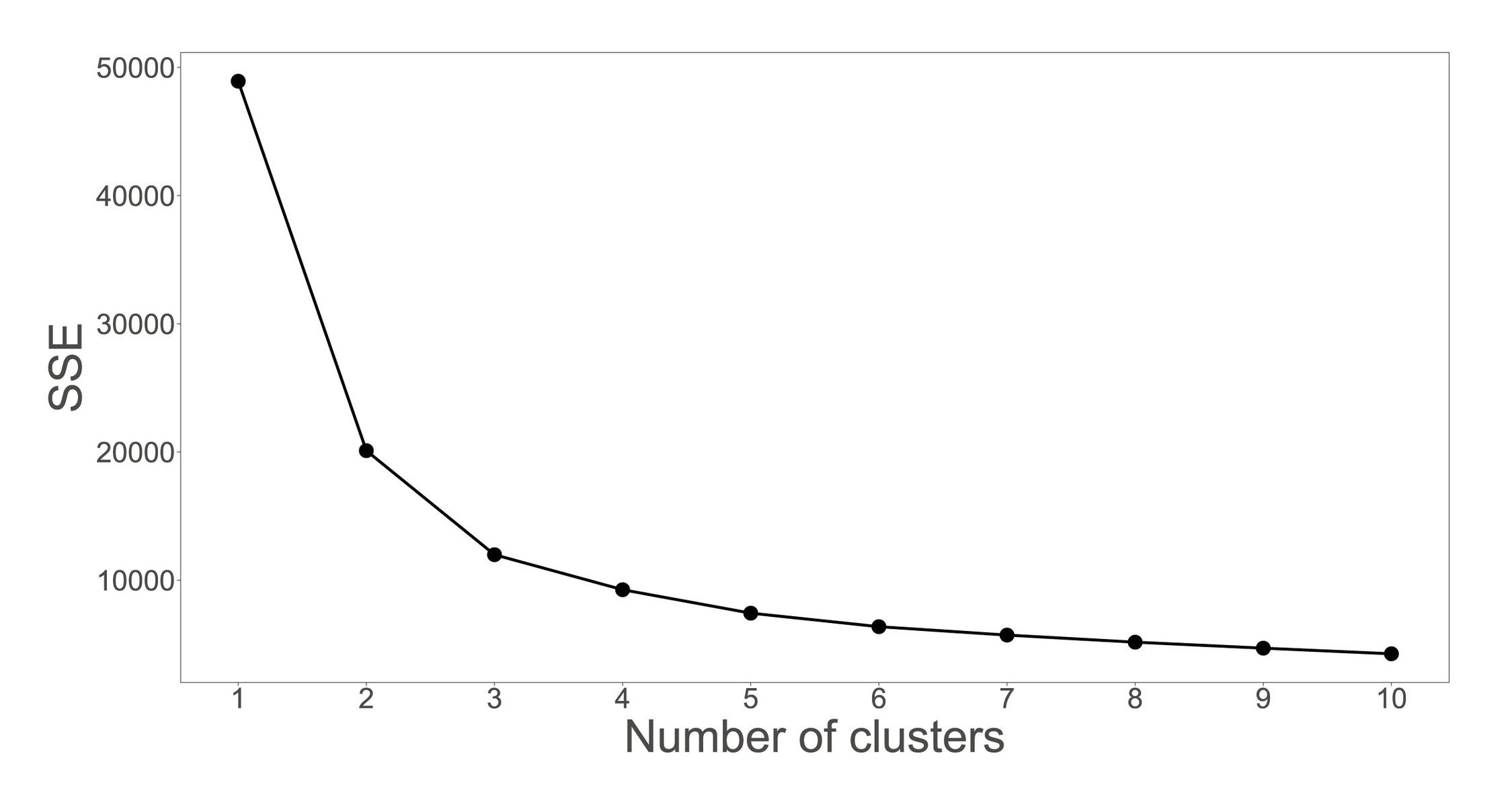
**S17 Fig |** **Accuracy of the clustering:** The within-cluster sum of squared errors (SSE) is used to measure the accuracy of clustering. The number of clusters is varied from 1 to 10.
