# Supplementary material for: Psychological distress among Japanese high school students during the COVID-19 pandemic: An energy landscape analysis
Source: PLoS Med. 2026 Jan 22;23(1):e1004884. doi: 10.1371/journal.pmed.1004884 (PMC12826503; doi:10.1371/journal.pmed.1004884)
Supplement: S18 Fig — (DOCX) [file pmed.1004884.s018.docx]

**
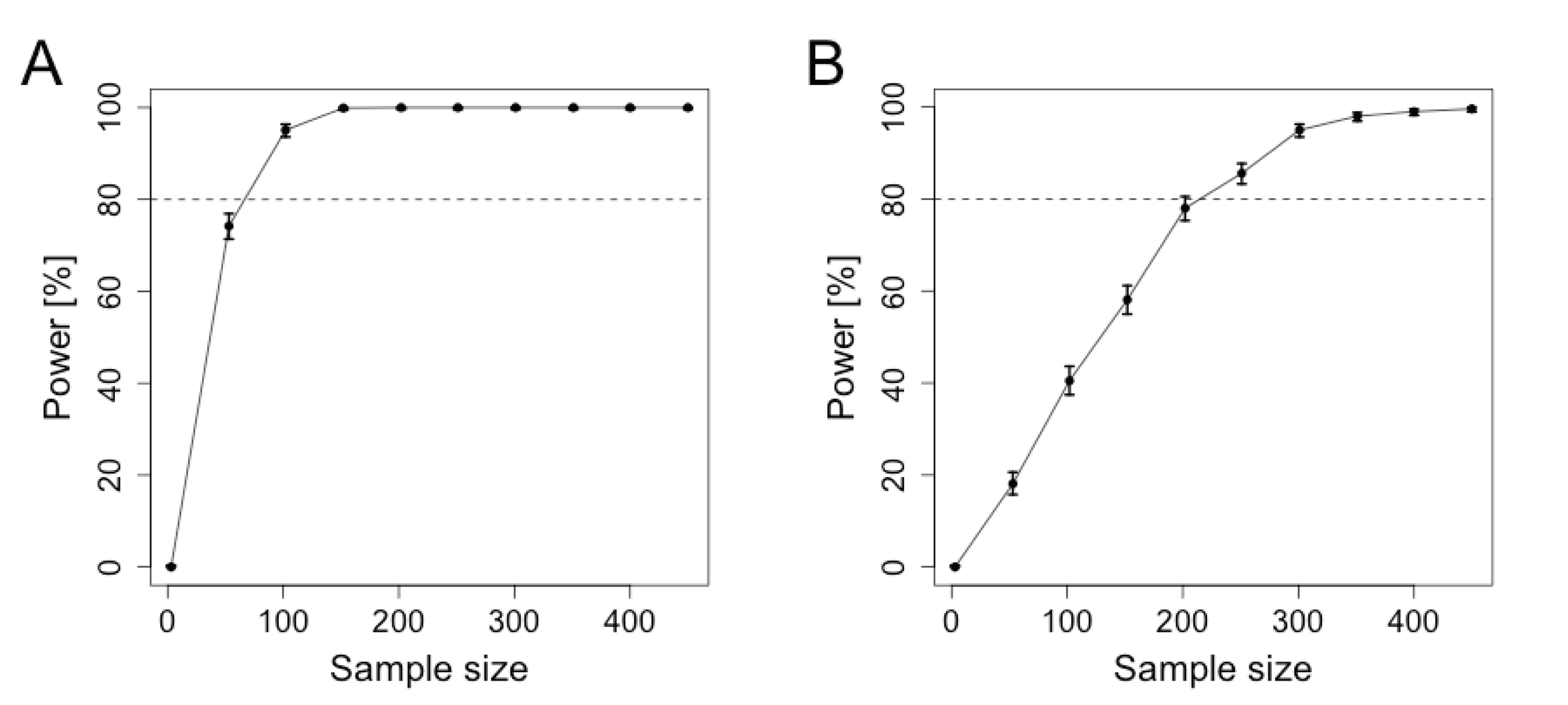
**

**S18 Fig | Estimated power at a range of sample sizes:** Estimated power is plotted for the following sample sizes: 3, 53, 102, 152, 202, 251, 301, 351, 400, 450. The horizontal axis shows the sample size (number of participants) and the vertical axis shows the corresponding power. Error bars indicate standard deviation (SD). **(A)** The model for cortical thickness in the caudal middle prefrontal cortex. **(B)** The model for cortical thickness in the temporal pole.
