# Supplementary material for: Psychological distress among Japanese high school students during the COVID-19 pandemic: An energy landscape analysis
Source: PLoS Med. 2026 Jan 22;23(1):e1004884. doi: 10.1371/journal.pmed.1004884 (PMC12826503; doi:10.1371/journal.pmed.1004884)
Supplement: S1 Note — (DOCX) [file pmed.1004884.s019.docx]

**S1 Note: Glossary of energy landscape analysis terms**

This glossary contains frequently used terms related to energy landscape analysis that are explained in the context of this paper. *Words in italics* have their own glossary entry.

**Adjacent state:** An adjacent state of *state* A is a state that differs from A by only one bit. For example, there are six adjacent states of state 100001, that is, 000001, 110001, 101001, 100101, 100011, and 100000. If state B is an adjacent state of state A, then states A and B are called adjacent states.

**Basin (or basin of attraction):** A basin (or basin of attraction) is a set of *states* that are "attracted" to the same *stable state*, i.e., if we start from a state in an *energy landscape* and keep moving in the direction of lowest *energy*, we will eventually reach that stable state. Thus, stable states lie at the bottom of a basin. States that belong to a basin have properties similar to those of the stable state to which they are attracted. In reality, state transitions do not always occur in the direction of lowest energy, and we can move from one basin to another.

**Disconnectivity graph:** The disconnectivity graph of an *energy landscape* is a dendrogram (binary tree diagram) showing hierarchical relationships among *stable states* of a system. The disconnectivity graph contains leaves and branches. Leaves are stable states, and branches are edges connecting leaves. The vertical position of leaves represents their *energy*. The height of the branch between leaves A and B describes the *energy barrier* that must be overcome to move from A to B or from B to A. See **Fig 2A**.

**Energy:** The energy of a *state* is a real number related to the probability of the state. States in a system can be compared by their energy: if the energy is small ("low energy"), the probability is high; if the energy is large ("high energy"), the probability is low. This energy has nothing to do with the physical energy of the participants.

**Energy barrier:** When an individual moves from one *stable* *state* A to another stable state B in an *energy landscape*, that person passes through an *intermediate state* with higher energy than state A or B. The energy difference between stable state A (starting point) and the intermediate state is called the energy barrier.

**Energy landscape:** An energy landscape is the network of all *states* in a system, where each state is assigned a real number called *energy* and *adjacent states* are connected by an edge. In this paper, the energy landscape is represented graphically as either a *disconnectivity graph* or a *graph of basins*.

**Graph of basins (or graph of basins of attraction):** The graph of basins is a network describing all *basins* and has a forest structure consisting of multiple trees (each tree is a basin). In the graph of basins, each *state* is connected by an edge to an *adjacent state* with the lowest *energy*. Note that not all adjacent states are connected. If we start from a state in a graph of basins and keep moving in the direction of lowest energy, we will eventually reach a *stable state*.

**Intermediate state:** When an individual moves from one *stable* *state* A to another stable state B in an *energy landscape*, that person passes through a state with higher *energy* than either state A or B. This state is called the intermediate state.

**Ising model:** The Ising model is a mathematical model originally proposed to describe ferromagnetism. The model consists of discrete variables, each of which can be in one of two *states*. These variables interact with each other and change the *energy* of the system.

**Numerical simulation**: Based on the obtained *energy landscape*, we can perform numerical simulations of hypothetical transitions between *states*. Since the transition is based on probabilities, it changes from one trial to another, but we can obtain statistical information by running many trials.

**Probability of being in a basin:** The probability of being in a *basin* in a given period is defined as the frequency of *states* belonging to the basin when we pool all the states of participants in a given period.

**Stable state:** A stable state is a *state* in a system that has lower *energy* (thus higher probability) than all *adjacent states*.

**State:** The state of an individual is the binarized responses to the K6 questionnaire at a given time, expressed as a six-bit number, e.g., 110001. A person's state may change over time. In an *energy landscape*, a state is visualized as a point in space.

**Transition probability from one basin to another:** Suppose an *energy landscape* consists of two *basins*, P and Q. Then, the transition probability from basin P to basin Q in a given period is the probability that an individual's state belongs to basin Q the following month, given that that person's state belongs to basin P some time during the period. The transition probabilities from P to P, Q to Q, and Q to P are defined similarly.
