# Supplementary material for: Psychological distress among Japanese high school students during the COVID-19 pandemic: An energy landscape analysis
Source: PLoS Med. 2026 Jan 22;23(1):e1004884. doi: 10.1371/journal.pmed.1004884 (PMC12826503; doi:10.1371/journal.pmed.1004884)
Supplement: S2 Note — (DOCX) [file pmed.1004884.s020.docx]

**S2 Note: Comparison of different binarization and smoothing methods**

In the main text, the individual mean of the K6 score was used to binarize the K6 scores for each item (1 if above the mean, 0 otherwise). For comparison, we also performed different binarization methods: (1) the individual median, (2) the threshold of 1, (3) the threshold of 0.833 (i.e., 5/6). The cutoff values of 1 or 0.833 (=5/6) were chosen because: (a) the mean value of each item of K6 was less than 1, so it was 1 when rounded to an integer; (b) we considered the clinical cutoff value of 5 [[1](#_ENREF_1)] in the total K6 score. The obtained disconnectivity graphs for the four Periods are shown in **S5A-C Fig**. The result of (1) (**S5A Fig**) was qualitatively similar to **Fig 2F**. The results of (2) and (3) (**S5BC Fig**) were also similar to **Fig 2F**, except for a minor difference (the energy of 111111 in Period 4 was smaller than that in Period 1), suggesting that the participants not only became healthier than in the previous periods of school closure (as in **Fig 2F** and the main text), but even healthier than before the pandemic.

To smooth the time series of K6 scores with irregular time points, we used Gaussian process regression to faithfully reflect the rapid fluctuations of the K6 scores by adjusting the kernel function. For comparison, we also performed smoothing by spline curves (**S4A Fig**). The values obtained were mostly similar to those obtained by Gaussian process regression (**S4B Fig**) and did not affect the binarization results (**S4C Fig**).

**References**

1. Furukawa TA, Kawakami N, Saitoh M, Ono Y, Nakane Y, Nakamura Y, et al. The performance of the Japanese version of the K6 and K10 in the World Mental Health Survey Japan. Int J Methods Psychiatr Res. 2008;17(3):152-8. doi: 10.1002/mpr.257. PubMed PMID: 18763695; PubMed Central PMCID: PMCPMC6878390.
