# Supplementary material for: Psychological distress among Japanese high school students during the COVID-19 pandemic: An energy landscape analysis
Source: PLoS Med. 2026 Jan 22;23(1):e1004884. doi: 10.1371/journal.pmed.1004884 (PMC12826503; doi:10.1371/journal.pmed.1004884)
Supplement: S3 Note — (DOCX) [file pmed.1004884.s021.docx]

**S3 Note: Validation of energy landscape analysis using latent class analysis**

To further validate the findings obtained from the energy landscape analysis, we conducted a latent class analysis (LCA) of total K6 scores to quantitatively assess depressive states across the 4 periods (**S11 Fig**). Although we examined information criteria such as AIC and BIC (2-class: AIC=-29464 and BIC=-29595, 3-class: AIC=-32291 and BIC=-32490), we focused on the 2- and 3-class solutions in line with methodological recommendations that emphasize substantive interpretability and clinical plausibility over purely statistical fit. Prior methodological work has shown that information criteria - particularly AIC in large samples - tend to favor over-complex solutions, and that class enumeration should therefore be guided by the meaningfulness and stability of the resulting classes rather than by fit indices alone. Consistent with systematic reviews in psychology and public-health research [[1](#_ENREF_2), [2](#_ENREF_3)], 2- or 3-class solutions are also the most commonly adopted in practice. In the 2-class solution, Class 1 represented a healthy group with nearly zero K6 scores, while Class 2 represented a depressive group with elevated scores. In the 3-class solution, Class 1 again comprised healthy cases with near-zero scores, Class 2 reflected a moderately distressed group, and Class 3 captured the most distressed group with the highest scores (**S11A Fig**).

The energy landscape analysis revealed that the energy of the depressive state (111111) was higher during Periods 2-4 than in Period 1, indicating that the likelihood of being in a depressive state decreased after the onset of the pandemic (**Fig 2F**). To examine whether this temporal pattern could also be captured by LCA, we evaluated how the proportion of datapoints assigned to depressive classes varied across the 4 periods. The results from both the 2-class and 3-class models exhibited patterns consistent with those identified by the energy landscape analysis. In the 2-class model, the proportion of depressive datapoints (Class 2) was higher in Period 1 than in the subsequent periods (0.66, 0.49, 0.49, and 0.50). In the 3-class model, the proportion of moderately depressive datapoints (Class 2) increased, whereas the proportion of healthy datapoints (Class 1) decreased in Period 1 relative to the other periods (Class 1: 0.34, 0.51, 0.51, and 0.49, Class 2: 0.49, 0.32, 0.32, and 0.34) (**S11B Fig**). These findings reproduce the temporal shifts identified by the energy landscape analysis.

To further quantify the agreement between the two methods, we evaluated the relationship between the energy of the depressive state (111111) estimated by the energy landscape analysis and the proportion of depressive datapoints identified by LCA—specifically, Class 2 in the 2-class model and Classes 2 and 3 in the 3-class model—for the 4 periods. A strong negative correlation was observed ($r=-0.95$, $p=0.047$; $r=-0.96$, $p=0.043$; **S11C Fig**). Because higher energy reflects a lower likelihood of being in that state, this negative correlation indicates that the proportion of depressive datapoints identified by LCA is positively associated with the frequency of the depressive state estimated by the energy landscape analysis. These consistent patterns across both analytical approaches support the validity and robustness of the energy landscape results.**References**

1. Sorgente A, Caliciuri R, Robba M, Lanz M, Zumbo BD. A systematic review of latent class analysis in psychology: Examining the gap between guidelines and research practice. Behav Res Methods. 2025;57(11):301. Epub 20251003. doi: 10.3758/s13428-025-02812-1. PubMed PMID: 41044287; PubMed Central PMCID: PMCPMC12494633.

2. Sinha P, Calfee CS, Delucchi KL. Practitioner's Guide to Latent Class Analysis: Methodological Considerations and Common Pitfalls. Crit Care Med. 2021;49(1):e63-e79. doi: 10.1097/CCM.0000000000004710. PubMed PMID: 33165028; PubMed Central PMCID: PMCPMC7746621.
