# Supplementary material for: Psychological distress among Japanese high school students during the COVID-19 pandemic: An energy landscape analysis
Source: PLoS Med. 2026 Jan 22;23(1):e1004884. doi: 10.1371/journal.pmed.1004884 (PMC12826503; doi:10.1371/journal.pmed.1004884)
Supplement: S4 Note — (DOCX) [file pmed.1004884.s022.docx]

**S4 Note: Further statistical analysis on probabilities of staying in the basin and of transitioning between basins**

Using the basins derived from the energy landscape for the entire period (**Fig 2C**), we proceeded to examine the differences in the probability of being in these basins across the four periods (see **Methods**). The probability of being in the 000000 basin (i.e., the frequency of students staying in the healthier states in a given period) was lower in Period 1 than in the other periods (**S13A Fig**), indicating that the COVID-19 pandemic increased the likelihood of being in a healthier state. Similarly, we analyzed the differences in transition probabilities per month between basins (i.e., the relative frequency with which students in one basin stayed in the same basin or moved to the other basin in the next month) for the four periods. Participants in the 000000 basin in a given month predominantly stayed in the same basin in the following month (top panel in **S13B Fig**), while participants in the 111111 basin tended to move to the 000000 basin (bottom panel in **S13B Fig**). Notably, we observed a lower probability of transitioning to the 000000 basin in Period 1 than in the other periods. This illustrates that the COVID-19 pandemic prompted a shift from a more depressive state to a healthier one.

We constructed separate energy landscapes for G1 and G2 and compared the basins derived from the energy landscapes (**Fig 13CD**). The stable states for both groups were 000000 (i.e., healthy state) and 111110/111111 (i.e., depressive(-like) state). The sizes for the 000000 and 111110/111111 basins were 47 and 17 for G1, and 33 and 31 for G2, respectively. Initially, we examined the probability of being in each basin (**S13CD** **Fig**): G1 participants were predominantly in the 000000 basin, whereas G2 participants were in both basins, with a higher probability of the 000000 basin in Periods 2 and 4 coinciding with declarations of states of emergency. We also analyzed the transition probabilities per month between basins: G1 participants in the 000000 basin almost always remained in the same basin, and those in the 111110 basin had a greater than 50% chance of returning to the 000000 basin the following month, particularly with all individuals reverting to the healthy state during Period 2 (**S13E** **Fig**). In contrast, G2 participants in the 000000 basin had a greater than 50% chance of staying in the same basin, with a higher probability of remaining during Periods 2 and 4. However, participants in the 111111 basin were more likely to stay in the 111111 basin, with a gradually decreasing probability from Period 1 to 4 (**S13F** **Fig**). In summary, these results demonstrate that G1 participants mainly remained in the healthier basin, while G2 participants alternated between both basins, with a higher probability of the healthier basin in Periods 2 and 4. Note that the total K6 score showed frequent fluctuations in G2 participants (**Fig 3A**).
