# Supplementary material for: Psychological distress among Japanese high school students during the COVID-19 pandemic: An energy landscape analysis
Source: PLoS Med. 2026 Jan 22;23(1):e1004884. doi: 10.1371/journal.pmed.1004884 (PMC12826503; doi:10.1371/journal.pmed.1004884)
Supplement: S5 Note — (DOCX) [file pmed.1004884.s023.docx]

**S5 Note: Power calculation**

We used the simr package (version 1.0.7) in R to perform a power calculation and estimate the required sample size for MRI findings using random effects models. We ran a simulation 1000 times and set alpha at 0.05 and 0.002, as we applied an FDR correction to the repeated tests for 75 MRI features and obtained significant interactions of age by group in the three models (max threshold of corrected p = 0.05/75*3 = 0.002). In the models for cortical thickness (CT) in the caudal middle prefrontal cortex and the temporal pole, the calculated powers of the interaction of age by group were 98.50% [95% C.I. 97.54 - 99.16] and 72.00% [69.11 - 74.76], respectively, for alpha of 0.05. The power decreased to 85.40% [83.06 - 87.53] and 23.90% [21.29 - 26.67], respectively, for alpha of 0.002. The results showed that at least one significant dependent variable (i.e., CT in the caudal middle prefrontal cortex) had sufficient sensitivity in the data set analyzed (**S18A Fig**), but the model for CT in the temporal pole needs to be confirmed with a larger sample size. Sample size estimation showed that testing with more than 220 participants may provide sufficient power (> 80%) for the latter dependent variable (i.e., CT in the temporal pole) (**S18B Fig**).
