# Supplementary material for: Psychological distress among Japanese high school students during the COVID-19 pandemic: An energy landscape analysis
Source: PLoS Med. 2026 Jan 22;23(1):e1004884. doi: 10.1371/journal.pmed.1004884 (PMC12826503; doi:10.1371/journal.pmed.1004884)
Supplement: S1 Methods — (DOCX) [file pmed.1004884.s024.docx]

**S1 Methods: Data acquisition of population-neuroscience Tokyo TEEN Cohort**

Before participants were enrolled in the pn-TTC Wave 1, the following exclusion criteria were applied: (i) current problems in mental health, interpersonal relationships, or behaviors; (ii) visual or hearing disabilities; (iii) history of head injury accompanied by more than 5 min of loss of consciousness; (iv) current chronic endocrine disease or metabolic disease; and (v) current use of medications that affect the central nervous system [[1](#_ENREF_4)]. The participants in the follow-up Waves 2, 3, and 4 of the pn-TTC study were primarily drawn from individuals who had participated in the previous wave, showed no abnormal brain MRI results, and met the exclusion criteria. To achieve the anticipated recruitment number (approximately 300), additional participants were recruited from the TTC study, applying the same exclusion criteria as in pn-TTC Wave 1.

As of March 2023, a total of 479 pn-TTC participants were recruited from the TTC members. Wave 1 (age 11 years) of the pn-TTC recruitment began in September 2013 and ended in February 2016. For the following Waves 2-4 (age 13, 15, and 18, respectively), participant recruitment was conducted biennially. A total of 1,271 scans from 479 participants were performed between October 2013 and March 2023. After quality controls (QCs) in the image processing steps, a total of 1,211 scans from 471 participants, 247 scans from Wave 1 (age = 11.5 ± 0.7 years; 115 girls), 350 scans from Wave 2 (age = 13.9 ± 0.8 years; 160 girls), 338 scans from Wave 3 (age = 15.9 ± 0.8; 153 girls), and 276 scans from Wave 4 (age = 18.2 ± 0.8; 135 girls) were analyzed in this study.

*Demographic assessment (IQ and SES)*

Participants' intelligence quotient (IQ) at age 10 was assessed using a short version of the Wechsler Intelligence Scale for Children - Third Edition [[2](#_ENREF_5)]. The socioeconomic status (SES) of the children was determined by the higher educational attainment of the father and mother, as assessed by a 6-point scale of educational attainment on the parent survey when the children were age 10 years [[3](#_ENREF_6)].

*Assessment of depressive symptoms on the date of MRI scans (GHQ-28)*

Participants' depressive symptoms on the date of MRI scans for Waves 3 and 4 were assessed using the GHQ-28 [[4](#_ENREF_7)], a widely used scale of subjective depressive symptoms. Each question was constructed using a 4-point Likert scale, and responses were transformed to a score of 0-0-1-1 (range 0-28, with higher scores representing more severe depressive symptoms). The GHQ-28 was originally developed for adults and is validated for use with junior high school adolescents [[5](#_ENREF_8), [6](#_ENREF_9)].

**References**

1. Okada N, Ando S, Sanada M, Hirata-Mogi S, Iijima Y, Sugiyama H, et al. Population-neuroscience study of the Tokyo TEEN Cohort (pn-TTC): Cohort longitudinal study to explore the neurobiological substrates of adolescent psychological and behavioral development. Psychiatry Clin Neurosci. 2019;73(5):231-42. Epub 20190219. doi: 10.1111/pcn.12814. PubMed PMID: 30588712.

2. Wechsler D. The Wechsler intelligence scale for children—third edition. San Antonio, TX: The Psychological Corporation; 1991.

3. Fujikawa S, Ando S, Nishida A, Usami S, Koike S, Yamasaki S, et al. Disciplinary slapping is associated with bullying involvement regardless of warm parenting in early adolescence. J Adolesc. 2018;68:207-16. Epub 20180818. doi: 10.1016/j.adolescence.2018.07.018. PubMed PMID: 30130722.

4. Goldberg DP, Hillier VF. A scaled version of the General Health Questionnaire. Psychol Med. 1979;9(1):139-45. doi: 10.1017/s0033291700021644. PubMed PMID: 424481.

5. Iwata N, Saito K. The factor structure of the 28-item General Health Questionnaire when used in Japanese early adolescents and adult employees: age- and cross-cultural comparisons. Eur Arch Psychiatry Clin Neurosci. 1992;242(2-3):172-8. doi: 10.1007/BF02191565. PubMed PMID: 1486106.

6. Tait RJ, Hulse GK, Robertson SI. A review of the validity of the General Health Questionnaire in adolescent populations. Aust N Z J Psychiatry. 2002;36(4):550-7. doi: 10.1046/j.1440-1614.2002.01028.x. PubMed PMID: 12169157.
