# Supplementary material for: Psychological distress among Japanese high school students during the COVID-19 pandemic: An energy landscape analysis
Source: PLoS Med. 2026 Jan 22;23(1):e1004884. doi: 10.1371/journal.pmed.1004884 (PMC12826503; doi:10.1371/journal.pmed.1004884)
Supplement: S2 Methods — (DOCX) [file pmed.1004884.s025.docx]

**S2 Methods: Acquisition and preprocessing methods for the brain imaging procedures**

*Acquisition methods and brain images*

Three acquisition procedures were used in the pn-TTC study (**S1 Table**). Procedure 1 used a Philips Achieva scanner with an 8-channel head coil and obtained 518 scans at Waves 1 and 2. Procedure 2 used a Siemens Prisma scanner with a 64-channel head coil to obtain 121 scans at Wave 2. The protocols for Procedures 1 and 2 were determined according to previous Japanese multisite projects and had similar scan parameters [1, 2]. Procedure 3 used a Siemens Prisma scanner with a 32-channel head coil, and 632 scans were obtained at Waves 3 and 4. The protocol for Procedure 3 was the same as that provided by the Human Connectome Project (HCP) for lifespan development and aging projects [[3-5](#_ENREF_12)]. From a total of 1,271 brain images, 60 images were excluded on the basis of a manual check for poor image quality by 3 trained staff: 3 images due to mass anatomical incidental findings or brain operation history, 9 images because of a failure of image preprocessing, and 48 images because of poor imaging quality and/or substantial outliers detected by the ENIGMA QC script.

*Image preprocessing*

The images using Procedures 1 and 2 were preprocessed using the legacy style in the HCP pipeline, primarily via the FreeSurfer version 6.0.1 “recon-all” command. The images obtained using Procedure 3 were preprocessed using HCP pipeline version 4.3.0 [[6](#_ENREF_15)]. The pipeline requires both T1w and T2w images and provides greater validity and reliability of cortical thickness, surface area, and subcortical volume. If T2w images were unavailable for any reason, we preprocessed only T1w images using FreeSurfer “recon-all” for Procedures 1 and 2.

*Traveling subject harmonization*

The use of a general linear model (GLM) is the most basic approach for removing site effects. We followed the traveling subject (TS) harmonization method [[2](#_ENREF_11), [7](#_ENREF_16)], which extends the GLM harmonization model using a TS dataset. The TS harmonization model can be described as follows:

$y\left( i,j,v \right)=\boldsymbol{X}_{s}^{T}(i,j)\boldsymbol{\beta}_{s}(v) +\boldsymbol{X}_{p}^{T}(i,j)\boldsymbol{\beta}_{p}(v)\boldsymbol{+\varepsilon}\left( i,j,v \right)$, (1)

where ***β_p_***(*v*) represents the participant factor, and ***X_p_*** (*i*, *j*) is the *n ×* 1 vector of the participant indicator. ***b_s_***(*v*) represents the coefficient of the site factor, namely the measurement bias, and ***X_s_*** (*i*, *j*) is the *k×* 1 vector of the site indicator. To estimate the respective parameters, we calculated the inverse matrix for ***X_p_*** (*i*, *j*) and ***X_s_*** (*i*, *j*). In this study, all subjects were healthy and identical at each site; therefore, sampling bias was not considered. However, the design matrix of the GLM was rank-deficient; thus, we used the Moore–Penrose pseudo-inverse matrix via the “pinv” function in MATLAB (R2020b) to estimate $\hat{\beta}_{s}\left( v \right)$ and $\hat{\beta}_{p}\left( v \right)$. After estimating $\hat{\beta}_{s}\left( v \right)$, the harmonized anatomical volumes and thicknesses were set as follows:

$y^{TS glm}\left( i,j,v \right)=y\left( i,j,v \right)-\boldsymbol{X}_{s}^{T}(i,j){\hat{\boldsymbol{\beta}}}_{s}(v)$. (2)

**References**

1. Tanaka SC, Yamashita A, Yahata N, Itahashi T, Lisi G, Yamada T, et al. A multi-site, multi-disorder resting-state magnetic resonance image database. Sci Data. 2021;8(1):227. Epub 20210830. doi: 10.1038/s41597-021-01004-8. PubMed PMID: 34462444; PubMed Central PMCID: PMCPMC8405782.

2. Yamashita A, Yahata N, Itahashi T, Lisi G, Yamada T, Ichikawa N, et al. Harmonization of resting-state functional MRI data across multiple imaging sites via the separation of site differences into sampling bias and measurement bias. PLoS Biol. 2019;17(4):e3000042. Epub 20190418. doi: 10.1371/journal.pbio.3000042. PubMed PMID: 30998673; PubMed Central PMCID: PMCPMC6472734.

3. Harms MP, Somerville LH, Ances BM, Andersson J, Barch DM, Bastiani M, et al. Extending the Human Connectome Project across ages: Imaging protocols for the Lifespan Development and Aging projects. Neuroimage. 2018;183:972-84. Epub 20180924. doi: 10.1016/j.neuroimage.2018.09.060. PubMed PMID: 30261308; PubMed Central PMCID: PMCPMC6484842.

4. Koike S, Tanaka SC, Okada T, Aso T, Yamashita A, Yamashita O, et al. Brain/MINDS beyond human brain MRI project: A protocol for multi-level harmonization across brain disorders throughout the lifespan. Neuroimage Clin. 2021;30:102600. Epub 20210316. doi: 10.1016/j.nicl.2021.102600. PubMed PMID: 33741307; PubMed Central PMCID: PMCPMC8209465.

5. Somerville LH, Bookheimer SY, Buckner RL, Burgess GC, Curtiss SW, Dapretto M, et al. The Lifespan Human Connectome Project in Development: A large-scale study of brain connectivity development in 5-21 year olds. Neuroimage. 2018;183:456-68. Epub 20180822. doi: 10.1016/j.neuroimage.2018.08.050. PubMed PMID: 30142446; PubMed Central PMCID: PMCPMC6416053.

6. Glasser MF, Sotiropoulos SN, Wilson JA, Coalson TS, Fischl B, Andersson JL, et al. The minimal preprocessing pipelines for the Human Connectome Project. Neuroimage. 2013;80:105-24. Epub 20130511. doi: 10.1016/j.neuroimage.2013.04.127. PubMed PMID: 23668970; PubMed Central PMCID: PMCPMC3720813.

7. Maikusa N, Zhu Y, Uematsu A, Yamashita A, Saotome K, Okada N, et al. Comparison of traveling-subject and ComBat harmonization methods for assessing structural brain characteristics. Hum Brain Mapp. 2021;42(16):5278-87. Epub 20210817. doi: 10.1002/hbm.25615. PubMed PMID: 34402132; PubMed Central PMCID: PMCPMC8519865.
