# Supplementary material for: Psychological distress among Japanese high school students during the COVID-19 pandemic: An energy landscape analysis
Source: PLoS Med. 2026 Jan 22;23(1):e1004884. doi: 10.1371/journal.pmed.1004884 (PMC12826503; doi:10.1371/journal.pmed.1004884)
Supplement: S1 Table — (DOCX) [file pmed.1004884.s026.docx]

**S1 Table: Image acquisition parameters and preprocessing methods**

|  | **Procedure 1** | **Procedure 2** | **Procedure 3** |
| --- | --- | --- | --- |
| Manufacturer  Scanner model  Head coil (ch)  Repetition time (ms)  Echo time (ms)  In-plate resolution (mm^2^)  Matrix size  Slice thickness (mm)  Slice direction  Slice orientation  Pulse sequence  Flip angle (°) | PHILIPS  Achieva  8  7  3.17  1.0×1.0  256*×*256  1.2  AP  Sagittal  MPRAGE  9 | SIEMENS  Prisma  64  1900  2.53  1.0×1.0  256*×*256  1.2  AP  Sagittal  MPRAGE  9 | SIEMENS  Prisma  32  2400  2.22  0.8×0.8  256*×*240  0.8  AP  Sagittal  MPRAGE  8 |
