# Supplementary material for: Psychological distress among Japanese high school students during the COVID-19 pandemic: An energy landscape analysis
Source: PLoS Med. 2026 Jan 22;23(1):e1004884. doi: 10.1371/journal.pmed.1004884 (PMC12826503; doi:10.1371/journal.pmed.1004884)
Supplement: S2 Table — (DOCX) [file pmed.1004884.s027.docx]

**S2 Table: Difference in depressive symptom scores between energy-landscape-related G1/G2 groups**

|  | B | S.E. | z | p |
| --- | --- | --- | --- | --- |
| (Intercept) | -3.09 | 0.66 | -4.70 | < 0.001 |
| GHQ score at Wave 3 | 0.34 | 0.09 | 3.63 | < 0.001 |
| Diff. in GHQ scores | 0.22 | 0.08 | 2.80 | 0.005 |

A logistic regression was tested for the energy landscape-related groups (G1 as reference).
