# Supplementary material for: Psychological distress among Japanese high school students during the COVID-19 pandemic: An energy landscape analysis
Source: PLoS Med. 2026 Jan 22;23(1):e1004884. doi: 10.1371/journal.pmed.1004884 (PMC12826503; doi:10.1371/journal.pmed.1004884)
Supplement: S3 Table — (DOCX) [file pmed.1004884.s028.docx]

**S3 Table: List of the model variables and parameters**

| **Name** | **Symbol** | **Meaning** | **Values** | **Notes** |
| --- | --- | --- | --- | --- |
| State | $\boldsymbol{\sigma= (}\sigma_{1,} \boldsymbol{...,}\sigma_{N}\boldsymbol{)}$ | Binarized response of $N$questionnaire items | $N$-dimensional binary vector | Overall, there are $2^{N}$ possible states*.* |
| Probability of a state (model) | $P_{\text{model}}$ | How often a certain state occurs | Real value | - |
| Energy of a state (model) | $E_{\text{model}}$ | How much energy a certain state has | Real value | Lower (higher) energy corresponds to higher (lower) probability |
| Coefficient of individual items | $\boldsymbol{h}={(h_{1}, ..., h}_{N})$ | The contribution of each item in the energy | $N$-dimensional real vector | - |
| Coefficient of interactions | $\boldsymbol{J}={\boldsymbol{(}J}_{ij})$  $(i,j=1,\ldots,N)$ | The contribution of the interaction between items in the energy | $N\times N$  dimensional real matrix | - |
