# Supplementary material for: Psychological distress among Japanese high school students during the COVID-19 pandemic: An energy landscape analysis
Source: PLoS Med. 2026 Jan 22;23(1):e1004884. doi: 10.1371/journal.pmed.1004884 (PMC12826503; doi:10.1371/journal.pmed.1004884)
Supplement: S4 Table — (DOCX) [file pmed.1004884.s029.docx]

**S4 Table: Estimated coefficients of the Ising model for the entire period**

|  | K6-1 | K6-2 | K6-3 | K6-4 | K6-5 | K6-6 |
| --- | --- | --- | --- | --- | --- | --- |
| K6-1 | $h_{1}=-3.00$0 | - | - | - | - | - |
| K6-2 | $J_{12}=0.368$ | $h_{2}=-2.967$ | - | - | - | - |
| K6-3 | $J_{13}=1.512$ | $J_{23}=0.582$ | $h_{3}=-2.492$ | - | - | - |
| K6-4 | $J_{14}=0.865$ | $J_{24}=1.730$ | $J_{34}=1.256$ | $h_{4}=-2$.748 | - | - |
| K6-5 | $J_{15}=1.047$ | $J_{25}=1.585$ | $J_{35}=0.719$ | $J_{45}=1.052$ | $h_{5}=-3.085$ | - |
| K6-6 | $J_{16}=0.298$ | $J_{26}=1.398$ | $J_{36}=0.500$ | $J_{46}=1.736$ | $J_{56}=0.349$ | $h_{6}=-3.456$ |
