# Supplementary material for: Psychological distress among Japanese high school students during the COVID-19 pandemic: An energy landscape analysis
Source: PLoS Med. 2026 Jan 22;23(1):e1004884. doi: 10.1371/journal.pmed.1004884 (PMC12826503; doi:10.1371/journal.pmed.1004884)
Supplement: S5 Table — (DOCX) [file pmed.1004884.s030.docx]

**S5 Table: Item-level statistical analysis**

|  |  | K6-1 | K6-2 | K6-3 | K6-4 | K6-5 | K6-6 |
| --- | --- | --- | --- | --- | --- | --- | --- |
| Jonckheere-Terpstra  trend test |  | 0.294 | 0.568 | 0.059 | 0.445 | 0.670 | 0.764 |
| Tukey’s test | Period 1 vs. 2 | 0.332 | 0.598 | 0.008 | 0.258 | 0.854 | 1.000 |
|  | Period 1 vs. 3 | 0.047 | 0.978 | 0.161 | 0.491 | 0.972 | 0.984 |
|  | Period 1 vs. 4 | 0.448 | 0.971 | 0.131 | 0.488 | 0.995 | 0.781 |
|  | Period 2 vs. 3 | 0.799 | 0.699 | 0.392 | 0.917 | 0.434 | 0.982 |
|  | Period 2 vs. 4 | 0.964 | 0.673 | 0.343 | 0.876 | 0.531 | 0.708 |
|  | Period 3 vs. 4 | 0.351 | 1.000 | 1.000 | 1.000 | 0.992 | 0.870 |
| Mood's median test |  | 0.125 | 0.958 | 0.591 | 0.318 | 0.990 | 0.644 |
