# Supplementary material for: Psychological distress among Japanese high school students during the COVID-19 pandemic: An energy landscape analysis
Source: PLoS Med. 2026 Jan 22;23(1):e1004884. doi: 10.1371/journal.pmed.1004884 (PMC12826503; doi:10.1371/journal.pmed.1004884)
Supplement: S6 Table — (DOCX) [file pmed.1004884.s031.docx]

**S6 Table: Number of participants, categorized by the mean total K6 scores, in each Period**

**G1 / G2**

| K6 score | 0 ≤ score < 5 | 5 ≤ score < 8 | 8 ≤ score < 13 | 13 ≤ score |
| --- | --- | --- | --- | --- |
| Period 1 | 31 / 3 | 1 / 1 | 0 / 4 | 0 / 1 |
| Period 2 | 45 / 5 | 0 / 5 | 0 / 4 | 0 / 0 |
| Period 3 | 50 / 6 | 1 / 4 | 0 / 4 | 0 / 2 |
| Period 4 | 52 / 8 | 1 / 4 | 0 / 3 | 0 / 4 |
