# Supplementary material for: Psychological distress among Japanese high school students during the COVID-19 pandemic: An energy landscape analysis
Source: PLoS Med. 2026 Jan 22;23(1):e1004884. doi: 10.1371/journal.pmed.1004884 (PMC12826503; doi:10.1371/journal.pmed.1004884)
Supplement: S7 Table — (DOCX) [file pmed.1004884.s032.docx]

**S7 Table: Demographic characteristics in energy-landscape-related G1 and G2 groups**

|  | **G1** |  | **G2** |  | **p** |
| --- | --- | --- | --- | --- | --- |
| **n** | 61 |  | 23 |  |  |
| **Female (n (%))** | **25** | **(41.0)** | **17** | **(73.9)** | **0.014** |
| **IQ (mean (SD))** | 108.77 | (13.01) | 109.87 | (11.74) | 0.72 |
| **Parental SES (%)** |  |  |  |  | 0.63 |
| **High school** | 3 | (4.9) | 3 | (13.0) |  |
| **2-year college** | 7 | (11.5) | 2 | (8.7) |  |
| **4-year university** | 45 | (73.8) | 16 | (69.6) |  |
| **Graduate university** | 6 | (9.8) | 2 | (8.7) |  |
| **GHQ score (mean (SD))** |  |  |  |  |  |
| **Wave 3** | **3.93** | **(3.94)** | **9.2** | **(5.88)** | **<0.001** |
| **Wave 4** | **3.19** | **(3.28)** | **10.05** | **(6.31)** | **<0.001** |
| **Difference between waves** | -0.75 | (4.34) | 0.18 | (6.01) | 0.49 |
