# Supplementary material for: Psychological distress among Japanese high school students during the COVID-19 pandemic: An energy landscape analysis
Source: PLoS Med. 2026 Jan 22;23(1):e1004884. doi: 10.1371/journal.pmed.1004884 (PMC12826503; doi:10.1371/journal.pmed.1004884)
Supplement: S8 Table — (DOCX) [file pmed.1004884.s033.docx]

**S8 Table: Relationship of three psychological features with the cortical thickness in the caudal middle frontal gyrus (cMFG)**

|  | B | S.E. | df | t | p |
| --- | --- | --- | --- | --- | --- |
| (Intercept) | 0.035 | 0.035 | 230 | 1.01 | 0.31 |
| G1/G2 label | 0.0055 | 0.0095 | 230 | 0.57 | 0.57 |
| GHQ at Wave 3 | -0.00055 | 0.00109 | 230 | -0.53 | 0.60 |
| Diff. in GHQ scores | -0.00026 | 0.00099 | 230 | -0.27 | 0.79 |
| IQ | -0.00012 | 0.00028 | 230 | -0.44 | 0.66 |
| SES | -0.0044 | 0.0050 | 230 | -0.89 | 0.38 |
| Handedness |  |  |  |  |  |
| L | 0.0039 | 0.0123 | 230 | 0.31 | 0.75 |
| R | 0.00019 | 0.00766 | 230 | 0.02 | 0.98 |
| Age interaction |  |  |  |  |  |
| G1/G2 label | **-0.023** | **0.009** | **230** | **-2.36** | **0.019** |
| GHQ at Wave 3 | -0.000035 | 0.000726 | 230 | -0.05 | 0.96 |
| Diff. in GHQ scores | -0.00050 | 0.00087 | 230 | -0.57 | 0.57 |

Bold shows p < 0.05.
