# Supplementary material for: Psychological distress among Japanese high school students during the COVID-19 pandemic: An energy landscape analysis
Source: PLoS Med. 2026 Jan 22;23(1):e1004884. doi: 10.1371/journal.pmed.1004884 (PMC12826503; doi:10.1371/journal.pmed.1004884)
Supplement: S9 Table — (DOCX) [file pmed.1004884.s034.docx]

**S9 Table: Relationship of three psychological features with the cortical thickness in the temporal pole (TP)**

|  | B | S.E. | df | t | p |
| --- | --- | --- | --- | --- | --- |
| (Intercept) | 0.034 | 0.118 | 230 | 0.29 | 0.77 |
| G1/G2 label | -0.018 | 0.032 | 230 | -0.56 | 0.57 |
| GHQ at Wave 3 | 0.00012 | 0.00349 | 230 | 0.03 | 0.97 |
| Diff. in GHQ scores | -0.00055 | 0.00331 | 230 | -0.17 | 0.87 |
| IQ | -0.00063 | 0.00094 | 230 | -0.67 | 0.50 |
| SES | 0.0088 | 0.0169 | 230 | 0.52 | 0.60 |
| Handedness |  |  |  |  |  |
| L | 0.016 | 0.041 | 230 | 0.38 | 0.71 |
| R | 0.0003 | 0.0257 | 230 | 0.01 | 0.99 |
| Age interaction |  |  |  |  |  |
| G1/G2 label | **0.10** | **0.03** | **230** | **3.08** | **0.0023** |
| GHQ at Wave 3 | **-0.0057** | **0.0024** | **230** | **-2.35** | **0.020** |
| Diff. in GHQ scores | -0.0030 | 0.0029 | 230 | -1.03 | 0.30 |

Bold shows p < 0.05.
