# Supplementary material for: Psychological distress among Japanese high school students during the COVID-19 pandemic: An energy landscape analysis
Source: PLoS Med. 2026 Jan 22;23(1):e1004884. doi: 10.1371/journal.pmed.1004884 (PMC12826503; doi:10.1371/journal.pmed.1004884)
Supplement: Attachment — Submitted filename: PBP_revision.docx [file pmed.1004884.s039.docx]

**Response to editor and reviewer comments**

Daiki Tatematsu, Naotoshi Nakamura, Masato Abe, Tetsuo Ishikawa, Takahiro Ezaki, Lin Cai, Eiryo Kawakami, Kazuyuki Aihara, Atsushi Nishida, Naohiro Okada, Naoki Masuda, Kiyoto Kasai, Shinsuke Koike, Shingo Iwami

June 24, 2025

We would like to thank the reviewers for their thorough reading of the manuscript and their helpful comments and suggestions. In this document, we present the reviewers’ comments in black and provide our responses in red. All changes made in response to the reviewers’ comments in PMEDICINE-D-24-03134R1 have been incorporated in the revised version of the manuscript. We believe that the manuscript has been considerably improved by these changes. All co-authors have agreed with these changes.

**Editor comments:**

The editorial team agrees that reconsideration of the original decision will only be possible once you have responded to the two comments below:

1) We feel that the energy landscape analysis has value and potential, but due to its limited use in psychology and psychiatry, it would be of paramount importance that readers have a good, intuitive understanding of the method and why it is a good method for the data structure.

**Reply to Editor comment 1:**

For readers in the fields of psychology and psychiatry who are not familiar with statistical physics, we provided a glossary of energy landscape analysis terms in **Supplementary Note 2**, in addition to detailed explanations using mathematical formulas in the **Methods**. To further promote an intuitive understanding, we included an illustrated explanation of energy landscape analysis in **Supplementary Fig 3**. Furthermore, on page 19, lines 353-358, we provided an intuitive explanation of the Ising model, an important concept in energy landscape analysis, and discussed it in the context of questionnaire data. These additions should help readers understand that energy landscape analysis is a useful method for capturing the data structure of questionnaires.

2) We would ask you to include, for example as an additional/sensitivity analysis, an analysis that analyses the same data using a more common, validated method.

**Reply to Editor comment 2:**

For comparison with energy landscape analysis, we performed an additional analysis using more common, validated statistical methods, namely the Jonckheere-Terpstra trend test, Tukey's test, and Mood's median test. Our analysis was validated by Jonckheere-Terpstra trend test using the total K6 score (**Fig 2E**). However, the total K6 score alone does not reveal which combination of items contributed to the healthy and depressive states, nor how these states change over time. When the K6 scores were analyzed item by item, however, these traditional analyses did not reveal significant differences among the different periods (see **Supplementary Table 4**), suggesting that these methods were not sensitive enough to detect item-level differences. We mentioned this on page 24, lines 428-432. In contrast, energy landscape analysis can capture relationships between multiple questionnaire items by fully exploiting the multidimensional structure and dynamic features, as mentioned on page 19, lines 353-358.

**Editor comments:**

If you feel that you can address these points and would like us to consider your appeal, please provide the following documentation:

1. Response to reviewers: In a separate file, provide a point-by-point response to each of the comments in your decision letter.

2. If you made any changes to your manuscript, please also provide a 'Track Changes' version of your paper that highlights any updates you made.

**Reply to Editor comments:**

Below is a list of the revised files, along with a brief description of the changes made to each.

1. Response to Reviewer comments (this file)

2. Manuscript file

- New analyses and descriptions were added to the Results, Discussion, and Methods (see replies to comments below for details).

3. Marked-up manuscript file ('Track Changes' version)

- The text added in revision is shown in red.

4. Supplementary file

- New figures were added as Supplementary Figures as follows. We also merged the Extended Data Figures into the Supplementary Figures in accordance with the PLOS guidelines and relabeled them as Supplementary Figures.

**Supplementary Figure 1** | Summary of the whole study

**Supplementary Figure 5** | Changes in total K6 scores across different periods

**Supplementary Figure 10** | Confidence intervals of energy estimates

**Supplementary Figure 12** | Different binarization methods

**Supplementary Figure 13** | Different smoothing methods

**Supplementary Figure 14** | Clustering of participants by dynamic time warping

**Supplementary Figure 15** | Estimated power at a range of sample sizes

- Furthermore, new Supplementary Notes, Supplementary Tables, and Supplementary Checklists were added as follows:

**Supplementary Note 1** | Comparison of different binarization and smoothing methods

**Supplementary Note 4** | Power calculation

**Supplementary Table 2** | List of the model variables and parameters

**Supplementary Table 3** | Estimated coefficients of the Ising model for the entire period

**Supplementary Table 4** | Item-level statistical analysis

**Supplementary Table 5** | Number of participants, categorized by the mean total K6 scores, in each Period

**Supplementary Table 6** | Demographic characteristics in energy-landscape-related G1 and G2 groups

**S1 Checklist** | STROBE Checklist

**S2 Checklist** | TRIPOD-AI Checklist

**Journal Requirements:**

1) Please ensure that the funders and grant numbers match between the Financial Disclosure field and the Funding Information tab in your submission form. Note that the funders must be provided in the same order in both places as well. Currently, order of grants are different in both places.

**Reply to Journal Requirement 1:**

We edited the submission form and matched the content and order in both fields.

2) Please amend your detailed Financial Disclosure statement. This is published with the article, therefore should be completed in full sentences and contain the exact wording you wish to be published.

i). State what role the funders took in the study. If the funders had no role in your study, please state: “The funders had no role in study design, data collection and analysis, decision to publish, or preparation of the manuscript.”

ii). If any authors received a salary from any of your funders, please state which authors and which funders.

**Reply to Journal Requirement 2:**

We added the following statement on page 48, lines 987-988: “The funders had no role in study design, data collection and analysis, decision to publish, or preparation of the manuscript”.

3) We ask that a manuscript source file is provided at Revision. Please upload your manuscript file as a .doc, .docx, .rtf or .tex.

**Reply to Journal Requirement 3:**

We uploaded our manuscript file as a .docx.

4) Please provide separate figure files in .tif or .eps format.

For more information about figure files please see our guidelines:

https://journals.plos.org/plosmedicine/s/figures

https://journals.plos.org/plosmedicine/s/figures#loc-file-requirements

**Reply to Journal Requirement 4:**

We uploaded separate figure files in the .tif format.

5) Thank you for stating " The data will be available after a review process by the pn-TTC projects. Please direct requests for data to the corresponding author. " Please note that the contact for accessing your data cannot be an author on the manuscript. Please therefore replace the current contact information with a third party contact, such as an institutional data access committee.

**Reply to Journal Requirement 5:**

The statement was updated as follows on page 48, lines 960-962: “The data will be available after a review process by the pn-TTC projects. Please direct requests for data to Department of Neuropsychiatry, The University of Tokyo (https://value.umin.jp/data-resource.html).”

6) We notice that your supplementary [figures and tables] are included in the manuscript file. Please remove them and upload them with the file type 'Supporting Information'. Please ensure that each Supporting Information file has a legend listed in the manuscript after the references list.

**Reply to Journal Requirement 6:**

We removed Supplementary Figures and Tables from the manuscript and uploaded them as 'Supporting Information'.

7) "Some material included in your submission may be copyrighted. According to PLOS’s copyright policy, authors who use figures or other material (e.g., graphics, clipart, maps) from another author or copyright holder must demonstrate or obtain permission to publish this material under the Creative Commons Attribution 4.0 International (CC BY 4.0) License used by PLOS journals. Please closely review the details of PLOS’s copyright requirements here: PLOS Licenses and Copyright. If you need to request permissions from a copyright holder, you may use PLOS's Copyright Content Permission form.

Please respond directly to this email and provide any known details concerning your material's license terms and permissions required for reuse, even if you have not yet obtained copyright permissions or are unsure of your material's copyright compatibility. Once you have responded and addressed all other outstanding technical requirements, you may resubmit your manuscript within Editorial Manager.

Potential Copyright Issues:

1) [Extended Data Figure 1A, B, Fig 4B]: Please confirm whether you drew the images / clip-art within the figure panels by hand. If you did not draw the images, please provide (a) a link to the source of the images or icons and their license / terms of use; or (b) written permission from the copyright holder to publish the images or icons under our CC BY 4.0 license. Alternatively, you may replace the images with open source alternatives. See these open source resources you may use to replace images / clip-art:

- https://commons.wikimedia.org

- https://openclipart.org/"

**Reply to Journal Requirement 7:**

Regarding **Supplementary Figure 3** (**Extended Data Figure 1** in the previous manuscript), the illustration of the human figure was downloaded from the free image website Jitanda (terms available at https://jitanda.com/terms/). Regarding **Figure 4B**, the illustration of the human brain was created using BrainPainter (https://github.com/razvanmarinescu/brain-coloring). All other illustrations were drawn by the first author, Daiki Tatematsu, using Adobe Illustrator. These are described in the **Methods** (page 18, lines 318-320): “The illustration of the human figure in **Supplementary Figure 3** was downloaded from Jitanda (https://jitanda.com/terms/). The illustration of the human brain in **Figure 4** was drawn using BrainPainter (https://github.com/razvanmarinescu/brain-coloring)”.

**General editorial requests:**

(Note: not all will apply to your paper, but please check each item carefully)

* We ask every co-author listed on the manuscript to fill in a contributing author statement, making sure to declare all competing interests. If any of the co-authors have not filled in the statement, we will remind them to do so when the paper is revised. If all statements are not completed in a timely fashion this could hold up the re-review process. If new competing interests are declared later in the revision process, this may also hold up the submission. Should there be a problem getting one of your co-authors to fill in a statement we will be in contact. Please do not add or remove authors without first discussing this with the handling editor. You can see our competing interests policy here: http://journals.plos.org/plosmedicine/s/competing-interests.

All co-authors have declared that they have no competing interests. In addition, we provided the Funding statement on page 48, line 991, where we stated that “The funders had no role in study design, data collection and analysis, decision to publish, or preparation of the manuscript”.

* Please upload any figures associated with your paper as individual TIF or EPS files with 300dpi resolution at resubmission; please read our figure guidelines for more information on our requirements: http://journals.plos.org/plosmedicine/s/figures. While revising your submission, please upload your figure files to the PACE digital diagnostic tool, https://pacev2.apexcovantage.com/. PACE helps ensure that figures meet PLOS requirements. To use PACE, you must first register as a user. Then, login and navigate to the UPLOAD tab, where you will find detailed instructions on how to use the tool. If you encounter any issues or have any questions when using PACE, please email us at PLOSMedicine@plos.org.

After confirming that all figures meet the required specifications using the PACE platform, we provided all figures as individual TIF files, following the PLOS Medicine guidelines.

* Please ensure that the paper adheres to the PLOS Data Availability Policy (see http://journals.plos.org/plosmedicine/s/data-availability), which requires that all data underlying the study's findings be provided in a repository or as Supporting Information. For data residing with a third party, authors are required to provide instructions with contact information (web or email address) for obtaining the data. Please note that a study author cannot be the contact person for the data. PLOS journals do not allow statements supported by "data not shown" or "unpublished results." For such statements, authors must provide supporting data or cite public sources that include it.

We provided the Data availability statement on page 48, lines 960-962 as follows: “The data will be available after a review process by the pn-TTC projects. Please direct requests for data to Department of Neuropsychiatry, The University of Tokyo (https://value.umin.jp/data-resource.html).”

* We expect all researchers with submissions to PLOS in which author-generated code underpins the findings in the manuscript to make all author-generated code available without restrictions upon publication of the work. In cases where code is central to the manuscript, we may require the code to be made available as a condition of publication. Authors are responsible for ensuring that the code is reusable and well documented. Please make any custom code available, either as part of your data deposition or as a supplementary file. Please add a sentence to your data availability statement regarding any code used in the study, e.g. "The code used in the analysis is available from Github [URL] and archived in Zenodo [DOI link]" Please review our guidelines at https://journals.plos.org/plosmedicine/s/materials-software-and-code-sharing and ensure that your code is shared in a way that follows best practice and facilitates reproducibility and reuse. Because Github depositions can be readily changed or deleted, we encourage you to make a permanent DOI'd copy (e.g. in Zenodo) and provide the URL.

The programming code we used to calculate the energy landscape is a Python version of the MATLAB code available from https://github.com/tkEzaki/energy-landscape-analysis. The MATLAB and Python versions share the same functionality. We intend to release the Python version in the near future. A corresponding explanation has been added to the **Methods** (page 13, lines 196–198).

* ETHICS STATEMENT: Please indicate whether consent was waived.

We provided the Ethics statement on page 18, lines 322-329, where we indicated that written informed consent was obtained from the participants.

* FINANCIAL DISCLOSURES: The funding statement should include: specific grant numbers, initials of authors who received each award, URLs to sponsors’ websites. Also, please state whether any sponsors or funders (other than the named authors) played any role in study design, data collection and analysis, the decision to publish, or preparation of the manuscript. If they had no role in the research, include this sentence: “The funders had no role in study design, data collection and analysis, decision to publish, or preparation of the manuscript.”

We provided the Funding statement on page 48, line 991, where we stated that “The funders had no role in study design, data collection and analysis, decision to publish, or preparation of the manuscript”.

* DATA AVAILABILITY STATEMENT: PLOS Medicine requires that the de-identified data underlying the specific results in a published article be made available, without restrictions on access, in a public repository or as Supporting Information at the time of article publication, provided it is legal and ethical to do so. Please see the policy at http://journals.plos.org/plosmedicine/s/data-availability and FAQs at

http://journals.plos.org/plosmedicine/s/data-availability#loc-faqs-for-data-policy

The data in this study will be available after a review process by the pn-TTC projects. We provided the Data availability statement on page 48, lines 960-962 as follows: “The data will be available after a review process by the pn-TTC projects. Please direct requests for data to Department of Neuropsychiatry, The University of Tokyo (https://value.umin.jp/data-resource.html).”

* DATA AVAILABILITY STATEMENT: The Data Availability Statement (DAS) requires revision. For each data source used in your study:

a) If the data are freely or publicly available, note this and state the location of the data: within the paper, in Supporting Information files, or in a public repository (include the DOI or accession number).

b) If the data are owned by a third party but freely available upon request, please note this and state the owner of the data set and contact information for data requests (web or email address). Note that a study author cannot be the contact person for the data.

c) If the data are not freely available, please describe briefly the ethical, legal, or contractual restriction that prevents you from sharing it. Please also include an appropriate contact (web or email address) for inquiries (again, this cannot be a study author).

We provided the Data availability statement on page 48, lines 960-962 as follows: “The data will be available after a review process by the pn-TTC projects. Please direct requests for data to Department of Neuropsychiatry, The University of Tokyo (https://value.umin.jp/data-resource.html).”

FORMATTING - GENERAL

* Abstract: Please structure your abstract using the PLOS Medicine headings (Background, Methods and Findings, Conclusions). Please combine the Methods and Findings sections into one section.

We revised the abstract to follow the PLOS Medicine structure by organizing it into the required sections: Background, Methods and Findings, and Conclusions. Specifically, we combined the Methods and Findings sections into a single section as instructed.

* At this stage, we ask that you include a short, non-technical Author Summary of your research to make findings accessible to a wide audience that includes both scientists and non-scientists. The Author Summary should immediately follow the Abstract in your revised manuscript. This text is subject to editorial change and should be distinct from the scientific abstract. Ideally each sub-heading should contain 2-3 single sentence, concise bullet points containing the most salient points from your study. In the final bullet point of 'What Do These Findings Mean?', please include the main limitations of the study in non-technical language. Please see our author guidelines for more information: https://journals.plos.org/plosmedicine/s/revising-your-manuscript#loc-author-summary.

We added a non-technical Author Summary immediately following the Abstract on pages 5-6, lines 31-58. The summary is written in concise bullet points under the required subheadings, including a clear statement of the study’s limitations in the final bullet point of 'What Do These Findings Mean?'.

* Please express the main results with 95% CIs as well as p values. When reporting p values please report as p<0.001 and where higher as the exact p value p=0.002, for example. Throughout, suggest reporting statistical information as follows to improve clarity for the reader "22% (95% CI [13%,28%]; p</=)". Please be sure to define all numerical values at first use.

We added 95% confidence intervals where appropriate and reported p-values as exact values. We also ensured that all numerical values are clearly defined at first use, following the guidelines.

* Please include page numbers and line numbers in the manuscript file. Use continuous line numbers (do not restart the numbering on each page).

We revised the manuscript to include page numbers and continuous line numbers (not restarting on each page), following the journal’s formatting requirements.

* Please cite the reference numbers in square brackets. Citations should precede punctuation.

We revised the manuscript to ensure that all references are cited in square brackets and placed before punctuation, following the journal’s style guidelines.

FIGURES AND TABLES

* Please provide titles and legends for all figures and tables (including those in Supporting Information files).

We provided clear and complete titles and legends for all figures and tables, including those in the Supporting Information files, following the journal’s guidelines.

* Please define all abbreviations used in each figure/table (including those in Supporting Information files).

We defined all abbreviations used in each figure and table, including those in the Supporting Information files, to ensure clarity and consistency throughout the manuscript. These definitions can be found on pages 48-49, lines 993-1003.

* Please consider avoiding the use of red and green in order to make your figure more accessible to those with color blindness.

We revised the figures to avoid excessive use of red and green, ensuring that the visualizations are accessible to those with color blindness.

SUPPLEMENTARY MATERIAL

* Please note that supplementary material will be posted as supplied by the authors. Therefore, please amend it according to the relevant comments outlined here.

We carefully revised the supplementary materials in full accordance with the provided comments, recognizing that they will be published as supplied.

* Please cite your Supporting Information as outlined here: https://journals.plos.org/plosmedicine/s/supporting-information

We updated the manuscript to ensure that all Supporting Information files are cited appropriately, following the PLOS guidelines.

REFERENCES

* PLOS uses the numbered citation (citation-sequence) method and first six authors, et al.

We formatted all citations according to the PLOS citation style, using numbered references. Additionally, we listed the first six authors followed by et al. for all cited works, following the journal’s guidelines.

* Please ensure that journal name abbreviations match those found in the National Center for Biotechnology Information (NCBI) databases (http://www.ncbi.nlm.nih.gov/nlmcatalog/journals), and are appropriately formatted and capitalised.

We revised all journal name abbreviations to match those listed in the NCBI database and ensured they are properly formatted and capitalized following the journal’s guidelines.

* Where website addresses are cited, please include the complete URL and specify the date of access (e.g. [accessed: 12/06/2023]).

We included complete URLs for all cited websites and have specified the date of access for each, following the journal’s guidelines.

* Please also see https://journals.plos.org/plosmedicine/s/submission-guidelines#loc-references for further details on reference formatting.

We reviewed the reference formatting guidelines and revised our references accordingly to ensure they align with the journal’s requirements.

STUDY TYPE-SPECIFIC REQUESTS

* Abstract: Please include the study design, population and setting, number of participants, years during which the study took place (enrollment and follow up), length of follow up, and main outcome measures.

We updated the **Abstract** to include key methodological and outcome-related details, following the journal’s guidelines. These can be found on pages 3-4, lines 1-30.

* If available, please ensure that the study is reported according to an appropriate guideline, such as STROBE (available from: https://www.equator-network.org/reporting-guidelines/strobe) and include the completed checklist as Supporting Information.

We wrote the manuscript ensuring that the study is reported following the STROBE guidelines. We prepared each section of the manuscript with close attention to the relevant checklist items to promote transparency, reproducibility, and completeness in reporting. We included the completed checklist as Supporting Information (**S1 Checklist**). This is described on page 18, lines 331-335.

* In the manuscript text, please indicate: (1) the specific hypotheses you intended to test, (2) the analytical methods by which you planned to test them, (3) the analyses you actually performed, and (4) when reported analyses differ from those that were planned, transparent explanations for differences that affect the reliability of the study's results. If a reported analysis was performed based on an interesting but unanticipated pattern in the data, please be clear that the analysis was data driven.

In the **Introduction**, we clearly state the hypothesis that adolescents exhibit heterogeneous patterns of psychological distress in response to the COVID-19 pandemic. To test this, we planned to use both conventional statistical methods and energy landscape analysis (ELA) to examine patterns in K6 self-report questionnaire data collected monthly before and during the pandemic. We conducted the analyses as planned, including scoring (total K6 scores) and item-level analysis using ELA. As described on page 8, lines 102-106, ELA was selected to overcome limitations of conventional methods in detecting multidimensional patterns from short-form questionnaires. In addition, we explored associations between depression and brain development using longitudinal MRI data. Some exploratory analyses, such as the linkage between depression and structural brain changes, were data-driven. These have been indicated in the **Discussion** on page 38, lines 663-665, with appropriate discussion of their interpretive limitations.

* Please state in the Methods section whether the study had a prospective protocol or analysis plan. If a prospective analysis plan (from your funding proposal, IRB or other ethics committee submission, study protocol, or other planning document written before analyzing the data) was used in designing the study, please include the relevant document(s) with your revised manuscript as a Supporting Information file to be published alongside your study and cite it in the Methods section. A legend for this file should be included at the end of your manuscript. If no such document exists, please make sure that the Methods section transparently describes when analyses were planned, and when/why any data-driven changes to analyses took place. Changes in the analysis, including those made in response to peer review comments, should be identified as such in the Methods section of the paper, with rationale.

This study is a part of the pn-TTC study, as stated in the **Methods** on page 10, lines 119-122. The study design and data collection procedures were described on page 10, lines 123-131. No major revisions were made to the **Methods** in the current revision, as the analysis plan and procedures remain consistent with those originally described.

* The following list is derived from Geoffrey P Garnett, Simon Cousens, Timothy B Hallett, Richard Steketee, Neff Walker. Mathematical models in the evaluation of health programmes. (2011) Lancet DOI:10.1016/S0140-6736(10)61505-X:

We carefully reviewed the checklist and ensured that our manuscript aligns with the principles and considerations.

* If pertinent, please provide a diagram that shows the model structure, including how the natural history of the disease is represented, the process and determinants of disease acquisition, and how the putative intervention could affect the system.

This recommendation does not apply to our study, as our analysis does not involve modeling the natural history of a disease or simulating the impact of a specific intervention on the system.

* Please provide a complete list of model parameters, including clear and precise descriptions of the meaning of each parameter, together with the values or ranges for each, with justification or the primary source cited and important caveats about the use of these values noted.

We provided a detailed description of particularly important variables and model parameters, including name, symbol, meaning, value, and notes, in the **Supplementary Table 2**. This is described on page 15, lines 240-241.

* Please provide a clear statement about how the model was fitted to the data, including goodness-of-fit measure, the numerical algorithm used, which parameter varied, constraints imposed on parameter values, and starting conditions.

We included a detailed explanation in the **Methods** on pages 13-15, lines 200-241, regarding how the model was fitted to the data. This includes the numerical algorithm used, goodness-of-fit measures, parameters that were estimated, constraints applied to parameter values, and the initial conditions used in the fitting process.

* For uncertainty analyses, please state the sources of uncertainties quantified and not quantified [can include parameter, data, and model structure].

This study did not include an uncertainty analysis.

* Please provide sensitivity analyses to identify which parameter values are most important in the model. Uncertainty estimates seek to derive a range of credible results on the basis of an exploration of the range of reasonable parameter values. The choice of method should be presented and justified.

As a sensitivity analysis for the energy estimate of state 111111 (depressive state), which is the primary focus, we calculated confidence intervals across different time scales (entire period, four periods, and monthly data). This is described on page 24, lines 452-453, and visualized in **Supplementary Fig 10**.

* Please discuss the scientific rationale for the choice of model structure and identify points where this choice could influence conclusions drawn. Please also describe the strength of the scientific basis underlying the key model assumptions.

We selected energy landscape analysis for its ability to capture complex, multidimensional relationships between questionnaire items. As noted on page 24, lines 428-432, we validated our findings using established statistical methods. However, the traditional methods failed to detect item-level differences across periods (**Supplementary Table 4**), highlighting their limitations. In contrast, energy landscape analysis revealed dynamic patterns that conventional approaches could not, supporting the rationale for our model choice.

* For studies that develop a prediction model or evaluate its performance, please ensure that the study is reported according to the TRIPOD statement (https://www.equator-network.org/reporting-guidelines/tripod-statement) and include the completed checklist as Supporting Information. Please add the following statement, or similar, to the Methods: "This study is reported as per the Transparent Reporting of a Multivariable Prediction Model for Individual Prognosis Or Diagnosis (TRIPOD) statement (S1 Checklist)." For studies using machine learning, please use the TRIPOD-AI checklist. When completing the checklist, please use section and paragraph numbers, rather than page numbers.

This study is reported following the Transparent Reporting of a Multivariable Prediction Model for Individual Prognosis Or Diagnosis (TRIPOD-AI) statement. A completed TRIPOD-AI checklist is provided as Supporting Information (**S2 Checklist**). This is described on page 18, lines 331-335.

**Reviewer #1:**

 Important study, but there are some major points to clarify the study.

**Reply to Reviewer summary:**

We thank the reviewer for recognizing the value of our work and for the constructive comments that significantly improved the quality of our study. We have prepared a revised version of the manuscript that addresses all of the comments raised.

1.Abstract
Please indicate the number of samples (total and important subgroups), and K6 cut-offs.

**Reply to comment 1:**

As suggested by the reviewer, we summarized the number of samples categorized by the total K6 scores (cutoffs: 5, 8, 13) in **Supplementary Table 5** and also described them in the Abstract on page 3, lines 18-22:

*"We also identified two groups of students with different K6 dynamics and energy landscapes. The first group consisted of 61 participants whose total K6 score was relatively low (less than 5) and stable over time, and the second group consisted of 23 participants whose total K6 score was higher (with most being higher than 5) and less stable."*

and on page 28, lines 490-492. The cutoff values of 5, 8, and 13 were chosen based on [1] (see **References (for Reviewer #1)** of this document). In addition, we also summarized the whole study as a flowchart in **Supplementary Fig 1**. We believe that these changes have made the text clearer and easier to understand.

2. Throughout the study K6 cut-offs can significantly change the interpretation of the results of this analysis. Please clearly mention how cut-offs of K6 (5, 8 or 13) are used to determine the severity of the distresses in this study.

**Reply to comment 2:**

In this study, we binarized each item of K6 separately using its mean value. As a sensitivity analysis, we added the results showing the effects of different cutoff values (median, threshold of 1 or 0.833) in **Supplementary Fig 12** and discussed them in **Supplementary Note 1**. The cutoff values of 1 or 0.833 (=5/6) were chosen because (a) the mean value of each item of K6 was less than 1, so it was 1 when rounded to an integer; (b) we considered the clinical cutoff value of 5 [1] in the total K6 score.

As suggested by the reviewer, we also summarized the data based on different cutoff values (5, 8, 13) of the total K6 scores (**Supplementary Table 5**). See also **Reply to comment 1**.

3. In relation to the above cut-off value of K6, it is difficult to understand the explanation of G1 and G2. Please explain carefully what is meant by higher K6 or low K6.

**Reply to comment 3:**

Rather than using a specific cutoff value to classify individuals, we let a data-driven method (*k*-means clustering) decide the grouping. The method looked at the time course of each participant's total K6 score and determined which time courses were closer than others. This resulted in two groups G1 and G2. As shown in **Fig 3AB**, when the two groups were compared, G1 had relatively lower K6 scores and G2 had relatively higher K6 scores. Specifically, G1 consistently showed low scores (less than 5), whereas the majority of G2 had total scores exceeding 5 (**Supplementary Table 5** and **Supplementary Fig 5**). This clustering therefore has clinical relevance.

4. Discussion

It is difficult to understand the overall picture of what happened to what percentage of people (total and subgroups) in this analysis. Therefore, it is not clear how the results can be applied to future pandemics by what degree. There are some major limitations in applying this study to all of Japan and the world as a whole, but we hope that you will take this into consideration and improve this aspect in the text.

**Reply to comment 4:**

Thank you very much for highlighting areas that might be unclear to readers. As suggested, we created a new figure summarizing the whole study as a flowchart (**Supplementary Fig 1**), which we are sure will help readers in understanding the overall picture. Please note that the timeline of K6 responses for all participants is shown in **Fig 1A**, and the timeline of all the other measurements is summarized in **Fig 4A**. We also summarized the number of people in each subgroup in **Supplementary Table 5**.

Our results showed an unexpected degree of heterogeneity in psychological states and their transition dynamics, which we can take as a lesson for future pandemics: we need to be vigilant about the possible adverse effects on adolescents and provide psychological support to those who need it. We discussed this point on pages 37-38, lines 642-645.

Regarding the limitations raised by the reviewer, we agree that our cohort consisted of high school students living in the capital city of Japan, Tokyo, and that the generalizability of our findings to other areas and countries needs to be confirmed using the same method. We leave this for future studies. We discussed this point on page 38, lines 646-650.

**References (for Reviewer #1)**

1. Furukawa TA, Kawakami N, Saitoh M, Ono Y, Nakane Y, Nakamura Y, et al. The performance of the Japanese version of the K6 and K10 in the World Mental Health Survey Japan. Int J Methods Psychiatr Res. 2008;17(3):152-8. doi: 10.1002/mpr.257. PubMed PMID: 18763695; PubMed Central PMCID: PMCPMC6878390.

**Reviewer #2:**

**Reply to Reviewer summary:**

We thank the reviewer for the constructive comments that significantly improved the quality of our study. We have prepared a revised version of the manuscript that addresses all of the comments raised.

1)      The manuscript uses the Ising model in physics as the basis for the energy landscape analysis, which assigns energies to states representing responses to the K6 items. However, there is no detailed explanation about why the Ising model was selected over alternative models in the context of psychological data.

**Reply to comment 1:**

The energy landscape is a concept that has attracted attention in neuroscience and other fields in recent years [1-4] (see **References (for Reviewer #2)**), and we demonstrated in this paper that it is also applicable to psychological and psychiatric data. The Ising model is a natural way to estimate the energy landscape from available data with minimal assumptions (i.e., maximum entropy), and indeed we validated the accuracy of the data fit to the model in **Supplementary Fig 4**. See **Reply to comment 3** regarding the use of alternative models.

2)      Binarization of Data: The conversion of continuous K6 scores into binary data (above or below the mean) may lead to a loss of information. The authors should discuss the limitations of binarizing continuous data and its impact on the sensitivity of the results. Additionally, exploring how different thresholds (such as median or clinically relevant cut-offs) impact the energy landscape would be valuable.

**Reply to comment 2:**

We agree with the reviewer that binarizing the score potentially limits the resolution of possible psychological states. We mentioned the limitation of binarization in the **Discussion** (page 38, lines 660-663) as follows:

*"In our analysis, this [binarization] simplified and emphasized the contrast between healthy and depressive states, but it is possible that removing the binarization may reveal other psychological states. Improving the limitation will be a matter for the future."*

Furthermore, as suggested by the reviewer, we added the results showing the effect of different cutoff values (median, threshold of 1 or 0.833) on the energy landscape in **Supplementary Figure 12** and discussed them in **Supplementary Note 1**. The cutoff values of 1 or 0.833 (=5/6) were chosen because: (a) the mean value of each item of K6 was less than 1, so it was 1 when rounded to an integer; (b) we considered the clinical cutoff value of 5 [5] in the total K6 score. The results were mostly similar to the mean cutoffs.

For comparison, we also summarized the data based on clinically relevant cutoff values (5, 8, 13) of the total K6 scores (**Supplementary Table 5**). The cutoff values of 5, 8, and 13 were chosen based on [5]. The result suggests that the total scores alone were not a sensitive indicator of change in psychological distress in our dataset.

3)      The manuscript mentions the use of two accuracy indices—entropy and Kullback-Leibler divergence—to validate the pairwise maximum entropy model. These are appropriate choices, but it would be useful to include a more in-depth comparison of the energy landscape model with traditional statistical models used in psychology to demonstrate its added value.

**Reply to comment 3:**

Our analysis was validated by traditional statistical analysis using the total K6 score (**Fig 2E**, Jonckheere-Terpstra trend test). However, the total K6 score alone does not reveal which combination of items contributed to the healthy and depressive states, nor how these states changed over time. By viewing the dynamic fluctuation of item-level scores as energy landscapes, we were able to identify "healthy" and "depressive" states as two stable states, and describe their changes between before and after the pandemic. We mentioned this on page 23, lines 419-422.

For comparison, when the K6 scores were analyzed item by item, traditional statistical methods such as the Jonckheere-Terpstra trend test, Tukey's test, or Mood's median test did not reveal significant differences among the different periods (see **Supplementary Table 4**), suggesting that these methods were not sensitive enough to detect item-level differences. We mentioned this on page 24, lines 428-432.

4)      The authors acknowledge that constructing energy landscapes based on monthly data may lead to lower accuracy due to a small sample size. Including statistical diagnostics for this potential limitation, such as confidence intervals around the energy estimates, would enhance the reader's understanding of the robustness of the findings.

**Reply to comment 4:**

We added confidence intervals around the energy estimates of state 111111 (depressive state) for the entire period, for the four periods, and for the monthly data in **Supplementary Fig 10**. We see that the monthly data have wider confidence intervals, as we expected. We described this on page 24, lines 452-453.

5)      The manuscript uses Gibbs sampling to simulate transitions between the depressive (111111) and healthy (000000) states. While the results are clear, the assumptions behind the transition process are not fully explained. For instance, does the energy landscape assume Markovian properties, and if so, how is the memoryless nature of transitions justified for psychological data, which may show temporal dependence?

**Reply to comment 5:**

While the Ising model used to derive the energy landscape does not assume the Markovian property, this transition simulation does so based on the detailed balance assumption [6]. Extending the model to cases where this simplifying assumption does not hold (e.g., kinetic Ising model) requires more data for fitting and will be left to future studies. We mentioned this on page 24, lines 434-435.

We agree with the reviewer that our data may show temporal dependence. In this respect, the clustering of the participants into two groups (G1 and G2) and running simulations in each group was informative (**Fig 3I-L**), reflecting the fact that different individuals have different temporal characteristics.

Note that we also calculated the monthly transition probabilities between two basins (**Supplementary Fig 8BEF** and **Supplementary Note 3**) and were pleased to confirm tendencies similar to those observed in the simulation. Specifically, the pandemic prompted a shift from the depressive basin to the healthy basin.

6)      In some sections, the median time for transitions between states is compared across periods. A more statistical comparison between transition times (e.g., confidence intervals, hypothesis testing, or bootstrapping) would provide more rigor to these comparisons.

**Reply to comment 6:**

As suggested, we made a statistical comparison across different periods and updated the figures (**Fig 2GH**, **Fig 3I-L**) and the main text (page 24, lines 439-442; page 29, lines 520-525).

7)      The manuscript stratifies participants into two groups (G1 and G2) using k-means clustering based on their K6 scores. While k-means clustering is widely used, it might not always be the most appropriate method for clustering time-series data. The use of time-series-specific clustering methods (such as dynamic time warping) could be explored. At a minimum, the authors should discuss why k-means was chosen and how sensitive the results are to the number of clusters.

**Reply to comment 7:**

We used k-means clustering here because of its computational efficiency for large datasets. As a sensitivity analysis, as suggested by the reviewer, we performed clustering via dynamic time warping and obtained essentially the same clustering except for four individuals as shown in **Supplementary Fig 14**. This demonstrates the robustness of our clustering. We believe that the k-means clustering is simpler and more interpretable.

8)      The group-level comparisons could be expanded with additional statistical tests, such as ANOVA or mixed-effects models, to provide more robust insights into the differences between the groups in terms of their psychological distress and brain morphology.

**Reply to comment 8:**

We indeed used general linear mixed models (GLMMs) to compare the cortical thickness of the brain between the two groups G1 and G2 (**Supplementary Tables 7** and **8**). This is described on page 33, lines 553-556.

9)      The mathematical equations for the maximum entropy model and the computation of probabilities using the energy function are clearly explained. However, readers who are not familiar with the Ising model might struggle with the concepts. It would be helpful if the authors provided an intuitive example or additional explanation of how the model handles interactions between different psychological items (e.g., K6-1 to K6-6).

**Reply to comment 9:**

As suggested by the reviewer, we added an explanation of how the Ising model handles interactions between different items (page 19, lines 353-358).

10)     The inclusion of pairwise interaction terms (Jij) between items in the model is briefly mentioned, but the implications of these interactions should be discussed in more detail. Are the interactions assumed to be symmetric, and how are they estimated from the data?

**Reply to comment 10:**

As the reviewer correctly pointed out, Jij was assumed to be symmetric (Jij = Jji). We described this on page 14, line 222. All coefficients of Jij for the entire period were positive (**Supplementary Table 3**), meaning that states become more stable when different items take the same values. This is consistent with positive correlations between the items (**Fig 1C**). We added this discussion on page 22, lines 387-389. We also described the maximum likelihood estimation of the coefficients in the **Methods** (pages 13-15, lines 200-241).

11)     As noted earlier, the binarization process could significantly impact the model outcomes. A sensitivity analysis exploring how the results vary when different thresholds are used (e.g., different cut-offs for binarization or alternative scoring methods) would improve the robustness of the findings.

**Reply to comment 11:**

As suggested by the reviewer, we added the results of binarization by different cutoff values (median, threshold of 1 or 0.833) in **Supplementary Fig 12** and discussed them in **Supplementary Note 1**. The results were mostly similar to the mean cutoffs. See also **Reply to comment 2**.

12)     Although the energy landscape analysis is the focus of the manuscript, comparing its performance to more traditional statistical models (e.g., latent class analysis or longitudinal mixed-effects models) would provide context for the added value of ELA.

**Reply to comment 12:**

See **Reply to comment 3** regarding the comparison with traditional statistical models.

13)     The manuscript links psychological distress profiles (from energy landscape analysis) with changes in cortical thickness. This is an interesting application, but more details are needed on how these associations were tested. Were the brain data adjusted for potential confounders such as age, sex, and socioeconomic status? Additionally, multiple comparison correction methods should be clearly stated given the number of brain regions analysed.

**Reply to comment 13:**

We indeed used the false discovery rate (FDR) to correct for multiple comparisons. This is described on pages 33-34, lines 558, 561-563, 572-574, where *q* values are mentioned. We clarified this in all places.

14)     Longitudinal studies often face challenges with missing data or participant dropout over time. The manuscript should discuss how missing data were handled, especially since the data collection spanned over two years. If imputation methods were used, details about the method and its justification are essential. Alternatively, a discussion on how the missing data could have impacted the results (e.g., selection bias or reduced statistical power) should be included.

**Reply to comment 14:**

The missing data were interpolated by Gaussian process regression. This method incorporates available data of the same individual (**Fig 1A**) to provide the best estimate for the missing data (**Supplementary Fig 2**) in an unbiased manner. We adjusted the kernel function to faithfully reflect the rapid fluctuation of K6 scores. For comparison, we also performed interpolation by spline curves (**Supplementary Fig 13A**). The values obtained were mostly similar to those obtained by Gaussian process regression (**Supplementary Fig 13B**) and did not affect the binarization results (**Supplementary Fig 13C**). We described this in **Supplementary Note 1**.

15)     While the manuscript mentions the use of Gaussian process regression to smooth time-series data, it would be beneficial to explain why this approach was chosen over others (such as Kalman filtering or spline-based methods) for handling irregular time points. Additionally, clarifying how the temporal aspect of psychological distress is accounted for in the energy landscape framework would be helpful, as this is crucial in longitudinal data.

**Reply to comment 15:**

As shown in **Supplementary Fig 2**, K6 scores fluctuated rapidly for each individual. In order to faithfully reflect the fluctuations, we opted for rugged, rather than smooth, interpolation. Gaussian process regression makes this possible by adjusting the kernel function. We also performed smoothing by spline curves (**Supplementary Fig 13A**), as suggested by the reviewer. The values obtained were mostly similar to those obtained by Gaussian process regression (**Supplementary Fig 13B**) and did not affect the binarization results (**Supplementary Fig 13C**). We described this in **Supplementary Note 1**.

In this paper, we addressed the temporal aspect of psychological distress by dividing the dataset into four Periods 1-4, and comparing the results between different Periods. We added the explanation in the **Discussion** (page 36, lines 596-598). We also performed monthly analysis of energy landscapes (**Supplementary Fig 9ABC**), which suggests that students were less depressed when school days per week were fewer (**Supplementary Fig 9C**). This monthly analysis also confirms the same tendency, although we note that the accuracy of landscape construction may have been lower due to a small sample size, as seen in the wider confidence intervals of the energy estimates (**Supplementary Fig 10**).

16)     The manuscript presents multiple complex statistical outputs, including energy landscapes, transition times, and brain structure associations. While each result is explained in isolation, the overall narrative could be improved by integrating these findings more clearly. For instance, the discussion could benefit from a summary that ties together how the psychological states (as modelled by the energy landscape) correlate with brain structural changes and how these dynamics evolve throughout the pandemic.

**Reply to comment 16:**

As suggested by the reviewer, we added an explanation in the **Discussion** to summarize energy landscapes, transition times, and brain structure associations (page 37, lines 617-627).

17)     The study infers that the COVID-19 pandemic and the associated states of emergency impacted psychological states and brain development. However, the ability to make causal claims from the energy landscape model, which is fundamentally descriptive, should be carefully nuanced. The manuscript should acknowledge that causal interpretations are limited unless supported by further experimental or interventional data.

**Reply to comment 17:**

As suggested by the reviewer, we added in the **Discussion** that "the association between psychological states and brain development remains descriptive, and further experimental or interventional studies are needed to validate causality" (page 38, lines 663-665).

18)     In sections where multiple brain regions or psychological states are being analysed, it is crucial to ensure that appropriate corrections for multiple comparisons (such as Bonferroni correction or False Discovery Rate) are applied. While the manuscript references some corrections, it would be beneficial to explicitly describe which corrections were applied and to what analyses.

**Reply to comment 18:**

As we stated in the **Methods** (page 17, lines 309-310), we used the false discovery rate (FDR) to correct for multiple comparisons. This applies to pages 33-34, lines 558, 561-563, 572-574, where *q* values are mentioned. We clarified this in all places.

19)     There is no mention of power calculations in the manuscript. Given the relatively small sample size (84 participants), especially when analysing brain imaging data, a power analysis would help to assess whether the study had sufficient power to detect meaningful differences in psychological distress or brain structural changes. This is particularly relevant for detecting subtle effects in longitudinal psychological and brain measures.

**Reply to comment 19:**

As suggested by the reviewer, we performed a power calculation and described it in **Supplementary Note 4** and **Supplementary Fig 15**. The result suggests that the model for cortical thickness in the caudal middle prefrontal cortex had sufficient sensitivity in the dataset analyzed, but the model in the temporal pole needs to be confirmed with a larger sample size. Testing with more than 220 participants may provide sufficient power (> 80%) for the latter model.

20)     The binarization process simplifies continuous psychological distress scores into "above or below average" categories. This simplification may lead to the loss of important granularity. The discussion should elaborate on what is lost or gained by this binning approach, and whether any important psychological nuances are potentially missed by treating all individuals above or below the threshold as homogeneous groups.

**Reply to comment 20:**

We mentioned the limitation of binarization in the **Discussion** (page 38, lines 660-663). For comparison, we performed sensitivity analyses using different cutoffs in **Supplementary Fig 12** and discussed them in **Supplementary Note 1**. The results were mostly similar to the mean cutoffs. Details are described in **Reply to comment 2**.

21)     The energy landscape model treats each state (combination of K6 scores) as either "healthy" or "depressive," but this binary treatment of psychological distress might overlook intermediary states of mental health. Providing more interpretation of what the middle ground looks like (e.g., scores that are neither fully healthy nor fully depressive) would help bridge the gap between the model and clinical reality.

**Reply to comment 21:**

We agree with the reviewer that there could be states other than "healthy" and "depressive". Indeed, we showed such states (010101 or 011110) in disconnectivity graphs (**Fig 2F**), but they had much higher energy than the healthy state 000000 (with a difference > 3), i.e., they had much lower probability (more than 20-fold). Moreover, the graph of the basin of attraction (**Fig 2C** and **Fig 3CD**) suggests that the two states ("healthy" and "depressive") are dominant. We mentioned this on page 23, lines 424-425.

Regarding the "middle ground" mentioned by the reviewer, we observed that some participants in G2 maintained a total K6 score above 5 across all periods, while others had a total K6 score below 5 in at least one period (**Supplementary Fig 5**). We mentioned this on page 27, lines 488-490. In the language of "healthy" and "depressive" basins, the former remained in depressive basins across all periods, whereas the latter alternated between the healthy and depressive basins. It is therefore possible to interpret the latter phenotype as being less severe than the former, and thus as the "middle ground" between fully healthy and fully depressed.

22)     The novel nature of the energy landscape analysis approach may make it harder for other researchers to replicate. The manuscript should include clear instructions or supplementary material with code to allow others to reproduce the results. If any custom software or scripts were used for the energy landscape modelling or Gibbs sampling simulations, these should be shared in a public repository.

**Reply to comment 22:**

The programming code we used to calculate the energy landscape is a Python version of the MATLAB code available from [7] along with a tutorial. This MATLAB code has the same functionality as our Python code. We also plan to release our Python code in the near future. We added the corresponding description in the **Methods** (page 13, lines 196-198).

23)     It is important to report diagnostic checks for the energy landscape models. For example, did the model converge properly? Were any diagnostics conducted to ensure that the model fits the data appropriately? Reporting these checks would enhance the reader's confidence in the robustness of the analysis.

**Reply to comment 23:**

The accuracy of data fitting to the model is shown in **Supplementary Fig 4**. For all possible states ($2^{6}=64$ states), the probabilities calculated from the data $P_{\text{data}}$ and from the model $P_{\text{model}}$ are plotted as black dots. The black dots are approximately on the line $y=x$, indicating a good fit.

24)     Beyond the two clusters identified (G1 and G2), it might be worth exploring whether other demographic variables (e.g., gender, socioeconomic status, academic performance) could serve as additional or alternative stratification factors. Some subgroups may have experienced different psychological impacts during the pandemic, and the current stratification based purely on K6 scores might overlook these effects.

**Reply to comment 24:**

We compared demographic variables between G1 and G2 and describe the results in **Supplementary Table 6**. As noted in the main text, there were significantly more females in G2, and there were also differences in the GHQ scores. In contrast, no significant differences were observed in IQ or parental socioeconomic status. We mentioned this on page 28, lines 497-498.

**References (for Reviewer #2)**

1. Ezaki T, Watanabe T, Ohzeki M, Masuda N. Energy landscape analysis of neuroimaging data. Philos Trans A Math Phys Eng Sci. 2017;375(2096). doi: 10.1098/rsta.2016.0287. PubMed PMID: 28507232; PubMed Central PMCID: PMCPMC5434078.
2. Ezaki T, Fonseca Dos Reis E, Watanabe T, Sakaki M, Masuda N. Closer to critical resting-state neural dynamics in individuals with higher fluid intelligence. Commun Biol. 2020;3(1):52. Epub 20200203. doi: 10.1038/s42003-020-0774-y. PubMed PMID: 32015402; PubMed Central PMCID: PMCPMC6997374.
3. Watanabe T, Masuda N, Megumi F, Kanai R, Rees G. Energy landscape and dynamics of brain activity during human bistable perception. Nat Commun. 2014;5:4765. Epub 20140828. doi: 10.1038/ncomms5765. PubMed PMID: 25163855; PubMed Central PMCID: PMCPMC4174295.
4. Watanabe T, Hirose S, Wada H, Imai Y, Machida T, Shirouzu I, et al. Energy landscapes of resting-state brain networks. Front Neuroinform. 2014;8:12. Epub 20140225. doi: 10.3389/fninf.2014.00012. PubMed PMID: 24611044; PubMed Central PMCID: PMCPMC3933812.
5. Furukawa TA, Kawakami N, Saitoh M, Ono Y, Nakane Y, Nakamura Y, et al. The performance of the Japanese version of the K6 and K10 in the World Mental Health Survey Japan. Int J Methods Psychiatr Res. 2008;17(3):152-8. doi: 10.1002/mpr.257. PubMed PMID: 18763695; PubMed Central PMCID: PMCPMC6878390.
6. Masuda N, Islam S, Thu Aung S, Watanabe T. Energy landscape analysis based on the Ising model: Tutorial review. PLOS Complex Systems. 2025;2(5):e0000039 %@ 2837-8830.

7.	Ezaki T. Energy Landscape Analysis Toolbox (ELAT) for MATLAB [cited: May 1 2025]. Available from: <https://github.com/tkEzaki/energy-landscape-analysis>.
