# Supplementary material for: Psychological distress among Japanese high school students during the COVID-19 pandemic: An energy landscape analysis
Source: PLoS Med. 2026 Jan 22;23(1):e1004884. doi: 10.1371/journal.pmed.1004884 (PMC12826503; doi:10.1371/journal.pmed.1004884)
Supplement: Attachment — Submitted filename: PBP_revision2.docx [file pmed.1004884.s040.docx]

**Response to editor and reviewer comments**

Daiki Tatematsu, Naotoshi Nakamura, Masato Abe, Tetsuo Ishikawa, Takahiro Ezaki, Lin Cai, Eiryo Kawakami, Kazuyuki Aihara, Atsushi Nishida, Naohiro Okada, Naoki Masuda, Kiyoto Kasai, Shinsuke Koike, Shingo Iwami

November 27, 2025

We would like to thank the reviewers for their thorough reading of the manuscript and their helpful comments and suggestions. In this document, we present the reviewers’ comments in black and provide our responses in red. All changes made in response to the reviewers’ comments in PMEDICINE-D-24-03134R3 have been incorporated in the revised version of the manuscript. We believe that the manuscript has been considerably improved by these changes. All co-authors have agreed with these changes.

**Reviewer #1:**

I have carefully reviewed the response letter and the revised manuscript. It is clear that the authors have appropriately and thoroughly addressed the reviewer comments. The revisions reflect a sincere effort to enhance clarity, methodological transparency, and accessibility of the energy landscape analysis. The additional explanations, sensitivity analyses, and improved figure annotations contribute meaningfully to the overall quality and reproducibility of the work. I believe the authors have responded in good faith to all points raised, and no major concerns remain unaddressed.

**Reply to Reviewer #1:**

We sincerely appreciate your careful review and thoughtful assessment of our revised manuscript. We are very pleased to hear that the revisions have addressed the reviewers’ comments satisfactorily. Thank you for recognizing our efforts to improve the clarity and reproducibility of the work.

**Reviewer #2:**

I appreciate the authors' thorough revisions and the clear effort made to address my previous comments. The manuscript is substantially improved. I would like to offer a few further suggestions to enhance the statistical and methodological robustness of the analysis.

**Reply to Reviewer #2:**

We sincerely appreciate the reviewer’s positive assessment of our revisions and the acknowledgment that the manuscript has substantially improved. We are grateful for the additional suggestions aimed at further enhancing the statistical and methodological rigor of our analysis. We have carefully considered and incorporated these recommendations as detailed below.

a)	While confidence intervals around energy estimates are provided for selected states, consider extending uncertainty quantification to all estimated parameters of the Ising model (e.g., hih_ihi, JijJ_{ij}Jij) to allow a more comprehensive assessment of robustness, especially across time periods.

**Reply to comment a:**

We calculated confidence intervals for the estimated parameters. As described in the revised manuscript, we added the following statement on page 22, lines 411-413: “Furthermore, to quantify the uncertainty of the estimated parameters, we computed confidence intervals for both $h_{i}$ and $J_{ij}$ and confirmed that these intervals were notably narrow, indicating stable estimates”. In addition, we created **Supplementary Figure 7** to visualize these confidence intervals.

“

b)	Although the manuscript mentions validation via entropy and KL divergence, it would be helpful to include additional information on model convergence diagnostics (e.g., likelihood convergence plots, changes in parameter estimates over iterations) to support the reliability of the energy landscape estimates.

**Reply to comment b:**

We plotted the convergence of estimation errors and the variation in parameter estimates across iterations (**Supplementary Figure 8**). Accordingly, we added the following statement to the revised manuscript on page 22, lines 413-415: “We also confirmed stable parameter estimation based on the convergence of estimation errors and the change in parameter estimates across iterations”.

c)	While the authors acknowledged the Markovian assumption and its limitations, exploring even a basic extension to non-Markovian transitions (e.g., via lagged variables or conditional entropy) could improve the model's realism for longitudinal psychological data. At minimum, this should be acknowledged as a potential future improvement in the Discussion.

**Reply to comment c:**

We revised the Discussion on page 39, lines 719-724, to acknowledge this point as a potential future direction: “Furthermore, while the current energy landscape analysis relies on the Markovian assumption, future methodological extensions could relax this constraint to better capture temporal dependencies inherent in longitudinal psychological processes. For example, adopting a kinetic Ising-based formulation or incorporating lagged variables may enable the model to represent non-Markovian dynamics and memory effects more realistically.”

d)	The current model is descriptive, and while it shows intuitive trends, some form of cross-validation (e.g., time-based splitting) or out-of-sample predictive accuracy (for transitions or group classification) could help establish its generalisability and utility in future applications.

**Reply to comment d:**

We added the following statement to the Discussion on page 39, lines 733-735: “Future studies should test the model’s generalizability by applying the energy landscape analysis to other cohorts, thereby evaluating its predictive validity and practical relevance across diverse populations.”

e)	While the energy landscape analysis has been contrasted descriptively with traditional methods, a quantitative comparison of predictive performance (e.g., using AIC/BIC, likelihood ratios, or classification metrics) between ELA and alternative models (like mixed-effects models or LCA) could better establish its empirical advantage.

**Reply to comment e:**

In response to this comment, we conducted an additional quantitative analysis using latent class analysis (LCA) to validate further the findings obtained from the energy landscape analysis (ELA). The purpose of this analysis was to determine whether an alternative modeling approach could reproduce the temporal patterns identified by the ELA, thereby strengthening the empirical robustness of our results.

To address this, based on the total K6 scores, we estimated both 2-class and 3-class LCA models (**Supplementary Figure 11A**). The resulting classes aligned well with clinically interpretable levels of psychological distress:

- 2-class solution:

Class 1: healthy (scores near zero)

Class 2: depressive (higher scores)

- 3-class solution:

Class 1: healthy (scores near zero)

Class 2: moderately depressive (intermediate scores)

Class 3: highly depressive (higher scores)

**Supplementary Figure 11A |** Participant-level plots showing classes of datapoints based on latent class analysis with 2- and 3-class solutions.

To determine whether the temporal patterns inferred from ELA were reproduced by LCA, we compared the proportion of datapoints belonging to depressive classes across the 4 periods (**Supplementary Figure 11B**).

- In both the 2-class and 3-class solutions, Period 1 (pre-pandemic) showed the highest proportion of datapoints belonging to depressive classes.
- In contrast, the proportions of datapoints belonging to depressive classes were lower in Periods 2-4 (during the pandemic).

This pattern is consistent with the results of the ELA, which showed that the energy of the depressive state (111111), indicating the difficulty of being in that state, was substantially higher in Periods 2-4 than in Period 1. Thus, the LCA independently reproduced the key temporal shift identified by the ELA, further supporting the robustness of the ELA findings.

**Supplementary Figure 11B |** Count (proportion) of datapoints in each class identified by latent class analysis across the 4 periods for 2- and 3-class solutions.

To further evaluate the quantitative agreement between the ELA and LCA results, we examined the relationship between the energy of the depressive state (111111) estimated by the ELA and the proportion of datapoints assigned to depressive classes in the LCA for these 4 periods (**Supplementary Figure 11C**). We found a strong negative correlation:

- 2-class solution: r = –0.95, p = 0.047
- 3-class solution: r = –0.96, p = 0.043

Because higher energy indicates a lower likelihood of being in that state, this negative correlation shows that the proportion of depressive data points identified by LCA is positively associated with the frequency of the depressive state (111111) estimated by the ELA, confirming strong agreement between the two methods.

**Supplementary Figure 11C |** Relationship between the energy of 111111 state estimated by energy landscape analysis and the count (proportion) of data points in each class identified by latent class analysis. Each point represents one of the 4 periods. 111111 (binarized responses of 1 to all six items) represents the “depressive” state.

Overall, these results demonstrate that LCA, a traditional clustering approach, independently reproduced the key pandemic-related temporal shift identified by the ELA, thereby strengthening the validity and robustness of the ELA findings. A description of this validation analysis has been added to **Supplementary Note 3** of the revised manuscript, and **Supplementary Figure 11** has been prepared to illustrate the results. In addition, the corresponding explanation has been added to the revised manuscript on page 24, lines 458-460.

f)	While it is appreciated that the Python version of the modelling code is based on an existing MATLAB repository, I strongly recommend making the actual Python scripts used in this study available (e.g., via GitHub and archived on Zenodo) at the time of publication to meet reproducibility standards.

**Reply to comment f:**

We made the Python scripts used in this study publicly available on GitHub (https://github.com/ttttmmttddiikk/Energy_Landscape_Analysis) and archived on Zenodo (DOI: 10.5281/zenodo.17585043) to ensure reproducibility. We added the following statement on page 13, lines 215-217: “The Python code is available on GitHub (https://github.com/ttttmmttddiikk/Energy_Landscape_Analysis) and Zenodo (DOI: 10.5281/zenodo.17585043).”

g)	The Ising model's structure implies inter-item associations, yet their clinical interpretation remains underdeveloped. I suggest including a brief discussion of how these item-level interactions (e.g., between specific K6 items) might inform mental health screening or interventions in school settings.

**Reply to comment g:**

We added the following statement to the Discussion on pages 37-38, lines 687-693: ”For example, examining inter-item associations of K6 identified by the energy landscape analysis, beyond the total K6 score, could provide deeper insights into how specific symptoms, such as concentration difficulties, fatigue, or feelings of hopelessness, interact and evolve over time. These item-level patterns may highlight distinct psychological profiles that are overlooked by aggregate scoring, helping educators and school health professionals to identify at-risk students and tailor preventive support more effectively during future public health crises.”

h)	Given the authors' novel use of energy landscapes, it may be helpful to briefly discuss the potential for integrating such models into economic evaluations or predictive modelling frameworks (e.g., for assessing cost-effectiveness of school-based interventions under pandemic scenarios). This could broaden the relevance of the approach.

**Reply to comment h:**

We added the following statement to the Discussion on page 38, lines 696-702: “Furthermore, in addition to characterizing psychological dynamics, the energy landscape analysis could be integrated into economic and predictive modelling frameworks to quantify the potential value of early mental health interventions. For instance, by linking energy landscape-derived stability metrics with longitudinal outcomes such as absenteeism, academic performance, or clinical referrals, future studies could estimate the cost-effectiveness of targeted school-based prevention under different resource or pandemic scenarios. This integration may ultimately inform policy decisions on allocating resources for youth mental health programs.”

**Editors Requests:**

We would like to emphasize point e), made by the statistical reviewer, who noted that a quantitative comparison of the predictive performance of ELA and alternative models could better demonstrate its empirical advantage. We strongly agree and encourage you to include the suggested analysis.

We appreciate the statistical reviewer’s suggestion regarding point (e) and fully agree that a quantitative comparison with alternative models would strengthen the empirical validity of the ELA findings. Therefore, we conducted an additional latent class analysis (LCA) to quantitatively validate the ELA results. The findings confirmed consistency between the two approaches, supporting the robustness of the ELA. The descriptions were added on page 24, lines 458-460 of the revised manuscript, and the results are presented in **Supplementary Note 3** and **Supplementary Figure 11**. Please see our detailed reply to **Reply to comment e**.

GENERAL

* Please confirm that your title complies with to PLOS Medicine's style. Your title must be nondeclarative and not a question. It should begin with main concept if possible. "Effect of" should be used only if causality can be inferred, i.e., for an RCT. Please place the study design ("A randomized controlled trial," "A retrospective study," "A modelling study," etc.) in the subtitle (i.e., after a colon).

We reviewed the title and confirmed that it complies with PLOS Medicine’s title format requirements.

* Statistical reporting: Please revise throughout the manuscript, including tables and figures.

We rechecked the statistical reporting throughout the manuscript, including tables and figures.

- Please report statistical information as follows to improve clarity for the reader ""XX (95% CI [XX,XX]; p</=)"".

We revised the statistical reporting to follow the suggested format where applicable.

- Please separate upper and lower bounds with commas instead of hyphens as the latter can be confused with reporting of negative values.

We revised the notation to separate the upper and lower bounds with commas as requested.

- Please repeat statistical definitions (HR, CI etc.) for each set of parentheses.

We revised the manuscript to repeat the statistical definitions for each set of parentheses as requested.

* Please ensure that all abbreviations are defined at first use throughout the text (including statistical abbreviations). Please also check figures and tables.

We checked that all abbreviations are defined at first use throughout the text, figures, and tables.

* Please ensure that tables and figures, including those in supplementary files, are appropriately referenced in the main text.

We ensured that all tables and figures, including those in the supplementary materials, are appropriately referenced in the main text.

* Please check that any use of statistical terms (such as trend or significant) are supported by the data, and if not please remove them.

We checked that all uses of statistical terms are supported by the data.

* Please ensure that where relevant figures include 95% CIs.

We included 95% confidence intervals in the relevant figures where applicable.

* Please review your text for claims of novelty or primacy (e.g. 'for the first time') and remove this language.

We reviewed the manuscript and removed any claims of novelty or primacy.

* Data availability: “The data will be available after a review process by the pn-TTC projects” – Does this mean that researchers interested in the data must submit a proposal via the email address you provided, which will then be reviewed by pn-TTC projects? Could you explain why the data is not publicly available?

As the dataset contains sensitive personal information, it cannot be made publicly available. To clarify this point, we have added the following statement to the Data Availability section on page 48, lines 1028-1030: ”The data used in this study are part of the pn-TTC project and contain sensitive individual information collected from adolescents. For ethical and privacy reasons, the data cannot be made publicly available.”

ABSTRACT

* Please confirm that your abstract complies with our requirements, including providing all the information relevant to this study type https://journals.plos.org/plosmedicine/s/submission-guidelines#loc-abstract

We reviewed the abstract and confirmed that it meets PLOS Medicine’s requirements.

* Please include the study setting.

We revised the description of the study setting in Methods section on page 3, lines 12-17: “In this study, we analyzed monthly Kessler 6-item Psychological Distress Scale (K6) questionnaire responses collected from 16- to 18-year-old high school students participating in the population-neuroscience Tokyo TEEN Cohort (pn-TTC) in Japan (1,278 responses from 84 participants). Participants included 42 males and 42 females. The pn-TTC is a population-based longitudinal study conducted in Tokyo, Japan that follows children to investigate their developmental and mental health trajectories.”

* We feel that the Abstract Methods section is not sufficiently detailed. Given that most readers will be unfamiliar with the energy landscape analysis, please provide sufficient explanation.

We revised the Abstract Methods section to make the analytical approach clearer to readers unfamiliar with energy landscape analysis. The following description was added on page 3, lines 17-22: “In addition to conventional statistical approaches that summarize multiple questionnaire items into a composite score, we applied “energy landscape analysis”, a method derived from statistical physics that models multivariate psychological states as a dynamic system of interactions among K6 questionnaire items, to visualize longitudinal changes in psychological distress before and during the COVID-19 pandemic (July 2019 to September 2021).”

* How is a healthy state and depressive state defined (scores)?

We clarified the definition of the healthy and depressive states by adding the following description on page 3, lines 23-25: “Here, we define the depressive and healthy states as configurations in which all six K6 items are above or below each participant’s individual mean, respectively.”

* Please quantify the main results (if applicable, with 95% CIs and p values).

We quantified the key findings and revised the manuscript to reflect these results on pages 3-4, lines 25-28: ”Before the pandemic, the healthy state occurred 11.0 times as frequently as the depressive state. In contrast, during the pandemic, the relative frequency of the healthy state increased to 18.2, 18.5, and 15.0 times that of the depressive state, respectively”.

* Please provide the main baseline characteristics of the study population.

We added a description of the main baseline characteristics of the study population as follows on page 3, lines 12-17: “In this study, we analyzed monthly Kessler 6-item Psychological Distress Scale (K6) questionnaire responses collected from 16- to 18-year-old high school students participating in the population-neuroscience Tokyo TEEN Cohort (pn-TTC) in Japan (1,278 responses from 84 participants). Participants included 42 males and 42 females. The pn-TTC is a population-based longitudinal study conducted in Tokyo, Japan that follows children to investigate their developmental and mental health trajectories.”

* Did all participants undergo MRI?

All participants included in the present analysis (i.e., those who completed the K6 questionnaire) also underwent MRI. For details, please refer to the data flow diagram in **Supplementary Figure 1**.

* Please ensure that all numbers presented in the abstract are present and identical to numbers presented in the main manuscript text.

We checked and confirmed that all numbers in the Abstract are consistent with those in the main text.

* In the last sentence of the Abstract Methods and Findings section, please describe the main limitation(s) of the study's methodology.

We added the following sentence to the last part of the Abstract Methods and Findings section on page 4, lines 35-37, to describe the main methodological limitation: “Because all participants lived in Tokyo, generalizability remains limited, and as the association between psychological states and brain development is descriptive, future studies in diverse cohorts are needed to establish causality.”

AUTHOR SUMMARY

* Under ‘What did the researchers do and find?’, we suggest briefly explaining in lay terms what the energy landscape analysis does.

We added the following lay explanation to the “What did the researchers do and find?” section on page 5, lines 52-53: “Energy landscape analysis, a method derived from statistical physics, has the potential to capture the complex dynamics of psychological states.”

INTRODUCTION

*Please remove the study results and/or conclusion from the Introduction.

We deleted the sections of the Introduction that contained study results or conclusions.

*Please conclude the Introduction with a clear description of the study question or hypothesis.

We added the following statement at the end of the Introduction on pages 8-9, lines 122-128, to clearly describe the study hypothesis: “We hypothesize that the energy landscape analysis can characterize how the pattern and stability of adolescents’ psychological states and mental health shifted before and during the pandemic, particularly in response to social restrictions such as school closure. Therefore, this study aims to examine whether the energy landscape analysis appropriately represents the underlying dynamics of adolescent mental health data and to explore how visualizing the distribution of psychological energy can provide insights into the impact of the pandemic on adolescent health.”

METHODS AND RESULTS

* Since most of our readers will not be familiar with energy landscape analysis, we have found that the description of the results can be quite complex. When revising the manuscript, please consider explaining the results in a broadly accessible way, focusing on the most relevant findings.

We revised the Results section to improve clarity and ensure that the key findings are explained in a way that is accessible to readers unfamiliar with energy landscape analysis.

* “In pn-TTC Wave 3” – was wave 3 in 2020?

We clarified the survey period by adding “conducted from May 2018 to September 2020” to the text on page 10, line 144.

* In the description of the study participants, please ensure that it’s 100% clear what data from which time point was included for the analysis in your study focusing on the energy landscape analysis as well as the MRI analysis.

To improve clarity regarding the specific time points and datasets used for both the energy landscape analysis and the MRI analysis, we revised and redesigned **Supplementary Figure 1**. The updated figure provides a clearer and more detailed overview of the study participants and data inclusion criteria.

* “We will release the Python code in the near future.” - Please make any custom code available, either as part of your data deposition or as a supplementary file. Also, please include the statement on code availability in the data availability statement in the online submission form.

We made the Python code used in this study publicly available and have updated the manuscript to include a statement on code availability.

* “Image acquisition and processing” – did the MRI analysis include data from all four waves (2013-2023) or just data from July 2019 to October 2021? Please clarify.

The MRI analysis included data from all four waves (from September 2013 to June 2023). To clarify this point, we added the description “from September 2013 to June 2023” in the revised manuscript on page 10, line 144. In addition, we redesigned **Supplementary Figure 1** to provide a more detailed and transparent overview of the data flow.

* Please clarify whether the pn-TTC received ethical approval prior to commencing the first wave 1 in 2013.

All the pn-TTC measurements in Wave 1 in 2013 were performed after approval from the ethical committee.

* Figure 3: Please define G1 and G2. Please add the definitions for Periods 1-4. Please ensure that you add sufficient detail and explanation in the figure description (as done in Figure 2). Each figure should be self-explanatory on a stand-alone basis.

We updated the figure legends to define G1 and G2 and to include explanations for Periods 1-4. The legends were also revised to provide sufficient detail, ensuring that each figure is self-explanatory on a stand-alone basis.

* Figures: Please define all elements of box plots in the figure caption - center line, box limits and whiskers. Please indicate in the figure caption the meaning of the whiskers in Figure (e.g. 95% CI or SD).

We revised the figure legends to include definitions of all box plot elements, including the center line, box limits, and whiskers, and to specify the meaning of the whiskers.

* Figures: We think it would be useful to briefly explain the meaning of the states 111111 and 000000.

We revised the figure legends to include brief explanations of the states “111111” and “000000” for clarity.

* At times, e.g. ll.550-556, we find that the results section contains too many re-iterations/explanations of methods details. Please ensure that the methods are sufficiently explained in the Methods section and in a way that’s accessible to a broad readership.

We revised the manuscript to reduce reiteration of methodological details in the Results section, ensuring that methods are appropriately and clearly described in the Methods section for a broad readership.

* Please consider avoiding the use of red and green in order to make your figure more accessible.

We adjusted the color schemes in the figures to minimize the use of red and green where possible, thereby improving accessibility for all readers.

* Figure 4: Please define G1 and G2. For graphs C-H, please mention the type of mention the type of plot.

We updated Figure 4 to include definitions of G1 and G2 and to clearly indicate the type of plots used in panels C-H for clarity.

DISCUSSION

* “disturbing reports from Japan” – please avoid using emotive language.

We have replaced the phrase “disturbing reports from Japan” with “reports from Japan” to ensure neutral and objective language.

**General Editorial Requests:**

1) We ask every co-author listed on the manuscript to fill in a contributing author statement. If any of the co-authors have not filled in the statement, we will remind them to do so when the paper is revised. If all statements are not completed in a timely fashion this could hold up the re-review process. Should there be a problem getting one of your co-authors to fill in a statement we will be in contact. YOU MUST NOT ADD OR REMOVE AUTHORS UNLESS YOU HAVE ALERTED THE EDITOR HANDLING THE MANUSCRIPT TO THE CHANGE AND THEY SPECIFICALLY HAVE AGREED TO IT.

We understand the instructions and are prepared to address any issues or requests from the editors if they arise.

2) Please ensure that the paper adheres to the PLOS Data Availability Policy (see http://journals.plos.org/plosmedicine/s/data-availability), which requires that all data underlying the study's findings be provided in a repository or as Supporting Information. For data residing with a third party, authors are required to provide instructions with contact information for obtaining the data. PLOS journals do not allow statements supported by "data not shown" or "unpublished results." For such statements, authors must provide supporting data or cite public sources that include it.

We have updated the Data Availability Statement to explain why the TTC data cannot be publicly shared. The following text was added on page 48, lines 1028-1030: “The data used in this study are part of the pn-TTC project and contain sensitive individual information collected from adolescents. For ethical and privacy reasons, the data cannot be made publicly available”.

3) Please review your reference list to ensure that it is complete and correct. If you have cited papers that have been retracted, please include the rationale for doing so in the manuscript text, or remove these references and replace them with relevant current references. Any changes to the reference list should be mentioned in the rebuttal letter that accompanies your revised manuscript.

We have carefully reviewed the reference list to confirm its completeness and accuracy and will promptly address any issues if they are identified during the editorial process.
