# Supplementary material for: Psychological distress among Japanese high school students during the COVID-19 pandemic: An energy landscape analysis
Source: PLoS Med. 2026 Jan 22;23(1):e1004884. doi: 10.1371/journal.pmed.1004884 (PMC12826503; doi:10.1371/journal.pmed.1004884)
Supplement: Attachment — Submitted filename: PBP_revision3_final.docx [file pmed.1004884.s041.docx]

**Response to editor and reviewer comments**

Daiki Tatematsu, Naotoshi Nakamura, Masato Abe, Tetsuo Ishikawa, Takahiro Ezaki, Lin Cai, Eiryo Kawakami, Kazuyuki Aihara, Atsushi Nishida, Naohiro Okada, Naoki Masuda, Kiyoto Kasai, Shinsuke Koike, Shingo Iwami

December 18, 2025

We would like to thank the reviewers for their thorough reading of the manuscript and their helpful comments and suggestions. In this document, we present the reviewers’ comments in black and provide our responses in red. All changes made in response to the reviewers’ comments in PMEDICINE-D-24-03134R4 have been incorporated in the revised version of the manuscript. We believe that the manuscript has been considerably improved by these changes. All co-authors have agreed with these changes.

**Comments from Reviewers:**

Reviewer #2: The authors have addressed all comments comprehensively, and the manuscript now represents a robust and valuable contribution to the field. I am pleased to recommend this work for publication and look forward to its positive impact. I have only a few minor suggestions that may further strengthen the manuscript:

**Reply to Reviewer #2:**

We sincerely thank the reviewer for the positive and encouraging evaluation of our manuscript and for the recommendation for publication. We are grateful for the recognition that the revised manuscript now constitutes a solid and valuable contribution to the field. We also appreciate the helpful minor suggestions, all of which we carefully addressed and incorporated into the manuscript as detailed below. *We particularly appreciated your insightful and rigorous statistical comments, which we found highly constructive and genuinely enjoyable to address.*

1) Consider including a minimal predictive or cross-validation analysis, or more clearly acknowledging this limitation.

**Reply to comment 1):**

We added the absence of predictive or cross-validation analyses as a limitation of the present study in the Discussion. Specifically, we added the following statement on page 39, lines 730-733:

*“Fourth, the present study was not designed to perform predictive modeling or cross-validation analyses. Accordingly, the robustness of the identified energy landscape structures in terms of out-of-sample prediction was not formally evaluated and should be examined in future studies, ideally using independent cohorts.”*

2) Summarize key LCA model indicators (e.g., fit statistics, class sizes) in the Results section to complement the supplementary figures.

**Reply to comment 2):**

We added the numerical values of the class sizes (proportion of data points), which are central to our interpretation of the latent class analysis results, to **Supplementary Note 3** to provide a clearer quantitative summary that complements **Supplementary Fig 11B**.

3) Ensure that all interpretations remain appropriately cautious, particularly regarding causal language.

**Reply to comment 3):**

We revised the manuscript to ensure that interpretations remain appropriately cautious, and we replaced potentially causal wording with non-causal language (e.g., “associated with”) where appropriate.

4) Provide a slightly expanded intuitive explanation of the energy landscape approach to support accessibility for non-technical readers.

**Reply to comment 4):**

To improve accessibility for non-technical readers, we added the following intuitive explanation to the **Introduction** on page 8, lines 121-125:

*“Rather than treating psychological distress as a static score that is simply ‘high’ or ‘low,’ the energy landscape analysis captures the dynamic nature of mental states over time. This perspective focuses on the stability and variability of psychological states, providing an intuitive way to understand how mental health fluctuates and how individuals move between healthier and more distressed conditions.”*

**Requests from Editors:**

* Please note that the title should include the study design (i.e., after a colon). Editorial suggestion: Psychological distress among Japanese high school students during the COVID-19 pandemic: An energy landscape analysis

The title was revised to explicitly include the study design and now reads: “Psychological distress among Japanese high school students during the COVID-19 pandemic: An energy landscape analysis”.

* Abstract: “The evolving energy landscape revealed that the pandemic reduced the likelihood of being in a depressive state.”- We suggest moving the sentence after ‘….healthy state increased to 18.2, 18.5, and 15.0 times that of the depressive state, respectively”.

The sentence was moved to the suggested position in the **Abstract**, and it was also rephrased to avoid causal language in response to comment 3) from Reviewer #2. It now reads: “The evolving energy landscape revealed an association between the pandemic period and a lower likelihood of being in a depressive state.”

* Please define t, p, and q the first time used (‘caudal part of the middle frontal gyrus (cMFG) (t=-2.36, p=0.019, q=0.048)).

We defined the statistical terms at their first occurrence by explicitly stating that t denotes the t-statistic, p the p-value, and q the q-value. The manuscript was revised accordingly.

* Please include the statement on code (“The programming code we used to calculate the energy landscape is a Python version of the MATLAB code available from [30] along with a tutorial. The Python code is available on GitHub (https://github.com/ttttmmttddiikk/Energy_Landscape_Analysis) and Zenodo (DOI: 10.5281/zenodo.17585043).”) in Data Availability statement in the online submission form.

We added the statement regarding the availability of the programming code to the **Data Availability statement** in the online submission form.

* Regarding comment 1) from Reviewer #2: While adding a minimal predictive or cross-validation analysis would be beneficial, the editorial team would accept if you acknowledged this as a limitation.

As suggested, we explicitly acknowledged the lack of a predictive or cross-validation analysis as a limitation of the present study in the **Discussion**.
